# Electronic properties and circuit applications of networks of electrochemically exfoliated 2D nanosheets

**DOI:** 10.1038/s41467-025-64100-y

**Published:** 2025-10-10

**Authors:** Tian Carey, Kevin Synnatschke, Goutam Ghosh, Luca Anzi, Eoin Caffrey, Emmet Coleman, Changpeng Lin, Anthony Dawson, Shixin Liu, Rebekah Wells, Mark McCrystall, Jan Plutnar, Iva Plutnarová, Joseph Neilson, Nicola Marzari, Laurens D. A. Siebbeles, Roman Sordan, Zdenek Sofer, Jonathan N. Coleman

**Affiliations:** 1 School of Physics, CRANN & AMBER Research Centres, Trinity College Dublin, Dublin, Ireland; 2 Chemical Engineering Department, Delft University of Technology, Delft, The Netherlands; 3 L-NESS, Department of Physics, Politecnico di Milano, Como, Italy; 4 Theory and Simulation of Materials, and National Centre for Computational Design and Discovery of Novel Materials, École Polytechnique Fédérale de Lausanne, Lausanne, Switzerland; 5 Department of Inorganic Chemistry, University of Chemistry and Technology Prague, Prague, Czechia

**Keywords:** Electronic devices, Electrical and electronic engineering

## Abstract

High aspect-ratio 2D materials are promising for solution-processed electronics, yet the factors controlling exfoliation remain unclear and relatively few solution-processed networks have been electrically characterized. Here we combine theory and experiment to show that electrochemical exfoliation of layered crystals with sufficient stiffness-anisotropy (in-plane/out-of-plane Young’s modulus ratio >1.7) yields high aspect-ratio nanosheets with intrinsic mobilities *μ*_NS_ = 20–75 cm²V⁻¹s⁻¹ across transition metal dichalcogenides and related alloys. Impedance spectroscopy indicates that solution-deposited networks can achieve junction-to-nanosheet resistance ratios (R_J_/R_NS_) as low as ~3, supporting theoretical predictions that *μ*_NS_/*μ*_Net_ = R_J_/R_NS_ + 1 and suggesting that further reductions in R_J_ will increase μ_Net_ toward the nanosheet limit (*μ*_NS_). These networks display n-type, p-type, and ambipolar behaviour, with on/off ratios up to 10⁵ and mobilities *μ*_Net_ = 13 cm²V⁻¹s⁻¹. Here, we show that such high-performing 2D materials enable functional solution-processed circuits, including inverters, buffers, a 4-bit digital-to-analog converter, and a circuit capable of encoding and decoding 7-bit ASCII messages.

## Abstract (web summary)

2D semiconductors hold promise for solution-processed circuits requiring low-cost components and manufacturing scalability. Here, the authors investigate the criteria for the electrochemical exfoliation of high aspect-ratio nanosheets from 28 different layered materials, identifying the most promising candidates and key bottlenecks for solution-processed complementary electronics and functional circuits.

## Introduction

Solution-processed electronics are important for circuits requiring low-cost components and manufacturing scalability (m^2^/min) on a wide range of non-conformal substrates (e.g. textile and polymers)^1,2^. Two-dimensional (2D) semiconducting nanosheets show great promise in this area due to their solution-processability^3,4^, diversity^5^, mechanical flexibility^6^, and impressive intrinsic electrical properties (e.g. nanosheet mobility, *µ*_NS_ > 50 cm^2 ^V^−1^ s^−1^)^7^. Liquid-phase exfoliation^8,9^ (LPE) has yielded many types of semiconducting nanosheets with low aspect ratio (AR < 30)^8^. These have been solution-processed into electronic devices with poor performance, displaying network mobilities, *μ*_Net_ ∼ 0.1 cm^2 ^V^−1 ^s^−1^ and current on/off ratio, *I*_on_/*I*_off_ ~10^2^, significantly below expectations for individual nanosheets^7,10^. Conversely, electrochemical exfoliation^4,11,12^ (EE) is a method known to produce high AR nanosheets^13–15^, leading to high mobility networks (*μ*_Net_ up to 15 cm^2 ^V^−1 ^s^−1^)^4,16–18^, due to the link between high AR and low junction resistance (*R*_J_)^19^. In addition, transition metal phosphorus trichalcogenides (M-P-X_3_, TMTs)^20^, transition metal monochalcogenides (M-X, TMMs)^13^ and elemental materials^21^ have been electrochemically exfoliated, although the electrical performance of their networks is unknown. Moreover, the factors determining successful exfoliation remain unclear in general.

The *AR* of nanosheets produced by LPE depends on the balance between in-plane tearing energy and out-of-plane peeling energy^8^. In EE, it is expected that ion insertion lowers the peeling energy, aiding exfoliation and maximising AR. Increased AR has been linked to the formation of conformal, low-resistance junctions within networks^19^. However, the influence of the crystal’s mechanical properties on the length (*L*) and thickness (*t*) of electrochemically exfoliated nanosheets is unstudied. We believe a deeper understanding of the physics of the exfoliation process could facilitate nanosheet optimisation, increase ARs, and so minimise *R*_J_^19,22^.

For networks, theory predicts^22^ that ${\mu }_{{\mathrm{NS}}}/{\mu }_{{\mathrm{Net}}}\approx {R}_{J}/{R}_{{\mathrm{NS}}}+1$. Thus, reducing *R*_J_ below the resistance of the individual nanosheets (*R*_NS_) would result in *μ*_Net_ approaching its upper limit of *µ*_NS_^22^, opening the way toward high-performance solution-processed devices. However, for most electrochemically exfoliated nanosheet types, the actual values of *µ*_NS_ are unknown, as are the values of *R*_J_ in solution-processed nanosheet networks. This means that for most types of 2D materials, we have no idea what the performance limits are or how far we are from achieving them.

Furthermore, although significant progress has been made recently in solution-processed logic with complementary functionality^23–26^ and resistive random access memory^27,28^, we still lack important solution-processed circuits, such as digital-to-analogue converters (DACs), commonly used in microcontrollers and systems-on-chip to convert digital signals into continuous analogue signals, and binary-amplitude-shift-keying (BASK) circuits, used in digital communication and signal processing^29^. The development of solution-processed, 2D-based DACs and BASK circuits would enable the next significant step in solution-processed circuits.

Using known exfoliation protocols, we combine theory and experiment to explore the underlying mechanisms of crystal expansion in a range of materials. For each exfoliated material, we solution-deposit nanosheet networks, performing electrical, optical and transistor characterisation. For a subset of materials, we examine the factors limiting charge transport in these networks by measuring *R*_J_, *R*_NS_ and *μ*_NS_ and using a network model to link these values to *μ*_Net_. Finally, we use our best performing transistors to fabricate solution-processed resistor ladder networks, inverters and buffer circuits.

### Results: selection of two-dimensional material families

**Fig. 1:**
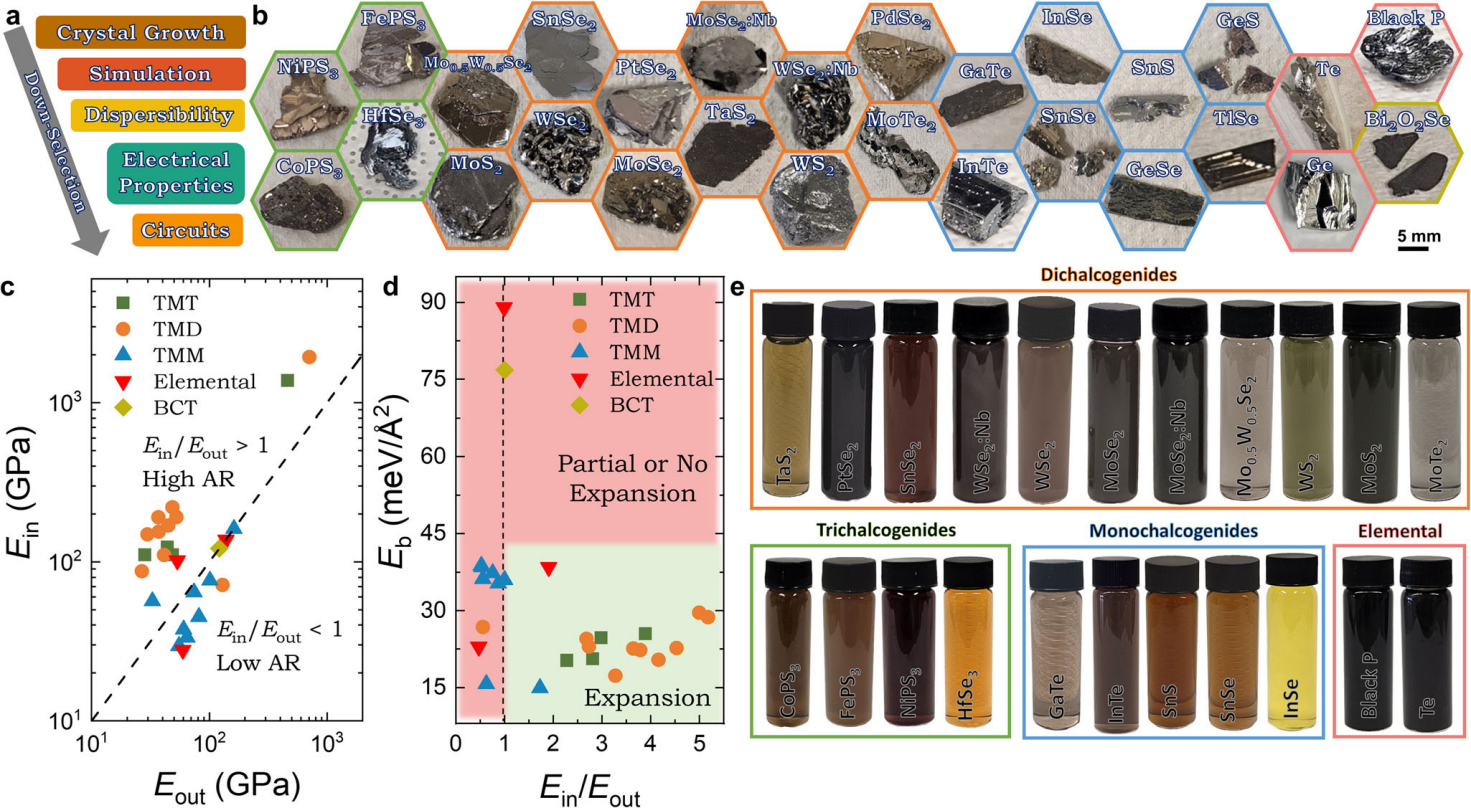
Material families and mechanical criteria for exfoliation. **a** Schematic of the systematic down-selection process for identifying low-bandgap 2D materials for solution-processed circuits. **b** Optical images of various crystals examined in the study, highlighting different families of 2D materials with corresponding colour-coded outlines with transition metal trichalcogenides (TMT, green), transition metal dichalcogenides (TMD, orange), transition metal monochalcogenides (TMM, blue), elemental materials (red), and bismuth oxychalcogenides (BCT, yellow). **c** Plot of in-plane (*E*_in_) versus out-of-plane (*E*_out_) Young’s moduli for each material, with a dashed line indicating *E*_in_/*E*_out_ = 1. **d** Correlation between simulated mechanical properties (*E*_in_/*E*_out_ and interlayer binding energy, *E*_b_) and experimentally determined intercalation success. **e** Semiconducting and metallic inks after dispersion in N,N dimethylformamide with polyvinylpyrrolidone (DMF-PVP).

We initially chose 28 low optical bandgap (*E*_Op_ < 2 eV) 2D materials, a group which will be subjected to a rigorous down-selection process to identify champion materials for solution-processed circuits (Fig. 1a). Figure 1b shows optical images of the 2D crystals grown by us and which can be grouped into five families: TMMs, TMDs, TMTs, layered bismuth oxychalcogenides (BCT) and elemental materials (metalloids).

The TMMs are outlined in blue and are combinations of a chalcogen (S, Se or Te) with a group 13 (In, Ga, Tl) or group 14 (Sn, Ge) element. These elements are post-transition metals except for Ge, which is a metalloid. The TMDs, outlined in orange, combine one transition metal (W, Mo, Pt, Pd, Ta) or post-transition metal (Sn) and two chalcogen atoms (S, Se). We will also examine the alloy, Mo_0.5_W_0.5_Se_2_, as well as substitutional doping of MoSe_2_ and WSe_2_ with niobium (MoSe_2_:Nb, WSe_2_:Nb) to induce p-type doping^30^. TMTs (green outline) such as CoPS_3_, FePS_3_, NiPS_3_ and HfSe_3_ will also be examined. We also examine elemental 2D materials of tellurene (Te), germanene (Ge), and black phosphorus (BP), which are shown with a red outline. Finally, BCTs such as bismuth oxyselenide Bi_2_O_2_Se (yellow outline) have shown great promise as high electron mobility materials (>100 cm^2 ^V^−1^ s^−1^) and are also included^31^.

### Mechanical criteria for electrochemical exfoliation

To identify the factors controlling the electrochemical exfoliability of 2D materials, we use density functional theory to calculate the in-plane (*E*_in_) and out-of-plane (*E*_out_) Young’s moduli as well as the inter-sheet binding energy, *E*_b_, as described in Supplementary Note 2. We use, the ratio of *E*_in_/*E*_out_ as a figure of merit for determining crystal expansion. In Fig. 1c, we plot the *E*_in_ and *E*_out_ for each material, where the dashed line represents where *E*_in_/*E*_out_ = 1. Alternatively, it was previously proposed that layered crystals with *E*_b_ < 120 meV Å^−2^ are potentially exploitable^32,33^. We use the Materials Cloud two-dimensional crystals database to determine the *E*_b_ of each material (Supplementary Note 2). We find that every crystal in this study has *E*_b_ < 90 meV Å^−2^, suggesting that all of our 2D crystals are potentially exfoliable.

To assess the potential of *E*_in_/*E*_out_ and *E*_b_ as metrics for electrochemical exfoliability, we have performed EE on each material^11,34^. We apply an electrical potential (8 V) to each crystal immersed in the electrolyte tetrapropylammonium bromide (TPA^+^Br^−^) (See “Methods”). Ideally, the intercalation of the TPA^+^ cations swells the crystal, weakening the interlayer bonding, facilitating exfoliation by mild sonication. We emphasise that crystal expansion is a required prerequisite for exfoliation. In Fig. 1d, we plot the calculated values of *E*_in_/*E*_out_ and *E*_b_ as a phase diagram with the presence or absence of swelling indicated (green zone for swelling). We found expansion to occur for all crystals with *E*_in_/*E*_out_ > 1.7, while for crystals with *E*_in_/*E*_out_ < 1 limited expansion was observed, leading to very poor exfoliation (<0.01 mg nanosheets produced). However, while expansion could be correlated with *E*_b_ < 40 meV Å^−2^, this criterion was not enough as crystals with *E*_in_/*E*_out_ < 1.7 showed limited or no expansion, indicating that *E*_in_/*E*_out_ > 1.7 is a more basic requirement. Crystal interlayer spacing is uncorrelated with exfoliation efficiency (Supplementary Note 15). The expanded crystals were then liquid-exfoliated as described in the methods and then formulated into isopropyl alcohol (IPA) based inks as shown in Fig. 1e. Optical absorption spectra for each ink are obtained using an integrating sphere and display the expected excitonic transitions further described in detail in Supplementary Note 8–12^35^.

**Fig. 2:**
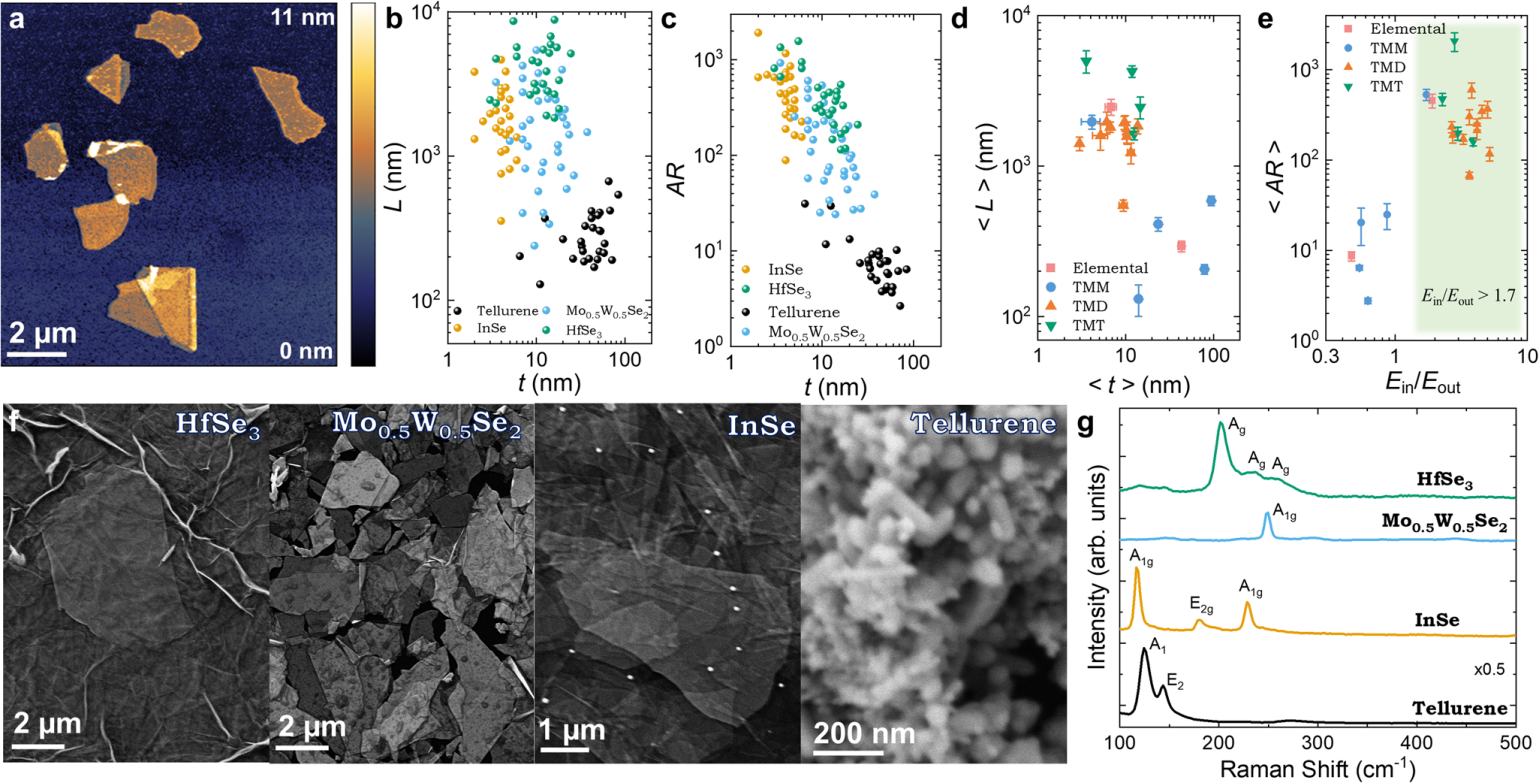
Nanosheet dimensions and formation of solution-processed networks. **a** Atomic force microscopy (AFM) micrograph showing InSe nanosheets drop-cast onto a Si/SiO_2_ substrate. Nanosheet lateral size, *L*, **b** and aspect ratio, *AR*, (**c**) plotted versus apparent nanosheet thickness, *t*, for four selected nanosheet types. Each data point represents a single nanosheet. **d** Relationship between average nanosheet lateral size (<*L*>) and apparent thickness (<*t*>) for each material under study. In (**d**), the green region illustrates *E*_in_/*E*_out_ > 1.7. The data have been grouped into families of materials: TMT, TMD, TMM, and elemental materials. **e** Dependence of mean nanosheet *AR* for each material on the starting crystal’s *E*_in_/*E*_out_ ratio. The data has been grouped into material families, as shown in (**d**). Errors in (**d**) and (**e**) are the standard deviation of the mean, SDOM. **f** Scanning electron microscopy images showing the morphology of solution-deposited nanosheet networks prepared using nanosheets from each material family. These images show alignment and connectivity for high *AR* nanosheets and the presence of nanosheets in the tellurene network. **g** Raman spectra of selected 2D materials showing characteristic vibrational modes, indicating the phase and quality of exfoliated nanosheets.

High AR nanosheets are critical for solution-processed electronics^19^. Atomic force microscopy (AFM) images such as those in Fig. 2a allow the measurements of *L*, *t* and *AR* for individual nanosheets. Examples of *L-t* and *AR*-*t* data clouds are given for one material from each family in Fig. 2b, c. Unlike LPE nanosheets^8^, there appears to be no *L*-*t* correlation within each material (Supplementary Note 14). The mean nanosheet length and apparent thickness are plotted for each material in Fig. 2d (Supplementary Note 3). Most materials have $\langle L\rangle > 1\,{\mathrm{\mu m}}$ and $\langle t\rangle < 20\,{\mathrm{nm}}$, although some materials are smaller and thicker (*L* < 1 µm, *t* < 100 nm). To probe the relationship between nanosheet dimensions and crystal properties, we plot $\langle AR\rangle$ versus ${E}_{{\mathrm{in}}}/{E}_{{\mathrm{out}}}$ in Fig. 2e. This shows clear evidence that crystals with stiffness anisotropy above a critical value, ${E}_{{\mathrm{in}}}/{E}_{{\mathrm{out}}} > 1.7$, yield nanosheets with *AR* > 100, underlining the importance of crystal mechanical properties.

High ARs are required for nanosheets to form aligned films with conformal junctions as explained by Kelly et al.^19^. Figure 2f shows examples of scanning electron microscopy images of solution-deposited nanosheet networks of materials that have not previously been electrochemically exfoliated for transistor device applications (Supplementary Note 14). High-*AR* materials such as InSe, Mo_0.5_W_0.5_Se_2_ and HfSe_3_ all have networks of well-aligned nanosheets with highly-connected junctions, requirements for low *R*_J_^19^. In contrast, Te is a low-*AR* material and forms a much more disordered, particle-like network. Example Raman spectra are shown in Fig. 2g and are consistent with the expected semiconducting material phase and dimensions as described in detail in the Supplementary Note 4–7, 14, alongside information on the full characterisation of each material.

### Network conductivity and transistor performance

Although nanosheet inks can be formed into networks in various ways, Langmuir–Schaefer (LS) coating is an efficient way to produce high-quality nanosheet networks. This process leverages the interfacial tension at a hexane/water interface to form highly aligned nanosheet networks with minimal (∼120 μL) use of ink, as detailed in the Methods. We use this technique to fabricate networks (thickness *t*_NET_ ~19–50 nm, Supplementary Note 17) of 22 different nanosheets types on flexible polyethylene terephthalate (PET) substrates for electrical characterisation. Depending on the material, we either evaporated gold top electrodes or used pre-patterned electrode arrays (See “Methods”). In all cases, networks are annealed at 120 °C for 1 h in an inert N_2_ environment.

**Fig. 3:**
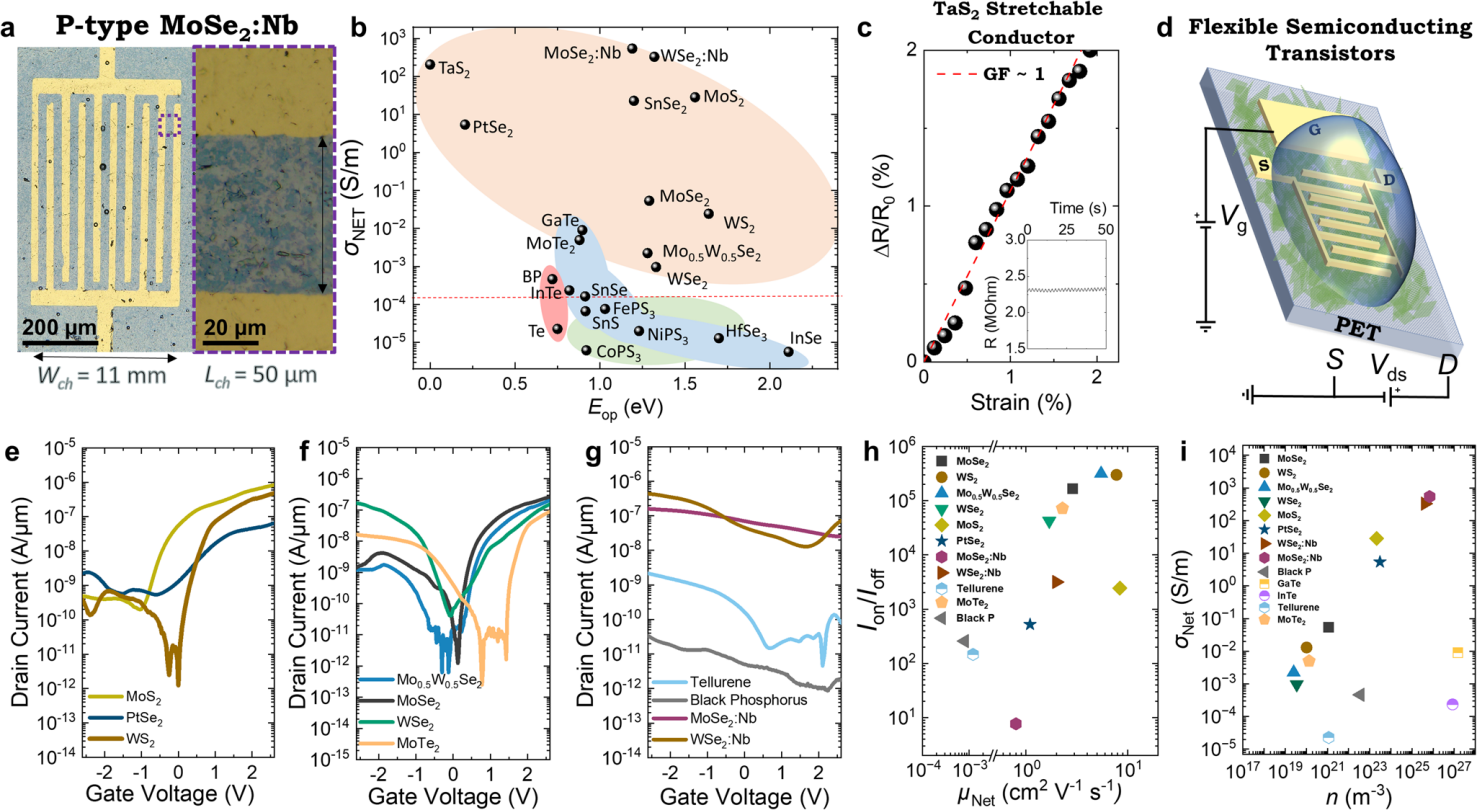
Conductivity and electrical behaviour of nanosheet networks. **a** Optical images showing a nanosheet network fabricated via Langmuir–Schaefer (LS) coating on polyethylene terephthalate (PET) substrates pre-patterned with gold contacts for source, drain, and gate electrodes. Channel width (*W*_ch_) and channel length (*L*_ch_) are indicated. **b** Network conductivity (*σ*_Net_) plotted as a function of optical bandgap (*E*_Op_) for different material families. The horizontal dashed line indicates the conductivity threshold below which networks could not be electrochemically gated. **c** Fractional resistance change (ΔR/R_0_) plotted versus strain for a TaS_2_ network, demonstrating linear scaling with very small slope. The gauge factor (GF), defined as Δ*R*/*R*_0_/*ε*, where *ε* is strain, is low ~1, indicating near strain-independent resistance. Inset: The resistance is almost invariant to a sawtooth applied strain (varying from 0% to 2% to 0% at 0.5 Hz). **d** Schematic of the flexible electrochemically gated transistor on PET. The device consists of source (S), drain (D), and gate (G) electrodes. A drain–source voltage (*V*_ds_) is applied between the source and drain, while a gate voltage (*V*_gs_) is applied to the gate to modulate the channel conductivity. Electrochemically switched transfer characteristics of various networks indicating n-type (**e**), ambipolar (**f**) and p-type (**g**) behaviour. Summary of the performance of electrochemically gated solution-processed transistors studied in this work, showing comparison of network mobility (*μ*_Net_*)* and on/off current ratio (*I*_on_/*I*_off_) (**h**) as well as network conductivity (*σ*_Net_) and charge carrier density (*n*) for each network (**i**). Not shown in (**f**) are data for GaTe and InTe networks due to their extremely small mobility (<10^−7 ^cm^2 ^V^−1^ s^−1^) and on/off ratio (<2).

Figure 3b plots *σ*_Net_ for each material and we estimate *E*_Op_ via Tauc plots with absorption spectra (Supplementary Note 13). With the exception of the Nb-doped TMDs and MoS_2_ and SnSe_2_, materials with higher *E*_Op_ tended to have lower *σ*_Net_, indicating the importance of thermal carrier generation^36^. For example, despite the quality of their junctions (Fig. 2f), HfSe_3_ and InSe have low conductivity due to their high bandgap. Otherwise, the spread in data implies a range of network mobilities from material to material. Broadly, TMDs are the most conductive (*σ*_Net_ > 10^−3 ^S/m) and TMTs the least conductive (*σ*_Net_ < 10^−4 ^S/m), possibly due to TMTs possessing anisotropic nanosheet electrical properties^37^. Similarly, the elemental nanosheet networks of BP and tellurene are anisotropic, resulting in *σ*_Net_ < 10^−3 ^S/m^38,39^. In materials with anisotropic electrical properties, charge carriers must traverse directions with lower conductivity, which increases the effective resistance of the network and lowers *σ*_Net_.

Many applications in flexible electronics will require strain-independent electrical properties. We demonstrate this for an LS-deposited TaS_2_ network in Fig. 3c, finding a minimal resistance change of only ~1% per % of strain, i.e $\varDelta R/{R}_{0}\approx \varepsilon$. Because the piezoresistive response of any material (resistivity, *ρ*, Poisson ratio *ν*) is given by ref. ^40^, $\varDelta R/{R}_{0}=(\varepsilon /\rho ){{\rm{d}}}\rho /{{\rm{d}}}\varepsilon+(1+2\nu )\varepsilon$, this implies that these networks have near strain-independent resistivity (${{\rm{d}}}\rho /{{\rm{d}}}\varepsilon \sim 0$) and *ν* ≈ 0, properties that are near ideal for strain-invariant devices. We demonstrate this by applying a cyclic strain to the TaS_2_ network (Fig. 3c, inset), finding only a very small ripple in the film resistance. It is likely that this behaviour is a feature of all EE nanosheet networks, provided that the networks have similar structure, dimensionality and inter-nanosheet junctions^41^.

To further characterise the electronic properties of our semiconducting networks, we fabricated electrochemical transistors, as shown schematically in Fig. 3d. We used a gold side gate for the electrodes and 1-ethyl-3-methylimidazolium bis(trifluoromethylsulfonyl)imide (EMIM TFSI) to switch the channel^42^. The electrical characterisation is performed using a probe station in ambient air under atmospheric conditions. We sweep a gate voltage, *V*_G_, from 3 V to −3V and measure the transfer characteristics for each network at a drain-source voltage of *V*_DS_ = 1 V. Networks with *σ*_Net_ < 10^−4 ^S/m (shown by the dashed red line in Fig. 3b) could not be gated since their *σ*_Net_ was lower than the ionic conductivity of EMIM TFSI, making it impossible to distinguish between semiconductor modulation or electrolyte effects. For all other materials, we observed ionic gate currents (*I*_G_ ∼ 10^−5 ^A, 5.2 × 10^−9 ^A/μm, matching expected values from the literature)^43^ below the measured drain current (*I*_D_), confirming the drain current modulation to be due to carrier concentration changes within the semiconducting channel (Supplementary Note 18).

We observe n-type behaviour for PtSe_2_, WS_2_, and MoS_2_, which is consistent with previous reports (Fig. 3e)^44,45^. The networks of MoSe_2_, WSe_2_, MoTe_2_, and Mo_0.5_W_0.5_Se_2_ display ambipolar behaviour, in line with expectations (Fig. 3f)^6,46^. Moreover, we observe p-type behaviour for MoSe_2_:Nb, WSe_2_:Nb, tellurene and BP. The addition of four p-type networks is particularly noteworthy as there are relatively few p-type 2D solution-processed networks in the wider literature (Supplementary Note 29). P-type behaviour was also observed for GaTe and InTe, although at extremely low *μ*_Net_ (Supplementary Note 16).

Figure 3h shows a summary of the average *μ*_Net_ and *I*_on_/*I*_off_ for our electrochemically gated transistors, with the best performing transistors found at the top right-hand corner of the plot. We obtain average *μ*_Net_ in the range of 1–8 cm^2 ^V^–1^ s^–1^ and average peak mobilities *μ*_peak_ > 10 cm^2 ^V^–1^ s^–1^ for TMDs with *I*_on_/*I*_off_ in the range of 10^0^–10^5^ (Supplementary Note 17). In some cases, relatively high off-currents were found as is often observed during electrochemical gating. The best performing family was the TMDs, where WS_2_, WSe_2_:Nb and Mo_0.5_W_0.5_Se_2_ had complementary n-type, p-type and ambipolar properties, respectively. Substitutional doping proved a good strategy to convert the ambipolar materials, WSe_2_ and MoSe_2_, to p-type while retaining reasonable *μ*_Net_.

In Supplementary Note 29, we have compiled a comprehensive list of state-of-the-art solution processed 2D semiconducting networks and their performance to date. We stress the realisation of network-based transistors with materials that have been rarely applied for solution-processed electronics: WMoSe_2_, MoTe_2_, tellurene, InTe, GaTe, MoSe_2_:Nb and WSe_2_:Nb, while demonstrating high *μ*_Net_ and *I*_on_/*I*_off_ for some of them. Our devices show *μ*_Net_ and *I*_on_/*I*_off_ comparable to some of the best organic polymers^47^, carbon nanotubes^48^ and metal oxides^49^ (*μ*_Net_ ∼ 10 cm² V⁻¹ s⁻¹ with *I*_on_/*I*_off_ < 10^5^)^6^. Furthermore, our *μ*_Net_ and *I*_on_/*I*_off_ are higher than what has been achieved for LPE semiconducting transistors, typically *μ*_Net_ ∼ 0.1 cm^2 ^V^–1^ s^–1^ with *I*_on_/*I*_off_ < 10^3^ ^50,51^. While our devices are comparable to the best EE networks *μ*_Net_ ∼ 10 with *I*_on_/*I*_off_ < 10^6^, we demonstrate a much broader range of materials with complementary electrical properties. The majority of the literature has only examined MoS_2_ networks for transistors, with a few exceptions beyond it^18,25,52,53^. We attribute the high *μ*_Net_ and *I*_on_/*I*_off_ to our use of crystals with favourable mechanical properties (*E*_in_/*E*_out_ > 1.7), which enables EE of high *AR* > 10^2^–10^3^ nanosheets leading to networks with low *R*_J_^22^. Our devices represent a significant broadening in the library of complementary 2D nanosheets available for solution-processed circuits.

We can combine the network conductivity reported in Fig. 3b with the mobility values in Fig. 3h to obtain carrier densities (*σ*_Net_ = *nqμ*_Net_) as shown in Fig. 3i. We find very high carrier densities in the Nb-doped materials, but also in MoS_2_ and PtSe_2_. In PtSe_2_, this can be attributed to the low bandgap. However, in MoS_2_, this suggests a significant degree of inadvertent doping and explains its position in Fig. 3b as well as its high *I*_off_. The *n*-values found are as expected and we explain their positions relative to each other in Supplementary Note 20.

### Junction resistance governs network mobility

**Fig. 4:**
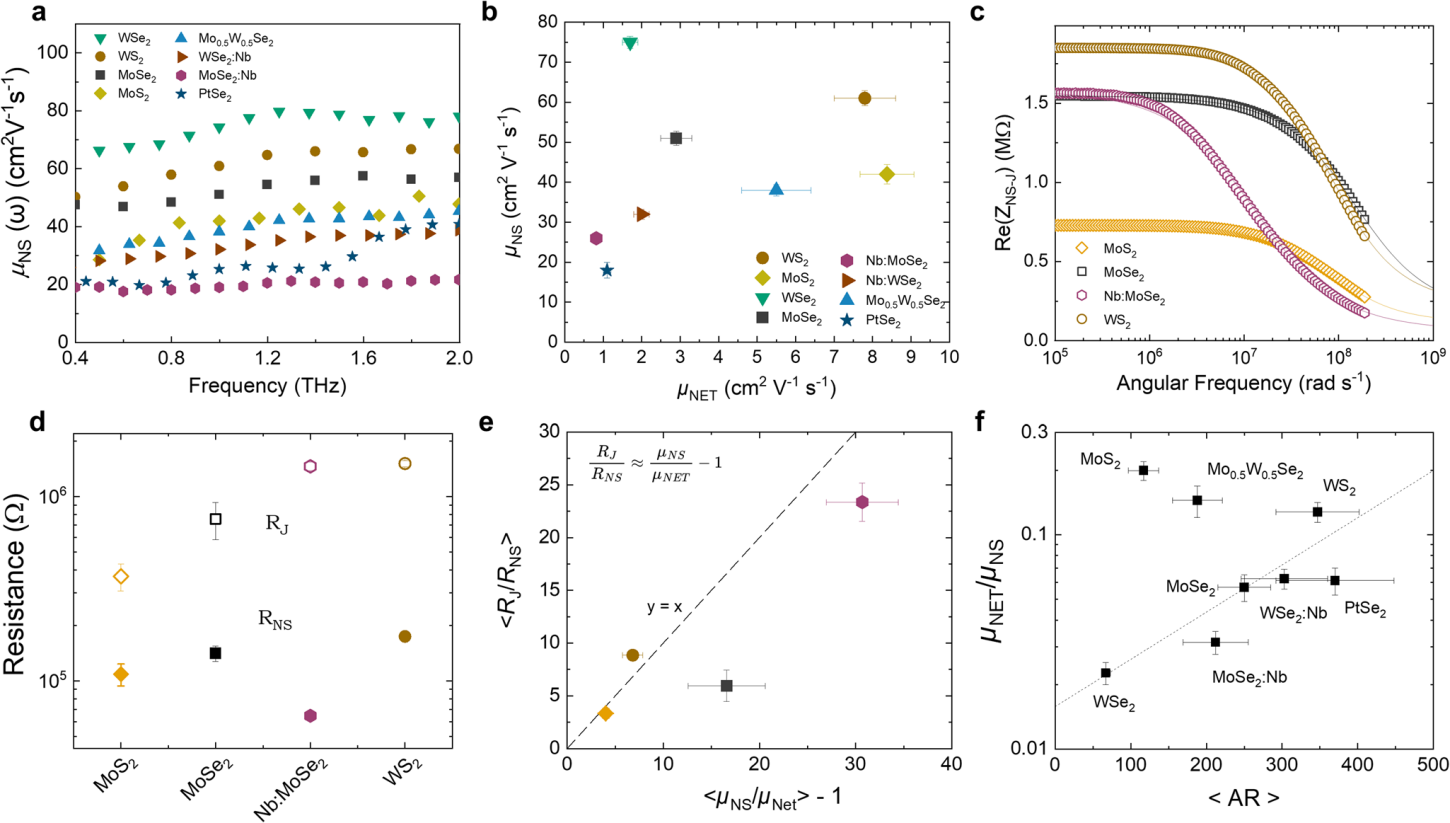
Nanosheet and network mobility with junction resistance analysis. **a** Nanosheet mobility (${\mu }_{{\mathrm{NS}}}\left(\omega \right)$) found from THz spectroscopy as a function of radian frequency $(\omega /2\pi )$ of the THz electric field for a range of different nanosheet types. **b** Nanosheet mobility (*μ*_NS_) found from THz spectroscopy plotted as a function of network mobility (*μ*_Net_) shown in Fig. 3h. Errors are found by SDOM over five independent devices. **c** Real impedance spectra measured on TMD networks. These spectra are scaled to represent the real impedance of single (average) nanosheet-junction pairs. The solid lines represent fits to an equivalent circuit model. **d** The junction (*R*_J_) and nanosheet resistance (*R*_NS_) were extracted from the data in **c** for each material examined. Errors are found by SDOM over five indep**e**ndent devices. **e** The relation between *R*_J_/*R*_NS_ ratio and the ratio of nanosheet to network mobility (*µ*_NS_/*µ*_Net_). The dashed line represents the expected behaviour (see text). Error bars are found by fractional propagation of errors. **f** The ratio of *μ*_Net_/*μ*_NS_ plotted versus mean aspect ratio. The dashed line is a guide to the eye. Errors are found by SDOM over five independent devices combined with error propagation.

Modelling^22^ shows that *µ*_Net_ and *R*_J_ are linked in nanonetworks via ${\mu }_{{\mathrm{NS}}}/{\mu }_{{\mathrm{Net}}}\approx {R}_{J}/{R}_{{\mathrm{NS}}}+1$. This means that the network mobility can be increased by reducing ${R}_{J}/{R}_{{\mathrm{NS}}}$. In addition, it shows that *µ*_Net_ can never exceed *µ*_NS_, with this maximum value realisable by achieving *R*_*J*_«*R*_NS_^22^. Thus, knowledge of *µ*_NS_ is important to know the upper mobility limit, while knowledge of ${R}_{J}/{R}_{{\mathrm{NS}}}$ is needed to know how far a network is from this limit. Here, we use optical-pump terahertz-probe spectroscopy and time-resolved terahertz spectroscopy (TRTS) on a select number of promising 2D EE networks to determine the nanosheet mobility, *µ*_NS_ (Supplementary Note 21). The results of TRTS are shown in Fig. 4a, which range from 20 to 80 cm^2 ^V^−1^ s^−1^ and are compared to the measured network mobilities in Fig. 4b. We find that in all cases, *μ*_Net_ <*µ*_NS_, confirming the devices are junction limited. The tungsten-based samples, WSe_2_ and WS_2_ have higher *µ*_NS_ than the molybdenum-based samples, MoS_2_ and MoSe_2_, suggesting lower defect contents (Supplementary Note 21). The higher *µ*_NS_ for the selenide-based samples than the sulphur-based samples could be due to the former being less defective. Nb-doping appears to reduce the mobility for both tungsten and molybdenum-based samples, probably due to defect or impurity scattering^54^.

We have recently demonstrated that A.C. impedance spectroscopy allows *R*_J_ and *R*_NS_, to be measured simultaneously^22^, although we only reported data for MoS_2_. In this method, the measured network impedance is converted into the impedance of the average nanosheet-junction pair, *Z*_NS-J_ (Supplementary Note 22). The real *Z*_NS-J_ spectrum is then fitted with an equivalent circuit model to yield values of *R*_NS_, *R*_J_. Such spectra are shown in Fig. 4c for networks of MoS_2_, WS_2_, MoSe_2_, and MoSe_2_:Nb with fits shown as solid lines (See “Methods” and Supplementary Note 22). The resultant *R*_J_ and *R*_NS_ data are shown in Fig. 4d. The *R*_J_ values for our EE networks are >1000 times lower than reported values for LPE nanosheet networks (*R*_*J*_ = 6–24 GΩ)^22^, emphasising the junction quality in EE networks. However, in all cases, we find *R*_J_/*R*_NS_ > 1, indicating that the networks are junction-limited, and consistent with our conclusions from THz spectroscopy. As mentioned above, *µ*_Net_ and *R*_J_ are linked in nanonetworks via ${R}_{J}/{R}_{{\mathrm{NS}}}\approx {\mu }_{{\mathrm{NS}}}/{\mu }_{{\mathrm{Net}}}-1$^22^. As shown in Fig. 4e, this relationship holds well in our networks further emphasising the junction-limited nature of these systems. However, for MoS_2_, we find *R*_J_/*R*_NS_ = 3.3, consistent with only mild junction limitations. In the future, junction resistances are likely to be further reduced by nanosheet optimisation and process engineering such that we anticipate that *R*_J_ < *R*_NS_ is achievable, leading to *μ*_Net_ ~*µ*_NS_. To help understand the factors involved, we plot *μ*_Net_/*μ*_NS_ versus mean nanosheet AR. With the exception of the MoS_2_ and Mo_0.5_W_0.5_Se_2_ data points, we see a well-defined trend where *μ*_Net_/μ_NS_ increases super-linearly with AR in Fig. 4f. This is probably a manifestation of the fact that increasing AR results in higher *R*_J_ (due to reduced nanosheet thickness) and lower *R*_NS_ (larger nanosheets should lead to larger junction area). Thus, increasing AR is a promising route to increased *R*_NS_/*R*_J_ and so higher values of *μ*_Net_/μ_NS_.

### Functional circuits from solution-processed networks

Only a small minority of 2D materials produced by EE have been studied for circuit applications. Above, we surveyed a range of *n*, *p*, and ambipolar 2D networks using electrochemically-gated transistor measurements because of the simplicity of this technique. However, due to their low ionic mobility, electrochemical transistors are often too slow (> 100 ms response times)^10^ to be used in practical circuits. Therefore, we select our most promising materials, WS_2_ and Mo_0.5_W_0.5_Se_2_, for use in solid-state field-effect transistors (FETs) and circuits using native AlO_x_ as a dielectric (See “Methods”). We use these FETs to make a combination of DACs (Supplementary Note 26, 27), inverters (Supplementary Note 24) and buffer circuits (Supplementary Note 28), demonstrating circuits with complementary functionality.

**Fig. 5:**
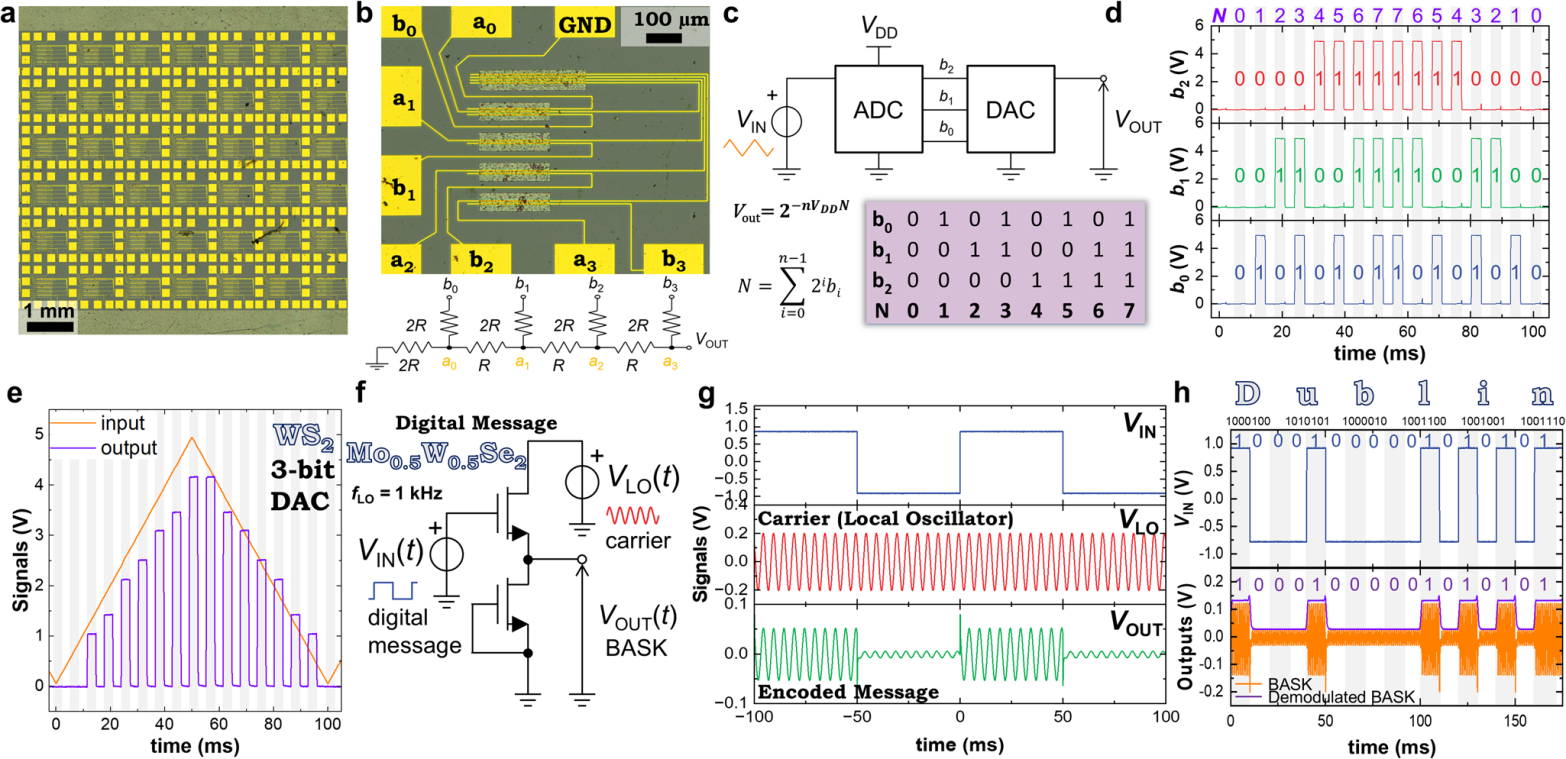
Solution processed DAC and BASK circuits. **a** Bright-field optical microscopy image of the WS_2_ digital-to-analogue converters. **b** Magnified optical image of the solution-processed WS₂ four-bit digital-to-analogue converter (DAC) with the corresponding circuit diagram. The DAC is based on a binary weighted resistor network. The outputs are referenced to ground (GND). The input lines are labelled a_0_, a_1_, a_2_, a_3_ for the resistor connections and b_0_, b_1_, b_2_, b_3_ for the corresponding digital bit inputs, where b_3_ is the most significant bit and b_0_ the least significant bit. **c** Circuit diagram of the analogue-to-digital converter (ADC) connected with three bits to the solution-processed DAC, together with the corresponding logic table. The input analogue voltage (*V*_IN_) is converted by the ADC into a digital signal, which is then supplied to the DAC. The DAC produces a reconstructed analogue output voltage (*V*_OUT_). The digital outputs of the ADC are referenced to the supply voltage (*V*_DD_). **d** Digital signals from bit lines in a 3-bit DAC, showing the contribution of each digital signal to the output voltage. **e** Analogue input to the ADC (orange line) and the corresponding analogue output (purple line) from the 3-bit WS_2_ DAC. **f** Circuit diagram of the binary amplitude shift keying (BASK) circuit used for signal encoding. A high-frequency local oscillator with frequency (*f*_LO_) provides a sinusoidal carrier signal with amplitude denoted as *V*_LO_. The digital input controls the modulation of the carrier, producing an amplitude-encoded output. **g** Digital signal encoding into a high-frequency analogue signal. The digital message input into the top FET gate modulates the high-frequency carrier signal to produce the encoded output. **h** Binary bits required to spell the word Dublin in 7-bit ASCII. *V*_IN_ represents the digital message shown by the blue curve. The output of the BASK circuit, which has the encoded digital message in the local oscillator, is shown by the orange curve, and the decoded signal is shown as the purple line.

The arrays of DAC circuits are shown in Fig. 5a with optical microscopy (Leica DM6M microscope). The process involves using a binary weighted resistor network (*R*-2*R* ladder network) to convert a digital binary input into an analogue output voltage^55^. Figure 5b magnifies the circuits and shows an optical image of the solution-processed WS_2_ 4-bit ladder network and its corresponding circuit diagram. We use the solution-processed DAC to restore an analogue signal, as shown in the circuit diagram of Fig. 5c. We input an analogue triangular waveform, *V*_IN_, into a conventional Si AD7819 analogue-to-digital converter (ADC), which outputs a digital signal of logic ‘1’ or ‘0’. We first make a 3-bit DAC and connect the ADC to the DAC using bit lines (*b*_2_, *b*_1_, and *b*_0_), where each bit represents a power of 2 in binary weighting. In our 3-bit DAC, *b*_2_ represents the most significant bit and *b*_0_ represents the least significant bit. We define *V*_DD_ as the power supply voltage of the ADC. If the value of a bit line is ‘1’, the corresponding ADC output will go to the value of *V*_DD_, and if it is ‘0’ the output will go to ground. Figure 5d shows the digital signals of bits *b*_2_ (red), *b*_1_ (green), and *b*_0_ (blue), and the corresponding binary weighted integer *N*. We achieve these weights by summating the contribution of each digital signal to the output voltage, *V*_OUT_, of the DAC. The contribution of each signal is halved for each node going to the output. For example, in a 3-bit DAC, the contribution of *b*_2_ is halved once, *b*_1_ twice and *b*_0_ three times. In Fig. 5e, we show the *V*_IN_ (orange) to the ADC and the analogue output of the WS_2_ DAC (purple line). A more detailed explanation of the DAC operation can be found in Supplementary Note 26. We can also increase the complexity of the circuit by making a 4-bit WS_2_ DAC that uses an additional binary weighted resistor (*b*_3_) to give 16 discrete *V*_OUT_ values (Supplementary Note 27).

As an additional circuit demonstration, we use a digital signal, similar to the output of the ADC, and encode it into an analogue signal using a binary amplitude shift keying (BASK) circuit, as shown in Fig. 5f. We make a buffer using the manufacturing protocol of the NMOS depletion logic. In this case, the top Mo_0.5_W_0.5_Se_2_ FET (driver) is used as a switch, and the bottom transistor is used as a depletion load. We provide a high-frequency (1 kHz) A.C. sine wave, with an amplitude of 200 mV, to act as a carrier signal denoted as the local oscillator, *V*_LO_. The digital message is shown at the top of Fig. 5g, as the *V*_IN_ to the gate of the top FET. When *V*_IN_ is −1 V, the top Mo_0.5_W_0.5_Se_2_ FET is off and *V*_OUT_ is low and near zero volts. The top FET cannot be completely turned off, so there is a small amplitude signal (7.2 mV) at *V*_OUT_ according to voltage divider circuit analysis (*V*_OUT_ = *V*_LO_ × *R*_L_/(*R*_CH_ + *R*_L_)), where *R*_CH_ and *R*_L_ are the channel resistances of the top and bottom FET, respectively. When *V*_IN_ = 0.87 V, the top Mo_0.5_W_0.5_Se_2_ FET is on, and the carrier signal from the *V*_LO_ is passed to the output. Thus, we can encode the information from the digital signal into the amplitude of the high-frequency analogue signal of *V*_LO._ We undertake a practical demonstration by demodulating a modulated encoded binary message from the high-frequency signal using an AM demodulator (Supplementary Note 28). The message ‘Politecnico di Milano and University of Dublin’ was encoded using 7-bit ASCII, resulting in 322 bits (data rate of 100 bits per second). Figure 5h shows the binary input to *V*_IN_ required to encode the message ‘Dublin’. The BASK circuit converts the binary sequence into an amplitude-modulated signal, shown as the orange curve in Fig. 5h. The purple curve shows a 175 ms snippet of the decoded signal to reveal the letter ‘U’ (Supplementary Note 28). These circuits help to understand the practical aspects of digital communication and signal processing in solution-processed 2D networks.

## Discussion

We measure the electrical properties of low-bandgap 2D materials for solution-processed electronic applications. We determine that a high in-plane to out-of-plane stiffness ratio >1.7 and *E*_b_ < 40 meV Å^−2^ is essential to enable crystal expansion. Electrical characterisation of solution-deposited networks confirmed that a subset of these materials could achieve high mobility and *I*_on_/*I*_off_ for both p-type and n-type 2D nanosheets. Measuring the nanosheet mobility for a range of materials (20–80 cm² V^−^¹ s^−^¹), clarified the upper limit for network mobility. We decoupled the junction and nanosheet resistance for materials beyond MoS_2_ and find the electrical performance is constrained by junction resistance (*R*_J_/*R*_NS_ = 3.3–23), which advances the understanding of performance limitations in 2D networks. Reducing these values below one will yield network mobilities approaching the intrinsic nanosheet limit. After a rigorous down selection of material electrical properties, we utilised WS_2_ for the development of solution-processed DACs, while Mo_0.5_W_0.5_Se_2_ was utilised in a BASK circuit. A 7-bit ASCII message modulated with a solution-processed circuit and then decoded, which could be particularly useful for future solution-processed communication systems.

## Methods

### Electrochemical exfoliation

An electrochemical cell with two electrodes is utilised to intercalate the crystals. A small crystal piece measuring 0.1 × 1 × 1 mm serves as the cathode, while a platinum foil (Alfa Aesar) is employed as the anode. The electrodes are secured using copper crocodile clips. The electrolyte solution is prepared by dissolving tetrapropylammonium (TPA) bromide (5 mg/mL, Sigma–Aldrich) in approximately 50 mL of propylene carbonate. An 8 V voltage is applied across the electrodes for 30 min to intercalate the 2D crystal with TPA+ cations. Following this process, the 2D crystal expands to more than twice its original volume, confirming successful intercalation.

### Ink formulation

The expanded 2D crystal is subjected to bath sonication (Fisherbrand 112xx series) in a solution of 1 mg/mL poly(vinylpyrrolidone) (PVP, molecular weight ∼40,000) dissolved in dimethylformamide (DMF) for 5 min. This is followed by centrifugation (Hettich Mikro 220, 1195-A, radius 87 mm) at 500 rpm (24 g) for 20 min to remove unexfoliated crystals. To size-select the dispersion, the supernatant (top 90%) is centrifuged at 1000 rpm (97 *g*) for 1 h, and the sediment is collected. To remove the PVP, the sediment obtained at 97 *g* is diluted with 2 mL of DMF and centrifuged at 10,000 rpm (9744 *g*) for 1 h. This process is repeated twice, with the sediment being collected each time. A third washing step (to remove the DMF) involves diluting the sediment in 0.5 mL of IPA and centrifuging at 10,000 rpm (9744 *g*), after which the sediment is collected. The final sediment is redispersed in approximately 0.5 mL of IPA, which is used in the study for each 2D crystal.

### Raman spectroscopy

Inks of each material are drop-cast onto Si/SiO_2_ substrate and annealed at 120 °C. A Renishaw Raman spectrometer at 532 nm with a 100× objective is used to acquire spectra. An incident power of ∼1 mW was used to minimise possible thermal damage.

### Atomic force microscopy

AFM measurements, including thickness and lateral nanosheet size analysis, are performed using a Bruker Multimode eight microscope. The inks are diluted with IPA at a 1:100 ratio and then drop-cast onto silicon/silicon dioxide (Si/SiO_2_) substrates. Post-dilution, the samples undergo an annealing process at 120 °C for 30 min to evaporate any remaining solvent. For scanning, OLTESPA R3 cantilevers are employed in the ScanAsyst mode to systematically scan the samples. Approximately 25–50 nanosheets per sample are analysed to gather statistical data. The lateral size of the nanosheets is determined by taking the square root of the product of the nanosheet’s length and width.

### Scanning electron microscopy

SEM imaging was conducted using a Carl Zeiss Ultra SEM. A secondary electron detector was used to obtain the images at a 3 kV accelerating voltage, 5 mm working distance and 30 μm aperture.

### Optical absorption spectroscopy

The spectra of nanosheet dispersions were collected by a Cary 1050 spectrometer from 900 nm to 200 nm with a 1–2 nm step. The dispersion was placed in a 10 mm optical length cuvette. The absorption spectra were collected in an integrating sphere. The collected extinction and absorption spectra of nanosheet dispersions were subtracted from their corresponding solvent spectra to yield nanosheet-only spectra. The scattering spectra were obtained by using extinction spectra and subtracting absorption spectra.

### Langmuir–Schaefer deposition

The LS setup involves a Teflon stand where a PET substrate (2 × 2 cm, Novele, Novacentrix) (unless stated otherwise) is placed on top. The stand is placed in a 100 mL beaker of deionized water. About 20 mL of distilled hexane is drop-cast onto the surface of the deionized water to create a water/hexane interface, under which the PET on the Teflon stand is submerged. Ink was drop-cast (∼120 μL) onto the surface of the hexane until no gaps in the interface could be seen. A needle attached to a vacuum pump is used to extract the deionized water from the bottom of the LS set-up, which extrudes the PET though the 2D crystal layer at a reproducible and consistent rate^6^. The process is repeated on separate PET substrates for every material to build the second layer of the network, except for MoS_2_, which only uses one deposition layer. For GaTe, InTe, SnSe, and SnS networks, Si/SiO_2_ substrates (Fraunhofer Gen 5 OFET chips) with pre-patterned gold electrodes were used. The 2D nanosheet networks are annealed at 120 °C for 1 h on a hot plate in an N_2_ glovebox (Jacomex GP campus) for each layer of deposition to remove residual solvent and improve the adhesion of the 2D nanosheets to the substrate.

### Evaporation

Gold electrodes (∼100 nm thick) are deposited by evaporation (FC-2000 Temescal Evaporator) through a stainless steel mask (50 μm thick), which is laser cut (Laser Micromachining Ltd). The gold defines the channel dimensions of *W*_CH_ = 11000 μm and *L*_CH_ = 50 μm. The Au evaporation also defines our gate electrode, which is placed ∼1 mm from the source and drain electrodes and is ∼1.5 × 4 mm in size. These electrodes are used for the electrical characterisation presented in Fig. 3 for all materials except GaTe, InTe, SnSe, and SnS, which used pre-patterned gold electrodes from Fraunhofer. The Fraunhofer chips consisted of an array of four transistors with *L*_CH_ = 2.5 μm, *W*_CH_ = 10 mm and 230 nm SiO_2_ thickness, with a gate of n-doped silicon (*n* ∼ 10^17 ^cm^−3^). We used the Fraunhofer chips for the electrical characterisation of these materials since the nanosheets were small <500 nm, and therefore it was difficult to create a percolative network over a large *L*_CH_ = 50 μm made by the shadow mask. *L*_CH_ < 50 μm was not possible to achieve with the shadow masks without shorting the source and drain electrodes.

### Stretchable conductor

TaS_2_ devices were tested by mounting samples in a Zwick Z0.5 Proline tensile tester with a 100 N load cell. The device was strained using a triangular sawtooth pattern at 2% strain, at a rate of 2%/s. Conductive samples were contacted using conductive silver paint and silver wires. Two-probe electronic measurements were taken using a Keithley KE2601 source metre.

### Impedance spectroscopy

Fused silica substrates were purchased from MicroChemicals and used as the substrate for the impedance spectroscopy measurements. We use evaporated (FC-2000 Temescal Evaporator) Ti/Au (5 nm/95 nm) on the nanosheet network with a shadow mask with *L*_CH_ = 25–200 µm, *W*_CH_ = 19.4 mm. With a maximum frequency of 30 MHz, the Keysight E4990E analyser was used to obtain the impedance spectra. In order to minimise inductive artefacts at high frequencies, a test fixture (16047E) was utilised to connect the samples to the analyzer. This fixture allowed for a wire distance as short as 5 cm. Sensepeek SP10, a spring-loaded probe attachment, was utilised to link the analyzer to the substrates’ contact pads. The spectra were obtained at a precision speed of three, with an amplitude of 500 mV. The network impedance, *Z*_Net_, is converted into the impedance of the average nanosheet-junction pair, *Z*_NS*-J*_ using the equation^22^,

1 $${Z}_{{\mathrm{NS}}-J}={\rho }_{{\mathrm{Net}}}^{\ast }\frac{(1-{P}_{{\mathrm{Net}}})}{2{t}_{{\mathrm{NS}}}}{\left[1+\frac{2}{{n}_{{\mathrm{NS}}}{t}_{{\mathrm{NS}}}{l}_{{\mathrm{NS}}}^{2}}\right]}^{-1}$$

ρ^*^_Net_, is the complex resistivity of the nanoheet network, ρ^*^_Ne*t*_ = *Z*_Net_*A/L*_CH_, and *A* and *L*_CH_ are the network thickness and channel length. *P*_Net_ is the meso-porosity of the network previously found to be about once *P*_Net_ ∼0.02, <*t >*, <*L* > and *n* are known. The *Z*_NS*-J*_ spectrum is then fitted with an equivalent circuit model of a Randles circuit to yield values of *R*_NS_, *R*_*J*_^22^.

### THz spectroscopy

A titanium-doped regenerative amplifier (Libra), the foundation of our THz spectroscopy apparatus, generates 60 fs laser pulses with an 800 nm central wavelength. Optical photoexcitation of the sample, THz creation, and THz detection are the three separate uses for the amplifier’s output. The first part of the beam is optically transformed by frequency doubling in a BBO crystal to a pump wavelength of 400 nm (photon energy 3.1 eV). Optical rectification at 800 nm is utilised in the second half to generate a THz waveform in a nonlinear ZnTe crystal with a duration of around 1 ps. The third component (at 800 nm) uses electro-optic sampling to detect the THz waveform after it has passed through the sample in a different ZnTe crystal. Mechanical delay stages regulate the time delays between the THz generation and detection pulse (*t*) as well as between the photoexcitation pump pulse and the THz detection pulse (*τ*). All of the experiments were carried out in a closed box with an N_2_ environment at room temperature.

### Transistor measurements

We used a gold side gate (∼1.5 mm × 4 mm) ∼1 mm for the electrodes and 1-ethyl-3-methylimidazolium bis(trifluoromethylsulfonyl)imide (EMIM TFSI) to switch the channel^42^. We used an FC-2000 Temescal Evaporator to evaporate Ti/Au (5 nm/95 nm) electrodes through a shadow mask with dimensions of *L*_CH_ = 50 µm and *W*_CH_ = 11 mm onto our LS deposited networks. We control ion injection into our semiconducting channel using the ionic liquid 1-ethyl-3-methylimidazolium bis(trifluoromethylsulfonyl)imide (EMIM-TFSI, Sigma–Aldrich). The ionic liquid is prepared by vacuum heating it at 100 °C for 6 h in order to remove any absorbed water. The transistor is then carefully pipetted with a small amount of EMIM to make sure the gate and channel are sufficiently coated. The devices are vacuum-sealed for a full 12-h period overnight in a Janis probe station in order to remove any residual water. The devices are then brought back to atmospheric pressure in order to be ready for measurements. We use a Keithley 2612A dual-channel source measurement instrument for electrical characterisation. Using a scan rate of 50 mV s^−1^, the transfer characteristics are carried out within a gate voltage window of −3–3 V. Additionally, throughout the measurements, *V*_DS_ is set to 1 V for every device.

### Solid-state electrostatic devices and electron-beam (e-beam) lithography

A fixed-beam moving-stage (FBMS) mode of a Raith eLINE system is operated at 30 keV to fabricate contacts for DACs, NMOS, and BASK circuits. The FBMS mode allowed the patterning of very wide (650 μm) electrodes without stitching errors. Source and drain contacts for FETs used in NMOS and BASK circuits are 350 µm wide and are patterned in a 500 µm write field using a standard exposure mode. The electrodes were made of Ti/Au deposited in the e-beam evaporator at a base pressure of ≈1.2 × 10^−6^ mbar. A thin native AlO_x_ layer, approximately 4 nm thick, formed on the surface of Al after the samples were exposed to air was used as the gate insulator.

### Solution-processed DACs

Highly doped Si/SiO_2_ wafers (90 nm SiO_2_) are utilised as substrates to fabricate the circuits. We extrude the wafers through a network of WS_2_ nanosheets twice using the LS process. The WS_2_ channels are defined by e-beam lithography and reactive ion etching (RIE) in SF_6_ for 1 min at a power of 20 W. Next, nine electrodes were patterned to make an *R*-2*R* ladder network by e-beam lithography (*W*_CH_ = 200 and 300 µm for 2*R* and *W*_CH_ = 300, 400, 500, and 600 µm for *R*, with *L*_CH_ = 4 µm in all cases), followed by deposition of Ti/Au (3/80 nm) in an e-beam evaporator at a base pressure of ≈1.2 × 10^−6^ mbar. The ADC used also has another input voltage (*V*_REF_), which defines the maximum expected voltage at the analogue input (*V*_IN_MAX_). For simplicity, we used *V*_REF_ = *V*_DD_.

### Solution-processed NMOS and BASK circuit

First, we fabricate gate contacts by e-beam lithography and e-beam evaporation of Al (40 nm) onto the Si/SiO_2_ wafers (90 nm SiO_2_) and expose the samples to air to form a native AlO_*x*_ layer approximately 4 nm thick^56^, which serves as the gate oxide. The oxide capacitance per surface area is *C*_ox_ ~1.4 µF/cm^2^, similar to previous work^24^. Next, we fabricate the source and drain contacts (*L*_CH_ = 1, 2, 3, 5, and 10 µm) of the FETs by e-beam lithography and e-beam evaporation of Ti/Au (3/37 nm). For the driver FET (*V*_GS_ = *V*_IN_): *W*_CH_ = 300 µm and *L*_CH_ = 5 µm and for the load FET (*V*_GS_ = 0 V): *W*_CH_ = 300 µm, *L*_CH_ = 10 µm. We extrude the wafers through a network of Mo_0.5_W_0.5_Se_2_ nanosheets twice using the LS process. The Mo_0.5_W_0.5_Se_2_ channels (*W*_CH_ = 200 and 300 µm) are defined by e-beam lithography and RIE in SF_6_ for 1 min at a power of 20 W.

### Crystals

We prepare TMDs and TMTs by chemical vapour transport in quartz ampoules, sealed under high vacuum, with a series of controlled heating and cooling stages involving two-zone furnaces to create thermal gradients for crystal growth. PtSe_2_ and PdSe_2_ are prepared by directly reacting elements in a stoichiometric ratio at high temperature (>800 °C) in a quartz ampoule. The BP is made by transporting red phosphorus in the presence of a mineraliser (SnI_4_/Sn) in a quartz ampoule, sealed under a high vacuum and heated gradually in a muffle furnace. The TMMs are prepared by direct reaction of elements in a stoichiometric ratio in quartz ampoules, sealed under a high vacuum, heated above the melting point in a muffle furnace, and cooled slowly. The MoS_2_ is of natural origin, and Bi_2_O_2_Se, thallium selenide (TlSe), tellurium, tin selenide (SnSe), tin sulphide (SnS) and germanium were bought commercially. Each crystal has a unique manufacturing protocol presented in further detail below. The crystals we will grow include, indium telluride (InTe), gallium telluride (GaTe), germanium sulphide (GeS), germanium selinide (GeSe), tungsten diselenide (WSe_2_), molybdenum disulphide (MoS_2_), molybdenum diselenide (MoSe_2_), tungsten disulphide (WS_2_), molybdenum ditelluride (MoTe_2_), molybdenum tungsten diselenide (Mo_0.5_W_0.5_Se_2_), platnium diselenide (PtSe_2_), palladium diselenide (PdSe_2_), tantalum disulphide (TaS_2_), tin diselenide (SnSe_2_), cobalt phosphorous trisulfide (CoPS_3_), iron phosphorous trisulfide (FePS_3_), nickel phosphorous trisulfide (NiPS_3_) along with hafnium triselenide (HfSe_3_).

### Chemicals

Sulphur (99.9999%, 2–6 mm), selenium (99.9999%, 2–4 mm), tellurium (99.9999%, 2–6 mm), phosphorus (99.9999%, 2–6 mm), Ge (99.9999%, 1–6 mm), Sn (1–4 mm, 99.9999%), Ga (99.9999%, granules 1–6 mm), In (99.9999%, 1–3 mm) were obtained from Wuhan Xinrong New Materials Co., China. Tungsten (99.999%, −100 mesh) was obtained from China Rhenium Co., China. Niobium (99.9%, −100 mesh), tantalum (99.9%, −100 mesh) and hafnium (99.9%, −100 mesh) were obtained from Beijing Metallurgy and Materials Technology Co., China. nickel (99.99%, −100 mesh), iron (99.9%, −100 mesh), iodine (99.9%, granules), SeCl_4_ (99.5%), WCl_6_ (99.9%), TeCl_4_ (99.9%), cobalt (99.9%, −100 mesh) were obtained from Strem, USA, platinum (99.99%, −100 mesh) was obtained from SurePure, USA. Palladium (99.95%, −100 mesh) was obtained from Safina, Czech Republic.

### MoS_2_

MoS_2_ of natural origin was collected in Krupka, Czech Republic, by Z.S.

### Commercial crystals

Bi_2_O_2_Se (2D semiconductors), SnS (2D semiconductors), SnSe (2D semiconductors), TlSe (2D semiconductors), tellurium (Novaelements) and germanium (Novaelements) were bought commercially.

### Black phosphorus

BP was made by transport of red phosphorus in presence of mineralizer using standard procedure with SnI_4_/Sn. Red phosphorus (2 g) and Sn (30 mg)/SnI_4_ (60 mg) (mineralising agent) were placed in a quartz glass ampule and subsequently sealed using an oxygen/hydrogen torch under high vacuum (under 1 × 10^−3 ^Pa using oil diffusion pump and LN_2_ trap). The ampule was placed horizontally in a muffle furnace and heated to 600 °C in 8 h. After 6 h on temperature the ampoule was cooled to 400 °C over a period of 50 h, subsequently on 200 °C in 25 h and finally freely cooled at room temperature for 2 h. The obtained BP crystals with a metallic sheen were washed with CS2 to remove the byproduct of white phosphorus and stored in argon glovebox.

### GeS

GeS was made by direct reaction of elements (25 g) in stochiometric ratio in quartz ampoule (100 × 25 mm) sealed under high vacuum (under 1 × 10^−3 ^Pa using oil diffusion pump and LN_2_ trap). The ampoule were placed horizontally in muffle furnace and heated above melting point. The ampoule was heated using 0.5 °C min^−1^ to 700 °C and after 6 h cooled to room temperature at 0.1 °C min^−1^. Ampoule was open in glovebox and sample was kept under argon till further use.

### GeSe

GeSe was made by direct reaction of elements (25 g) in stochiometric ratio in a quartz ampoule (100 × 25 mm) sealed under high vacuum (under 1 × 10^−3^ Pa using oil diffusion pump and LN_2_ trap). The ampoule were placed horizontally in muffle furnace and heated above melting point. The ampoule was heated using 1 °C min^−1^ to 700 °C and after 6 h cooled on room temperature sing 0.1 °C min^−1^. Ampoule was open in glovebox and sample was kept under argon till further use.

### SnSe_2_

SnSe_2_ was made by direct reaction of elements (25 g) in stochiometric ratio in quartz ampoule (100 × 25 mm) sealed under high vacuum (under 1 × 10^−3 ^Pa using an oil diffusion pump and LN_2_ trap). The ampoule were placed horizontally in muffle furnace and heated above melting point. The ampoule was heated using 1 °C min^−1^ on 700 °C and after 6 h cooled on room temperature at 0.1 °C min^−1^. Ampoule was open in glovebox and sample was kept under argon till further use.

### GaTe

GaTe was made by direct reaction of elements (25 g) in stochiometric ratio in quartz ampoule (100 × 25 mm) sealed under high vacuum (under 1 × 10^−3 ^Pa using an oil diffusion pump and LN_2_ trap). The ampoules were placed horizontally in muffle furnace and heated above melting point. The ampoule was heated using 1 °C min^−1^ to 830 °C and after 6 h cooled on room temperature sing 0.1 °C min^−1^. Ampoule was open in glovebox and sample was kept under argon till further use.

### InTe

InTe was made by direct reaction of elements (25 g) in stochiometric ratio in quartz ampoule (100 × 25 mm) sealed under high vacuum (under 1 × 10^−3 ^Pa using oil diffusion pump and LN_2_ trap). The ampoule were placed horizontally in muffle furnace and heated above melting point. The ampoule was heated using 1 °C min^−1^ on 710 °C and after 6 h cooled on room temperature sing 0.1 °C min^−1^. Ampoule was open in glovebox and sample was kept under argon till further use.

### InSe

InSe was made by direct reaction of elements (25 g) in ratio 52 at% In and 48 at% Se in quartz ampoule (100 × 25 mm) sealed under high vacuum (under 1 × 10^−3 ^Pa using oil diffusion pump and LN_2_ trap). The ampoule were placed horizontally in muffle furnace and heated above melting point. The ampoule was heated using 1 °C min^−1^ on 710 °C and after 6 h cooled on room temperature sing 0.1 °C min^−1^. Ampoule was open in glovebox and sample was kept under argon till further use.

### PtSe_2_

PtSe_2_ was made by direct reaction from elements in quartz glass ampoule of 100 × 25 mm dimension. For the synthesis of PtSe_2_ was in ampoule placed 3 g of Pt and Se with 2 at% excess of selenium. The ampoule was placed in muffle furnace and heated on 800 °C for 25 h and subsequently was heated on 1280 °C (1 °C min^−1^) and after 1 h was cooled over a period of 100 min on 1200 °C and subsequently on room temperature using cooling rate 1 °C min^−1^.

### PdSe_2_

For the synthesis of PdSe_2_ the elements corresponding to 3 g of PdSe_2_ were placed in stochiometric ratio in ampoule and melt-sealed under high vacuum (under 1 × 10^−3 ^Pa using oil diffusion pump and LN_2_ trap). The ampoule was heated at 820 °C (1 °C min^−1^) and after 5 h cooled on room temperature using cooling rate 0.1 °C min^−1^.

### MoSe_2_, WSe_2_, Mo_0.5_W_0.5_Se_2_

The crystals were made by chemical vapour transport in a quartz ampoule. The elements corresponding to the 50 g of compound were placed in a quartz ampoule (50 × 250 mm) together with 2 at.% excess of selenium and 0.5 g of SeCl_4_ and melt sealed under high vacuum (under 1 × 10^−3 ^Pa using oil diffusion pump and LN_2_ trap). The ampoule was placed in muffle furnace and heated on 500 °C for 25 h, on 600 °C for 50 h and finally at 800 °C for 50 h. Subsequently the ampoule were placed in two two-zone furnace and first the growth zone was heated on 1000 °C and source zone on 800 °C, after 2 days the thermal gradient was reversed and the source zone was heated on 950 °C while the growth zone was kept at 850 °C for 14 days. The ampoule was open under argon atmosphere in glovebox and keep under argon atmosphere till further use.

### Nb doped MoSe_2_ and WSe_2_

The Nb doped materials were made by mixing the elements in ration W_0.97_Nb_0.03_Se_2_ (Mo_0.97_Nb_0.03_Se_2_) with total mass of 50 g and 2 at.% excess of Se and 0.5 g of SeCl_4_ were placed in ampoule and melt sealed under high vacuum (under 1 × 10^−3 ^Pa using oil diffusion pump and LN_2_ trap). The ampoule was placed in muffle furnace and heated on 500 °C for 25 h, on 600 °C for 50 h and finally on 800 °C for 50 h. Subsequently the ampoule were placed in two zone furnace and first the growth zone was heated on 1000 °C and source zone on 800 °C, after 2 days the thermal gradient was reversed and the source zone was heated on 950 °C while the growth zone was kept at 850 °C for 14 days. The ampoule was open under argon atmosphere in glovebox and keep under argon atmosphere till further use.

### TaS_2_

The crystals were made by chemical vapour transport in quartz ampoule. The elements corresponding to the 50 g of compound were placed in quartz ampoule (50 × 250 mm) together with 0.7 g of I_2_ and melt sealed under high vacuum (under 1 × 10^−3 ^Pa using oil diffusion pump and LN_2_ trap). The ampoule was placed in muffle furnace and heated on 500 °C for 25 h, on 600 °C for 50 h and finally on 800 °C for 50 h. Subsequently the ampoule were placed in two zone furnace and first the growth zone was heated on 1000 °C and source zone on 800 °C, after 2 days the thermal gradient was reversed and the source zone was heated on 950 °C while the growth zone was kept at 850 °C for 14 days. The ampoule was open under argon atmosphere in glovebox and keep under argon atmosphere till further use.

### MoTe_2_

The crystals were made by chemical vapour transport in a quartz ampoule. The elements corresponding to the 50 g of compound were placed in a quartz ampoule (50 × 250 mm) together with 2 at.% excess of tellurium and 0.5 g of TeCl_4_ and melt sealed under high vacuum (under 1 × 10^−3 ^Pa using oil diffusion pump and LN_2_ trap). The ampoule was placed in muffle furnace and heated on 500 °C for 25 h, on 600 °C for 50 h and finally on 800 °C for 50 h. Subsequently, the ampoule were placed in two zone furnace and first the growth zone was heated on 1000 °C and source zone on 800 °C, after 2 days the thermal gradient was reversed and the source zone was heated on 900 °C while the growth zone was kept at 800 °C for 14 days. The ampoule was open under argon atmosphere in glovebox and keep under argon atmosphere till further use.

### WS_2_

The crystals were made by chemical vapour transport in quartz ampoule. The elements corresponding to the 50 g of compound were placed in quartz ampoule (50 × 250 mm) together with 2 at.% excess of sulphur and 0.5 g of WCl_6_ and melt sealed under high vacuum (under 1 × 10^−3 ^Pa using oil diffusion pump and LN_2_ trap). The ampoule was placed in muffle furnace and heated on 500 °C for 25 h, on 600 °C for 50 h and finally on 800 °C for 50 h. Subsequently, the ampoule were placed in two zone furnace and first the growth zone was heated on 1000 °C and source zone on 800 °C, after 2 days the thermal gradient was reversed and the source zone was heated on 1000 °C while the growth zone was kept at 900 °C for 14 days. The ampoule was open under argon atmosphere in glovebox and keep under argon atmosphere till further use.

### HfSe_3_

The crystals were made by chemical vapour transport in a quartz ampoule. The elements corresponding to the 50 g of compound were placed in a quartz ampoule (50 × 250 mm) together with 2 at.% excess of selenium and 0.5 g of HfCl4 and melt sealed under high vacuum (under 1 × 10^−3 ^Pa using oil diffusion pump and LN_2_ trap). The ampoule was placed in muffle furnace and heated on 500 °C for 25 h, on 600 °C for 50 h and finally on 800 °C for 50 h. Subsequently, the ampoule were placed in two zone furnace and first the growth zone was heated on 1000 °C and source zone on 800 °C, after 2 days the thermal gradient was reversed and the source zone was heated on 900 °C while the growth zone was kept at 800 °C for 14 days. The ampoule was open under argon atmosphere in glovebox and keep under argon atmosphere till further use.

### NiPS_3_ and FePS_3_

The crystals were made by chemical vapour transport in quartz ampoule. The elements corresponding to the 30 g of compound were placed in quartz ampoule (50 × 250 mm) together with 1 at.% excess of sulphur and phosphorus and 0.5 g of I2 and melt sealed under high vacuum (under 1 × 10^−3 ^Pa using oil diffusion pump and LN_2_ trap). The ampoule was placed in muffle furnace and heated on 500 °C for 25 h, on 600 °C for 50 h and finally on 700 °C for 50 h (heating rate was 0.2 °C min^−1^). Subsequently, the ampoule were placed in two zone furnace and first the growth zone was heated on 750 °C and source zone on 600 °C, after 2 days the thermal gradient was reversed and the source zone was heated on 750 °C while the growth zone was kept at 650 °C for 14 days. The ampoule was open under argon atmosphere in glovebox and keep under argon atmosphere till further use.

### CoPS_3_

The crystals were made by chemical vapour transport in quartz ampoule. The elements corresponding to the 15 g of compound were placed in quartz ampoule (35 × 150 mm) together with 1 at.% excess of sulphur and phosphorus and 0.5 g of I_2_ and melt sealed under high vacuum (under 1 × 10^−3 ^Pa using oil diffusion pump and LN_2_ trap). The ampoule was placed in muffle furnace and heated on 560 °C for 30 days (heating rate was 0.1 °C min^−1^). The ampoule was open under argon atmosphere in glovebox and keep under argon atmosphere till further use.

## Supplementary information

Supplementary Information

Transparent Peer Review file

## Data Availability

Relevant data supporting the key findings of this study are available within the article and the Supplementary Information file. All raw data generated during the current study are available from the corresponding authors upon request.

## References

[1] Carey, T. et al. Knot architecture for biocompatible and semiconducting 2D electronic fiber transistors. *Small Methods* **8**, e2301654 (2024). 38602193 10.1002/smtd.202301654

[2] Torrisi, F. & Carey, T. Graphene, related two-dimensional crystals and hybrid systems for printed and wearable electronics. *Nano Today* **23**, 73–96 (2018).

[3] Coleman, J. N. et al. Two-dimensional nanosheets produced by liquid exfoliation of layered materials. *Science* **331**, 568–571 (2011). 21292974 10.1126/science.1194975

[4] Lin, Z. et al. Solution-processable 2D semiconductors for high-performance large-area electronics. *Nature* **562**, 254–258 (2018). 30283139 10.1038/s41586-018-0574-4

[5] Nicolosi, V., Chhowalla, M., Kanatzidis, M. G., Strano, M. S. & Coleman, J. N. Liquid exfoliation of layered materials. *Science* **340**, 1226419–1226419 (2013).

[6] Carey, T. et al. High-mobility flexible transistors with low-temperature solution-processed tungsten dichalcogenides. *ACS Nano* **17**, 2912–2922 (2023). 36720070 10.1021/acsnano.2c11319 PMC9933598

[7] Radisavljevic, B., Radenovic, A., Brivio, J., Giacometti, V. & Kis, A. Single-layer MoS2 transistors. *Nat. Nanotechnol.* **6**, 147–150 (2011). 21278752 10.1038/nnano.2010.279

[8] Backes, C. et al. Equipartition of energy defines the size-thickness relationship in liquid-exfoliated nanosheets. *ACS Nano* **13**, 7050–7061 (2019). 31199123 10.1021/acsnano.9b02234

[9] Hernandez, Y. et al. High-yield production of graphene by liquid-phase exfoliation of graphite. *Nat. Nanotechnol.* **3**, 563–568 (2008). 18772919 10.1038/nnano.2008.215

[10] Kelly, A. G. et al. All-printed thin-film transistors from networks of liquid-exfoliated nanosheets. *Science* **356**, 69–73 (2017). 28386010 10.1126/science.aal4062

[11] Zhao, M., Casiraghi, C. & Parvez, K. Electrochemical exfoliation of 2D materials beyond graphene. *Chem. Soc. Rev.* **53**, 3036–3064 (2024). 38362717 10.1039/d3cs00815k

[12] Wang, S. et al. A library of 2D electronic material inks synthesized by liquid-metal-assisted intercalation of crystal powders. *Nat. Commun.* **15**, 6388 (2024). 39079965 10.1038/s41467-024-50697-z PMC11289403

[13] Hao, Q. et al. Surface-modified ultrathin inse nanosheets with enhanced stability and photoluminescence for high-performance optoelectronics. *ACS Nano* **14**, 11373–11382 (2020). 32809802 10.1021/acsnano.0c03556

[14] Yu, W. et al. High-yield exfoliation of monolayer 1 T’-MoTe(2) as saturable absorber for ultrafast photonics. *ACS Nano* **15**, 18448–18457 (2021). 34714041 10.1021/acsnano.1c08093

[15] Li, J. et al. Printable two-dimensional superconducting monolayers. *Nat. Mater.* **20**, 181–187 (2021). 33106649 10.1038/s41563-020-00831-1

[16] Piatti, E. et al. Charge transport mechanisms in inkjet-printed thin-film transistors based on two-dimensional materials. *Nat. Electron.* **4**, 893–905 (2021).

[17] Jeon, Y. et al. Electrochemically exfoliated phosphorene nanosheet thin films for wafer-scale near-infrared phototransistor array. *npj 2D Mater. Appl.* **6**, 82 (2022).

[18] Kim, J. et al. All-solution-processed van der Waals heterostructures for wafer-scale electronics. *Adv. Mater.* **34**, e2106110 (2022). 34933395 10.1002/adma.202106110

[19] Kelly, A. G., O’Suilleabhain, D., Gabbett, C. & Coleman, J. N. The electrical conductivity of solution-processed nanosheet networks. *Nat. Rev. Mater.* **7**, 217–234 (2021).

[20] Li, X. et al. High-yield electrochemical production of large-sized and thinly layered NiPS(3) flakes for overall water splitting. *Small* **15**, e1902427 (2019). 31172668 10.1002/smll.201902427

[21] Yang, S. et al. A delamination strategy for thinly layered defect-free high-mobility black phosphorus flakes. *Angew. Chem. Int Ed. Engl.* **57**, 4677–4681 (2018). 29474753 10.1002/anie.201801265

[22] Gabbett, C. et al. Understanding how junction resistances impact the conduction mechanism in nano-networks. *Nat. Commun.* **15**, 4517 (2024). 38806479 10.1038/s41467-024-48614-5 PMC11133347

[23] Carey, T. et al. Fully inkjet-printed two-dimensional material field-effect heterojunctions for wearable and textile electronics. *Nat. Commun.* **8**, 1202 (2017). 29089495 10.1038/s41467-017-01210-2 PMC5663939

[24] Carey, T. et al. Inkjet printed circuits with 2D semiconductor inks for high-performance electronics. *Adv. Electron. Mater.* **7**, 2100112 (2021).

[25] Ricciardulli, A. G., Wang, Y., Yang, S. & Samori, P. Two-dimensional violet phosphorus: A p-Type semiconductor for (Opto)electronics. *J. Am. Chem. Soc.* **144**, 3660–3666 (2022). 35179356 10.1021/jacs.1c12931

[26] Tang, B. et al. Solution-processable 2D materials for monolithic 3D memory-sensing-computing platforms: opportunities and challenges. *npj 2D Mater. Appl.* **8**, 74 (2024).

[27] Tang, B. et al. Wafer-scale solution-processed 2D material analog resistive memory array for memory-based computing. *Nat. Commun.* **13**, 3037 (2022). 35650181 10.1038/s41467-022-30519-w PMC9160094

[28] Sivan, M. et al. All WSe(2) 1T1R resistive RAM cell for future monolithic 3D embedded memory integration. *Nat. Commun.* **10**, 5201 (2019). 31729375 10.1038/s41467-019-13176-4 PMC6858359

[29] Taylor, H. R. in *Data Acquisition for Sensor Systems* (ed. H. Rosemary Taylor) Ch. 8, 141–162 (Springer US, 1997).

[30] Pandey, S. K. et al. Controlled p-type substitutional doping in large-area monolayer WSe(2) crystals grown by chemical vapor deposition. *Nanoscale* **10**, 21374–21385 (2018). 30427027 10.1039/c8nr07070a

[31] Ding, X. et al. Bi2O2Se: a rising star for semiconductor devices. *Matter* **5**, 4274–4314 (2022).

[32] Mounet, N. et al. Two-dimensional materials from high-throughput computational exfoliation of experimentally known compounds. *Nat. Nanotechnol.* **13**, 246–252 (2018). 29410499 10.1038/s41565-017-0035-5

[33] Campi, D., Mounet, N., Gibertini, M., Pizzi, G. & Marzari, N. Expansion of the Materials Cloud 2D Database. *ACS Nano* **17**, 11268–11278 (2023). 37310789 10.1021/acsnano.2c11510 PMC10403156

[34] Yang, R. et al. Synthesis of atomically thin sheets by the intercalation-based exfoliation of layered materials. *Nat. Synth.* **2**, 101–118 (2023).

[35] Backes, C. et al. Edge and confinement effects allow in situ measurement of size and thickness of liquid-exfoliated nanosheets. *Nat. Commun.* **5**, 4576 (2014). 25099520 10.1038/ncomms5576

[36] Hudgins, J. L., Simin, G. S., Santi, E. & Khan, M. A. An assessment of wide bandgap semiconductors for power devices. *IEEE Trans. Power Electron.* **18**, 907–914 (2003).

[37] Kim, H. S., Haule, K. & Vanderbilt, D. Mott metal-insulator transitions in pressurized layered trichalcogenides. *Phys. Rev. Lett.* **123**, 236401 (2019). 31868467 10.1103/PhysRevLett.123.236401

[38] Tao, J. et al. Mechanical and electrical anisotropy of few-layer black phosphorus. *ACS Nano* **9**, 11362–11370 (2015). 26422521 10.1021/acsnano.5b05151

[39] Guo, Z., Gu, H., Fang, M., Ye, L. & Liu, S. Giant in-plane optical and electronic anisotropy of tellurene: a quantitative exploration. *Nanoscale* **14**, 12238–12246 (2022). 35929846 10.1039/d2nr03226k

[40] Fiorillo, A. S., Critello, C. D. & Pullano, S. A. Theory, technology and applications of piezoresistive sensors: a review. *Sens. Actuators A: Phys.* **281**, 156–175 (2018).

[41] Caffrey, E. et al. Using electrical impedance spectroscopy to separately quantify the effect of strain on nanosheet and junction resistance in printed nanosheet networks. *Small* n/a, e2406864 10.1002/smll.202406864 (2024). 10.1002/smll.202406864 PMC11798361 39696978

[42] Rivnay, J. et al. Organic electrochemical transistors. *Nat. Rev. Mater.* **3**, 17086 (2018).

[43] Cho, J. H. et al. High-capacitance ion gel gate dielectrics with faster polarization response times for organic thin film transistors. *Adv. Mater.* **20**, 686–690 (2008).

[44] Higgins, T. M. et al. Electrolyte-gated n-type transistors produced from aqueous inks of WS2 nanosheets. *Adv. Funct. Mater.* **29**, 1804387 (2018).

[45] Ma, H. et al. Controlled synthesis of ultrathin PtSe(2) nanosheets with thickness-tunable electrical and magnetoelectrical properties. *Adv. Sci.* **9**, e2103507 (2022). 10.1002/advs.202103507 PMC8728827 34713628

[46] Shi, W. et al. Superconductivity series in transition metal dichalcogenides by ionic gating. *Sci. Rep.* **5**, 12534 (2015). 26235962 10.1038/srep12534 PMC4522664

[47] Okamoto, T. et al. Robust, high-performance n-type organic semiconductors. *Sci. Adv.* **6**, eaaz0632 (2020). 32494668 10.1126/sciadv.aaz0632 PMC7195148

[48] Ha, M. et al. Printed, sub-3V digital circuits on plastic from aqueous carbon nanotube inks. *ACS Nano* **4**, 4388–4395 (2010). 20583780 10.1021/nn100966s

[49] Nomura, K. et al. Room-temperature fabrication of transparent flexible thin-film transistors using amorphous oxide semiconductors. *Nature* **432**, 488–492 (2004). 15565150 10.1038/nature03090

[50] Li, J., Naiini, M. M., Vaziri, S., Lemme, M. C. & Östling, M. Inkjet printing of MoS2. *Adv. Funct. Mater.* **24**, 6524–6531 (2014).

[51] Yu, X., Prévot, M. S. & Sivula, K. Multiflake thin film electronic devices of solution processed 2D MoS2 enabled by sonopolymer assisted exfoliation and surface modification. *Chem. Mater.* **26**, 5892–5899 (2014).

[52] Zou, T. et al. High-performance solution-processed 2D P-Type WSe(2) transistors and circuits through molecular doping. *Adv. Mater.* **35**, e2208934 (2023). 36418776 10.1002/adma.202208934

[53] Lin, Z. et al. High-yield exfoliation of 2D semiconductor monolayers and reassembly of organic/inorganic artificial superlattices. *Chem* **7**, 1887–1902 (2021).

[54] Lin, D.-Y., Jheng, J.-J., Ko, T.-S., Hsu, H.-P. & Lin, C.-F. Doping with Nb enhances the photoresponsivity of WSe2 thin sheets. *AIP Advances* **8**10.1063/1.5024570 (2018).

[55] Adel Sedra, K. S., Tony Chan Carusone, Vincent Gaudet. *Microelectronic Circuits*. (Oxford University Press, 2009).

[56] Guerriero, E. et al. Gigahertz integrated graphene ring oscillators. *ACS Nano* **7**, 5588–5594 (2013). 23713626 10.1021/nn401933v

