# Supplementary material for: Electronic properties and circuit applications of networks of electrochemically exfoliated 2D nanosheets
Source: Nat Commun. 2025 Oct 10;16:9038. doi: 10.1038/s41467-025-64100-y (PMC12514219; doi:10.1038/s41467-025-64100-y)
Supplement: Supplementary file 1 — Supplementary Information [file 41467_2025_64100_MOESM1_ESM.pdf]

Supporting Information

Electronic properties and circuit applications of networks of electrochemically exfoliated 2D nanosheets

Tian Carey[1*], Kevin Synnatschke[1], Goutam Ghosh[2], Luca Anzi[3], Eoin Caffrey[1], Emmet Coleman[1], Changpeng Lin[4], Anthony Dawson[1], Shixin Liu[1], Rebekah Wells[1], Mark McCrystall[1], Jan Plutnar[5], Iva Plutnarova[5], Joseph Neilson[1], Nicola Marzari[4], Laurens D.A. Siebbeles[2], Roman Sordan[3], Zdenek Sofer[5], Jonathan N. Coleman[1*]

[1]School of Physics, CRANN & AMBER Research Centres, Trinity College Dublin, Dublin 2,

Ireland

[2]Chemical Engineering Department, Delft University of Technology, Van der Maasweg 9, NL-2629 HZ Delft, The Netherlands

[3]L-NESS, Department of Physics, Politecnico di Milano, Como, Italy

[4]Theory and Simulation of Materials, and National Centre for Computational Design and Discovery of Novel Materials, École Polytechnique Fédérale de Lausanne, Lausanne, Switzerland

[5]Department of Inorganic Chemistry, University of Chemistry and Technology Prague, Technická 5, Prague 6, 166 28, Czech Republic

*Correspondence and requests for materials should be addressed to T.C. (careyti@tcd.ie) and J.C. (colemaj@tcd.ie)

Contents

X-Ray Diffraction (XRD) of 2D Crystals 3
Mechanical Simulation: DFT calculations with vdW correction 7
Atomic Force Microscopy: EE of Elemental, TMM, TMD and TMTs 9
Raman Spectroscopy: Post-Transition Metal Monochalcogenides 11
Raman Spectroscopy: TMDs and Group IV (Post Transition Metal) Dichalcogenides 13
Raman Spectroscopy: Elemental 2D materials 15
Raman Spectroscopy: Transition Metal Trichalcogenides (TMT) 16
UV-Visible Optical Spectroscopy: Elemental 2D materials 17
UV-Visible Optical Spectroscopy: Post-Transition Metal Monochalcogenide 18
UV-Visible Optical Spectroscopy: Transition Metal Dichalcogenide 20
UV-Visible Optical Spectroscopy: Post Transition Metal Dichalcogenide 22
UV-Visible Optical Spectroscopy: Transition Metal Trichalcogenides 23
UV-Visible Optical Spectroscopy: Optical Bandgap Estimation 24
Selection of Nanosheets for Each 2D Material Family 25
Effect of Interlayer Spacing on Crystal Expansion 28
Solid-State Transistors: Indium Telluride and Gallium Telluride 30
Network Capacitance of Ionic Liquid Filled Networks 31
Ionic Current of Liquid Gated Transistors 34
Output Characteristics of Ionic Gated Transistors 35
Further Examination of Network Charge Carrier Densities 36
Terahertz Spectroscopy: Electrochemically Exfoliated Networks 37
Impedance Spectroscopy: Impedance of Semiconducting Nanosheet Networks 38
Impedance Spectroscopy: Measuring Contact Resistance of the Networks 39
Transfer Characteristics of FETs and NMOS 40
Measurement of circuit time constant τ 43
3-bit Digital-to-Analog Circuit 45
4-bit Digital-to-Analog Circuit 46
Amplitude Demodulation of BASK Signal 47
Literature Review of Solution Processed 2D Materials 49
Supplementary References 54

Supplementary Note 1 | X-Ray Diffraction (XRD) of 2D Crystals

X-ray diffraction (XRD) characterisation was performed for crystals prepared in-house. XRD for purchased crystals can be found at the respective manufacturing site. Measurements were taken with a Panalytical X'Pert Pro X-ray diffractometer fitted with a Cu tube emitting K_α radiation at a wavelength of 1.5406 Å. The samples were prepared by grinding crystals using a mortar and pestle and pressing the resulting powder on a glass slide. 2θ diffraction patterns were recorded and the primary peaks were indexed by comparison with reference data from the ICDD PDF-2 database.[1] The raw data, which is sufficient for indexing, is shown below without any additional peak fitting or refinement. A preferred orientation was exhibited by each sample such that enhanced diffraction was observed from planes parallel to the layers in the crystal structure. This is typical for powders of 2D layered crystals and indicates that the particles had a 2D-like platelet morphology, with the long side preferentially aligning with the substrate.[2]

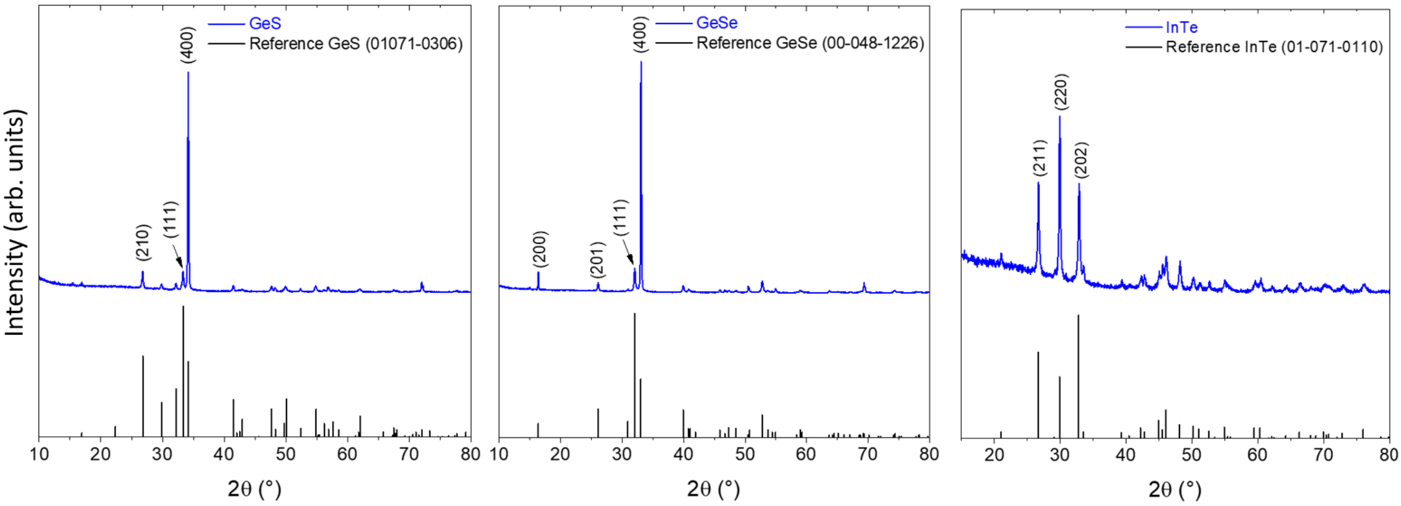

Supplementary Figure 1. XRD spectra for Monochalcogenides GeS (left), GeSe (center), and InTe (right). The strong (004) peaks in GeS and GeSe are consistent with the preferred orientation of the lattice planes of the nanosheets along the glass substrate. InTe appears to show less preferred orientation. The relative intensities of the (221), (220), and (202) peaks suggest the presence of two InTe polymorphs: InTe(I) and InTe(II), of which the latter can only be obtained *via* high pressure[3], which is possible using the methods used in this work (see methods section).

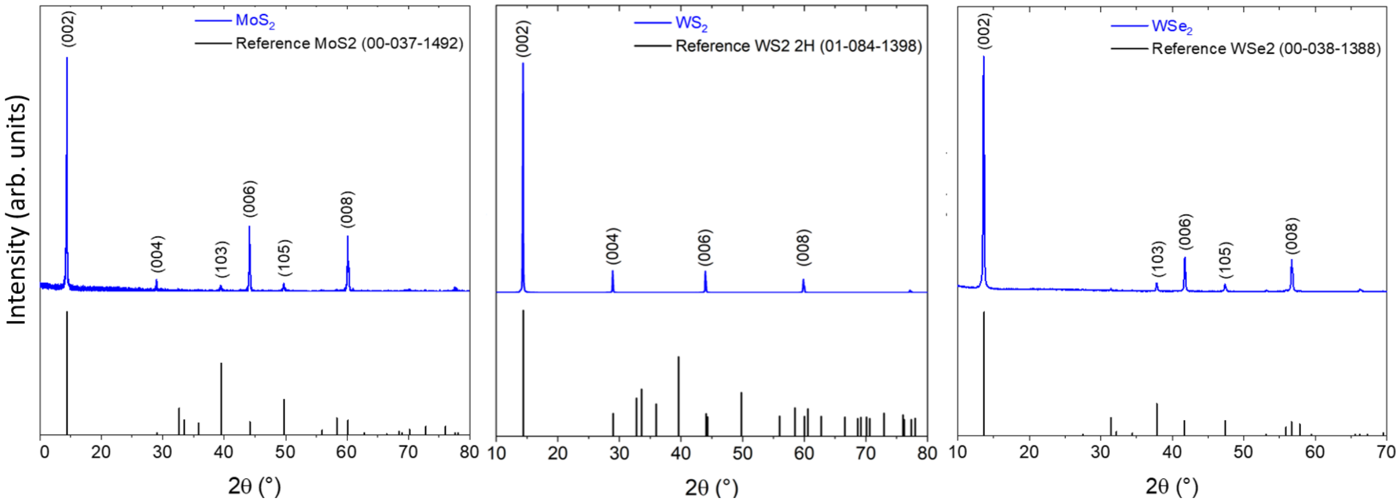

Supplementary Figure 2. XRD spectra for pure phase dichalcogenides MoS_2 (left), WS_2 (center), and WSe_2 (right). The strong (002), (004), (006), and (008) peaks are consistent with the preferred orientation of the lattice planes of the nanosheets along the glass substrate.

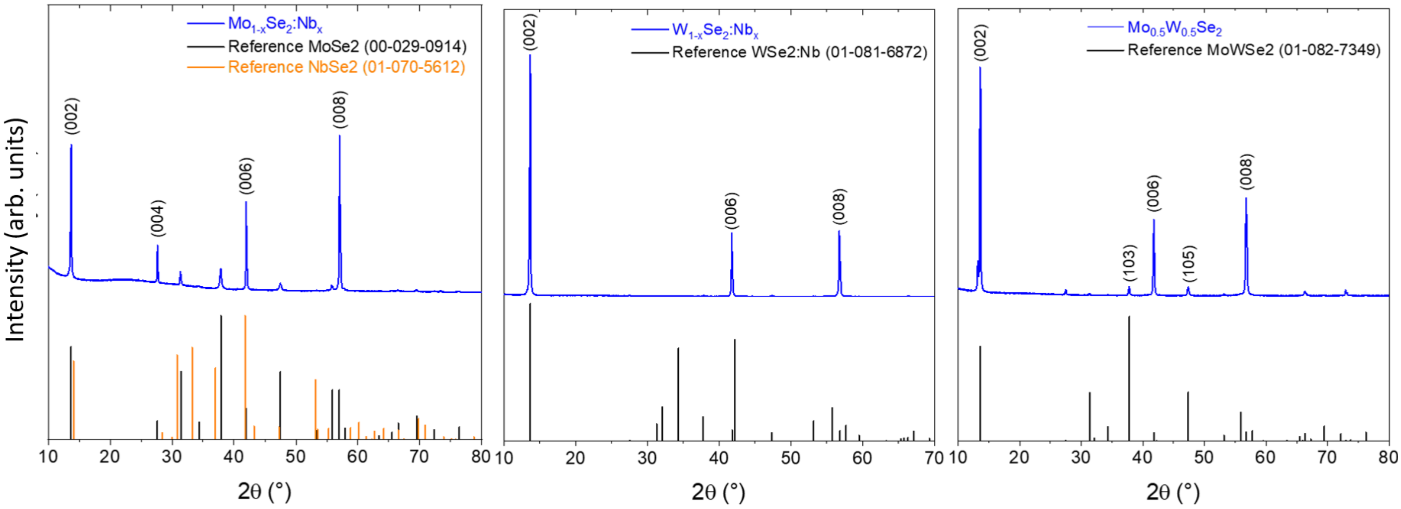

Supplementary Figure 3. XRD spectra for pure doped dichalcogenides $Mo_{1-x}Se_2:Nb_x$ (left) and $W_{1-x}Se_2:Nb_x$ (center) and alloyed dichalcogenide $Mo_{0.5}W_{0.5}Se_2$ (right). The strong (002), (004), (006), and (008) peaks are consistent with the preferred orientation of the lattice planes of the nanosheets along the glass substrate. Importantly, for both the doped samples (left and centre), no pure phase NbS_2 is observed. Furthermore, as shown in the left panel, the peak locations are essentially identical to pure $MoSe_2$, which is consistent with atomic doping. This is in contrast to alloying, where the peak locations should be located clearly between the two pure materials and linearly according to Vegard's law for alloys[4]. For the alloyed sample (right panel), no pure phase material peaks are observed, indicating successful alloying.

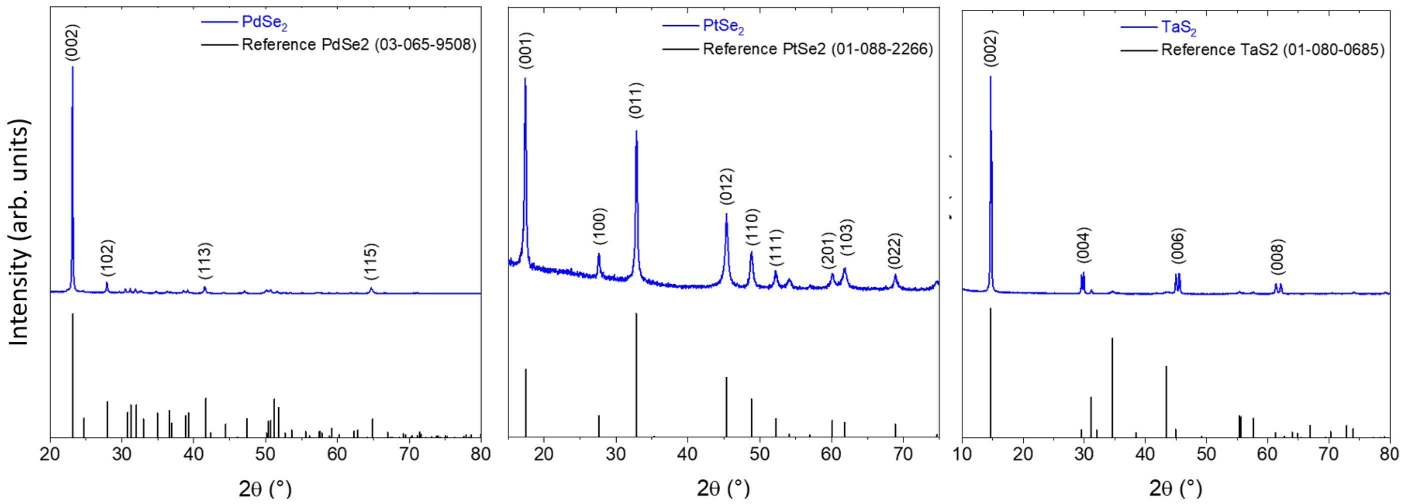

Supplementary Figure 4. XRD spectra for dichalcogenides $PdSe_2$ (left), $PtSe_2$ (center) and TaS_2 (right). For $PdSe_2$, the strong (002) peak is consistent with the preferred orientation of the lattice plate along the glass substrate. For $PtSe_2$ the strong (001) peak is in accordance with preferred orientation. For TaS_2, the strong (002), (004), (006), and (008) peaks are consistent with the preferred orientation of the lattice planes of the nanosheets along the glass substrate.

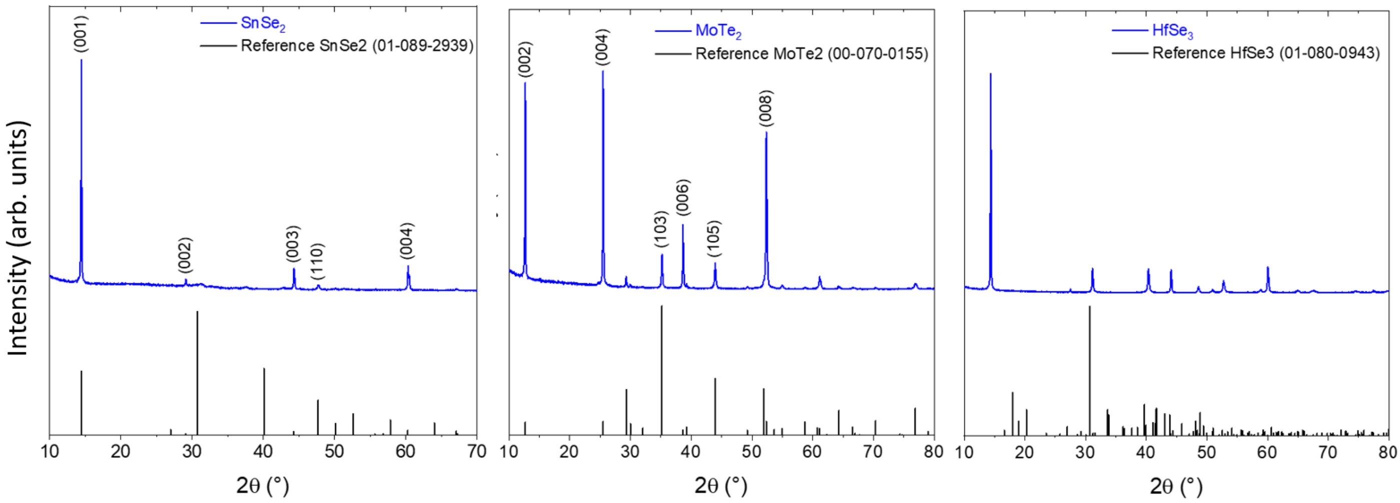

Supplementary Figure 5. XRD spectra for dichalcogenides $SnSe_2$ (left) and $MoTe_2$ (center) and trichalcogenide $HfSe_3$ (right). For $SnSe_2$, the strong peak at (001) is consistent with the preferred orientation along the lattice plane of the nanoplatelet. For $MoTe_2$, strong peaks at (002), (004), (008) are consistent with the preferred orientation. Due to the lack of available experimental data on layered $HfSe_3$, this material was not able to be reliably indexed. However, the peak located around 14° indicates a clear preferred orientation of the nanosheets along the glass substrate, confirming the layered nature of the material. Reference data for an $HfSe_3$ powder (note, not nanoplatelets) is shown for comparison.

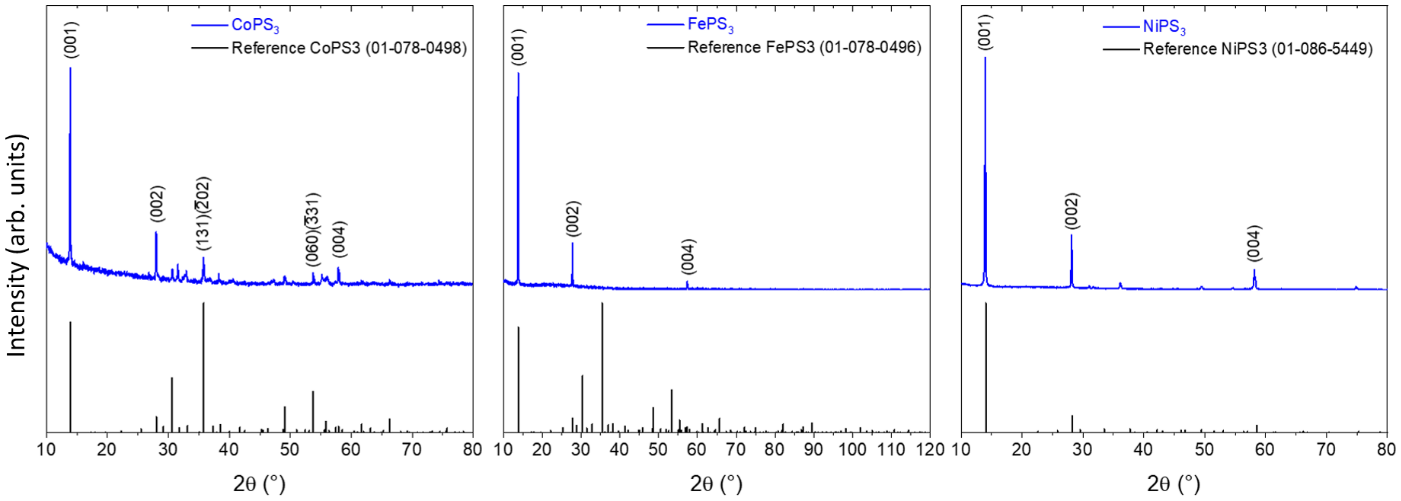

Supplementary Figure 6. XRD spectra for triphosphates $CoPS_3$ (left), $FePS_3$ (center), and $NiPS_3$ (right). The strong (001), (002), and (004) peaks are consistent with the preferred orientation of the lattice planes of the nanosheets along the glass substrate.

Supplementary Note 2 | Mechanical Simulation: DFT calculations with vdW correction

Material	E_b (meV Å^{-2})	C_{11}	C_{22}	C_{12}	C_{33}	E_{IN} (GPa)	E_{OUT} (GPa)	E_{IN}/E_{OUT}
**TMT**								
$FePS_3$	25.5	120	121	34	28	110	28	3.89
$NiPS_3$	20.6	1340*	1350*	380*	441*	124*	44*	2.81
$HfSe_3$	20.3	126	100	14	49	111	49	2.28
$CoPS_3$	24.7	1543*	1534*	502*	461*	1375*	461*	2.98
**TMD**								
MoS_2	28.7	205	205	54	37	191	37	5.18
WS_2	22.7	233	233	55	49	220	49	4.53
WSe_2	22.6	1995*	1995*	404*	526*	191*	53*	3.64
WSe_2:Nb	22.6	1995	1995	404	526	191	53	3.64
$MoSe_2$	20.4	167	167	46	37	155	37	4.16
$MoSe_2$:Nb	20.4	167	167	46	37	155	37	4.16
$PtSe_2$	29.6	167	167	54	30	149	30	5.00
$SnSe_2$	17.3	95	95	28	27	87	27	3.27
TaS_2	22.3	192	192	66	45	170	45	3.78
$MoTe_2$	24.5	120	120	33	41	110	41	2.68
$Mo_{0.5}W_{0.5}Se_2$	23*	2008*	2008*	388*	708*	1933*	708*	2.73
$PdSe_2$	26.78*	1309*	1297*	874*	1296*	72*	130*	0.55
**TMM**								
InSe	14.91	629	629	199	329	57	33	1.72
GaTe	15.7	70	30	19	60	38	60	0.62
SnS	36.2	97	51	42	82	45	82	0.55
SnSe	35.3*	81	53	7	74	64	74	0.87
InTe	38.5*	35	35	14	55	30	55	0.54
TlSe	38.73	40	40	17	65	33	65	0.52
GeSe	36	162	162	7	162	162	162	1.00
GeS	37.3	107	55	2	101	76	101	0.75
**Elemental**								
BP	38.43	185	57	6	54	102	54	1.90
Te	22.92*	30	30	8	60	28	60	0.47
Ge	89.97	108	108	41	108	93	108	0.86
**BCT**								
Bi_2O_2Se	77	156	156	73	121	121	121	1.00

Supplementary Table 1**: Binding energy and mechanical properties of crystals by DFT:** Binding energy and elastic constants from MC2D and JARVIS database, respectively.[5,6] Data marked with * are calculated by our own DFT simulations with vdW correction. Data for Bi_2O_2Se is taken from the literature.[7]

The ratio of in-plane (E_{in}) and out-of-plane (E_{out}) Young's moduli has previously been used to measure exploitability for LPE 2D materials based on the assumption of energy equipartition.[8] The larger the E_{in}/E_{out} ratio, the easier a bulk crystal can be exfoliated into a 2D material. To accelerate the calculation of Young's moduli, we use the Joint Automated Repository for Various Integrated Simulations (JARVIS) database to find the elastic constants related to the material's response to stress in each crystal.[5,6] The elastic constants (C_{11}, C_{22}, C_{12}, and C_{33}) in the database are obtained using density functional theory (DFT) with van der Waals (vdW) corrections considered to provide more accurate material properties. When the vdW direction is

oriented along the z-axis, the out-of-plane Young's modulus E_{out} is denoted by the elastic constant C_{33}. The in-plane Young's modulus E_{in} can be calculated by,

$$E_{in} = \frac{C_{11}C_{22} - C_{12}^2}{\sqrt{C_{11}C_{22}}} \quad (2)$$

Another measure previously used to predict the exfoliatibility of 2D materials is the binding energy, E_b, defined as the difference between the ground-state energy of the relaxed 3D bulk structure and all of its unrelaxed substructures of any dimensionality per unit area.[9,10] Previous DFT work categorised materials with binding energy below 120 meV Å^{-2} as potentially exploitable, and materials that have higher binding energies are non-exfoliative.[10] We use the materials cloud database to determine E_b of each material presented in Supplementary Table 1.

Materials which did not have sufficient information in the database were simulated within the DFT framework using the Quantum ESPRESSO package[11,12] and the v1.1 SSSP efficiency pseudopotentials' library[13]. We employed the Perdew, Burke and Ernzerhof's (PBE)[14] parametrisation of the generalised gradient approximation (GGA) to describe the exchange-correlation effect of electrons. The vdW functionals at the vdw-df2-c09[15] and rVV10[16] levels were adopted for non-magnetic and magnetic materials, respectively, according to previously described protocols.[9] All simulations were performed with the suggested plane-wave energy cutoff of the SSSP library and a k-point density of 0.15 Å^{-1}, and a Marzari−Vanderbilt cold smearing[17] of 0.02 Ry was further used to converge the Brillouin zone integral in metallic systems. For elastic constant calculations, we used the finite difference of the strain-stress relation as implemented in thermo_pw code,[18] where the independent strains with a magnitude of 0.005 were applied to deform the system depending on the Laue class and crystal symmetry. The elastic stiffness tensor was then calculated as the first-order derivative of stress with respect to strain.

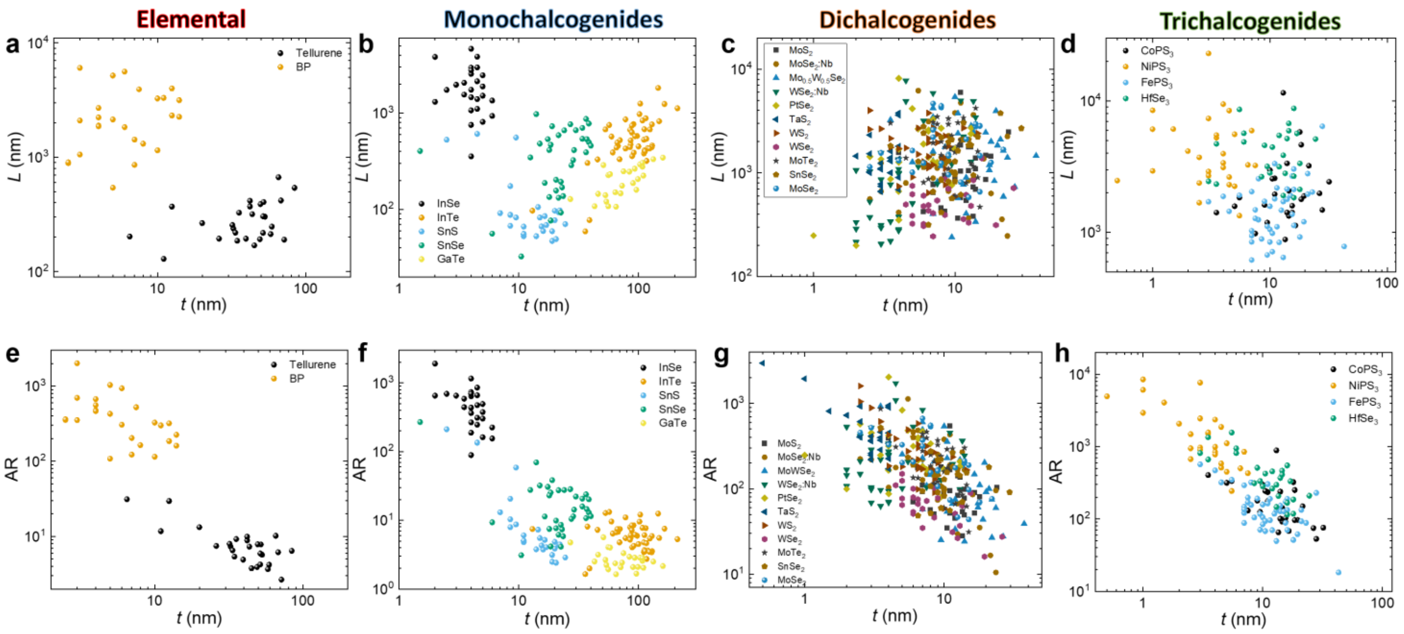

Supplementary Figure 7: **Atomic force microscopy of 2D material nanosheets. a-d** Lateral size (*L*) and apparent thickness (*t*) of the elemental, monochalcogenide, dichalcogenide and trichalcogenide 2D nanosheets. **e-h** Aspect ratio (i.e. *L*/*t*) of the elemental, monochalcogenide, dichalcogenide and trichalcogenide 2D nanosheets.

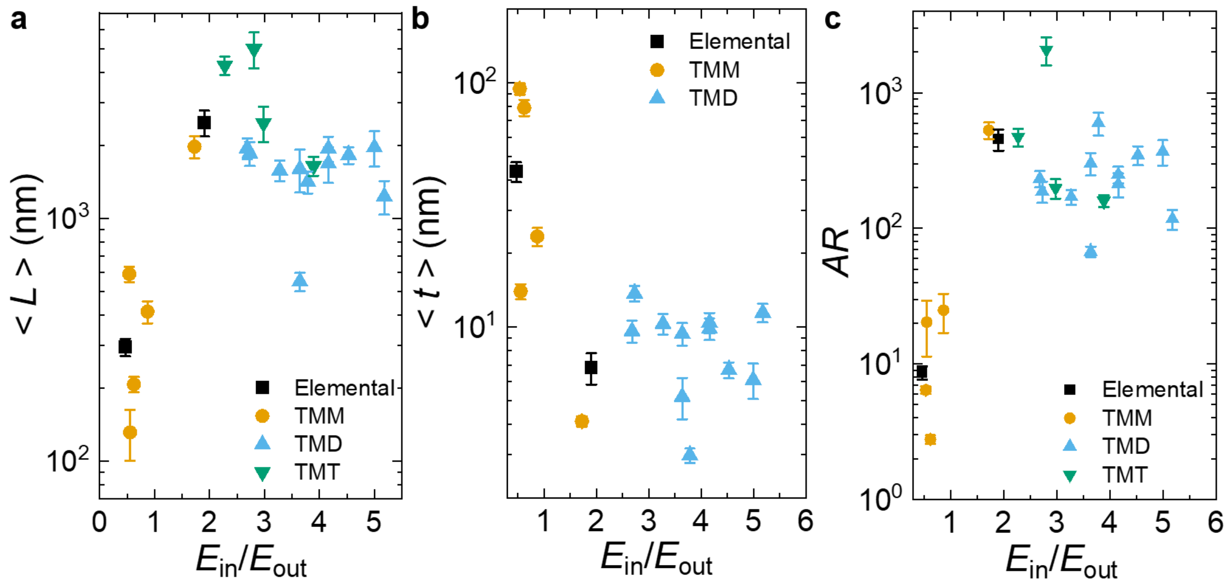

Supplementary Figure 8: **Predicted crystal mechanical properties combined with atomic force microscopy for each material. a** Lateral size (*L*), **b** apparent thickness (*t*) and **c** nanosheet aspect ratio (*AR*) of the elemental, monochalcogenide, dichalcogenide and trichalcogenide 2D nanosheets as a function of the ratio of in-plane young's modulus to out-of-plane young's modulus. Error is calculated by SDOM, for each material n = 25, except for SnSe, InTe, $FePS_3$ where n = 35, 52, 45 and respectively and the TMD's $Mo_{0.5}W_{0.5}Se_2$, WSe_2:Nb and WSe_2, which have n = 33.

Material	<*L*> (nm)	<*t*> (nm)	<AR>
**BP**	**2477** ± 303	**7** ± 1	**456** ± 81
**Tellurene**	**294** ± 24	**43** ± 4	**9** ± 1
**GaTe**	**207** ± 15	**79** ± 6	**3** ± 1
**InSe**	**1971** ± 206	**4.0** ± 0.2	**531** ± 74
**InTe**	**589** ± 43	**95** ± 5	**6** ± 1
**SnS**	**131** ± 31	**14** ± 1	**20** ± 9
**SnSe**	**412** ± 43	**23** ± 2	**25** ± 8
**$MoSe_2$**	**1937** ± 232	**10** ± 1	**250** ± 35
**$MoSe_2$:Nb**	**1683** ± 285	**10** ± 1	**212** ± 43
**WSe_2**	**549** ± 48	**9** ± 1	**67** ± 6
**WSe_2:Nb**	**1598** ± 321	**5** ± 1	**303** ± 57
**MoS_2**	**1230** ± 196	**11** ± 1	**117** ± 20
**$Mo_{0.5}W_{0.5}Se_2$**	**1851** ± 211	**14** ± 1	**188** ± 33
**$PtSe_2$**	**1960** ± 325	**6** ± 1	**370** ± 78
**WS_2**	**1818** ± 146	**7** ± 1	**347** ± 55
**$MoTe_2$**	**1942** ± 192	**10** ± 1	**233** ± 32
**$SnSe_2$**	**1576** ± 152	**10** ± 1	**171** ± 21
**TaS_2**	**1414** ± 148	**3.0** ± 0.2	**600** ± 115
**$NiPS_3$**	**4988** ± 842	**4.0** ± 0.3	**2084** ± 448
**$CoPS_3$**	**2467** ± 409	**15** ± 1	**198** ± 33
**$FePS_3$**	**1643** ± 147	**12** ± 1	**160** ± 16
**$HfSe_3$**	**4268** ± 376	**12** ± 1	**473** ± 72

Supplementary Table 2: **Summary of atomic force microscopy results.** Average lateral size <L>, average apparent thickness <t> and average aspect ratio <AR> for each of the elemental (red), monochalcogenide (blue), dichalcogenide (orange) and trichalcogenide (green) 2D nanosheets. Error is calculated by SDOM, for each material n = 25, except for SnSe, InTe, $FePS_3$ where n = 35, 52, 45 and respectively and the TMD's $Mo_{0.5}W_{0.5}Se_2$, WSe_2:Nb and WSe_2 which have n = 33.

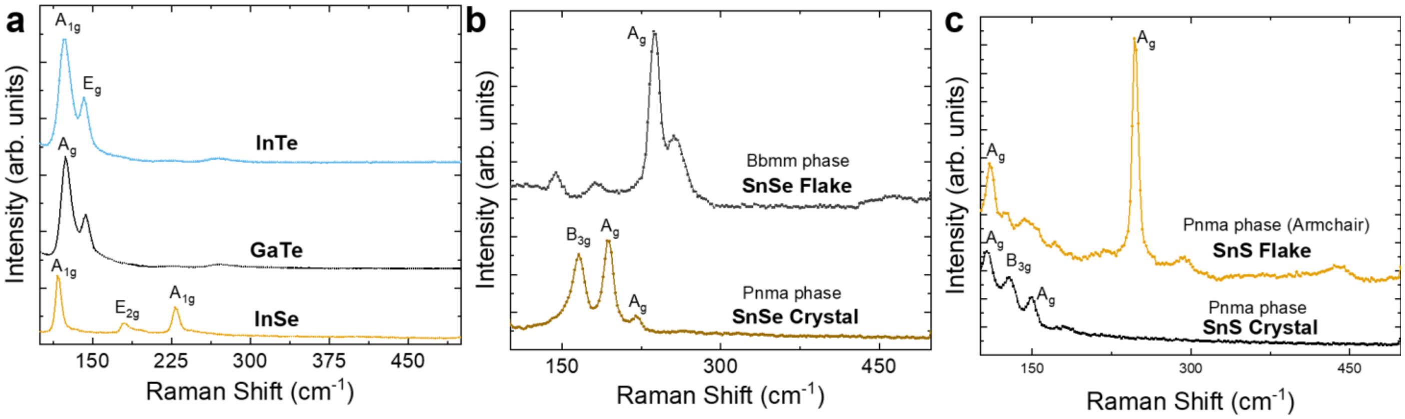

Supplementary Figure 9: **Raman spectroscopy of post-transition metal monochalcogenides. a** Raman spectroscopy of the PTMM materials. **b** Raman spectra of the SnSe starting bulk crystal (brown) and the exfoliated SnSe nanosheets (black) **c** Raman spectra of the SnS starting bulk crystal (black) and the electrochemically exfoliated SnS nanosheet (yellow).

Supplementary Figure 9a (yellow curve) shows that the vibrational modes of the A_{1g}, E_{2g} and A_{1g} are found at ~117, 180 and 228 cm^{-1}, respectively, indicative of few-layer InSe. The A_{1g} mode is associated with the out-of-plane vibrations of the Se atoms, while the E_{2g} mode is due to in-plane vibrations between the In and Se in the basal plane.[19] Supplementary Figure 9a (blue curve) shows the two expected vibrational modes at 122 (A_{1g}) and 141 cm^{-1} (E_g) for indium telluride (InTe).[20] Very little has been investigated regarding the raman vibrational modes of InTe, the A_{1g} mode is likely associated with the out-of-plane vibrations of the Te atoms and the E_g mode associated with the in-plane vibrations between the In and Te atoms, similar to the other group III monochalcogenides.

Supplementary Figure 9a (black curve) shows the Raman spectra of the gallium telluride (GaTe) sample, which shows the A_g vibrational mode at 124 cm^{-1}.[21] Unlike freshly cleaved GaTe, which has additional peaks >150 cm^{-1}.[22] The peak at 143 cm^{-1} is attributed to the surface's adsorbed oxygen (GaTe-O_2).[22] We exfoliated our GaTe in a glovebox and used degassed solvents to exfoliate. However, the oxygen could have been adsorbed during the Raman measurement in the air or small quantities of O_2 <0.1 ppm could have been adsorbed over time while stored in the glove box. The InTe, GaTe and InSe spectra are consistent with measurements on liquid-phase exfoliated nanosheets.[20]

We undertook an additional Raman spectroscopy investigation on the tin-based group IV monochalcogenides since they can exist in various phases and polytypes due to the different oxidation states of Sn and consequently have vastly different electronic properties.[23] In Supplementary Figure 9b, the SnSe crystal before exfoliation shows B_{3g} and A_g peaks (brown curve) associated with the semiconducting pnma phase of the SnSe crystal. After electrochemical exfoliation (black curve), the phase of the material changes to the

semimetallic bbmm phase, which is attributed to the quenching of the B_{3g} mode and upshift of the A_g peak to 236 cm^{-1}.[24]

We also investigated tin sulfide (SnS) nanosheets in Supplementary Figure 9c and found the prominent A_g and B_{3g} modes expected for SnS crystals.[23] Once exfoliated the A_g peak increases in intensity and shifts to 246 cm^{-1} and has an absence of the B_{3g} peak which is indicative of SnS with an armchair configuration rather than zigzag.[25] Therefore, the pnma phase is retained.[26]

Supplementary Note 5 | Raman Spectroscopy: Transition Metal Dichalcogenides and Group IV (Post Transition Metal) Dichalcogenides

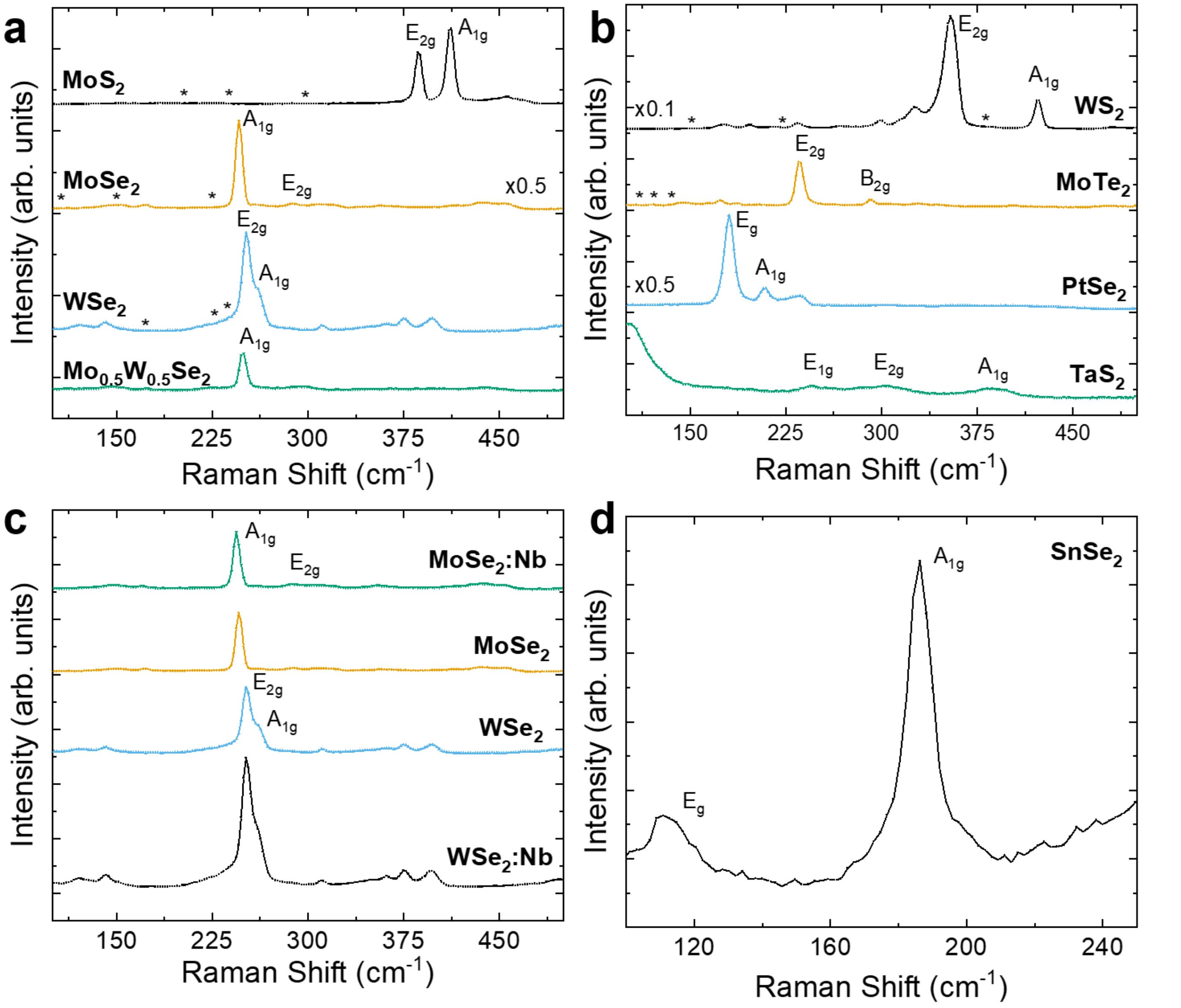

Supplementary Figure 10: **Raman spectroscopy of Transition Metal Dichalcogenides and Group IV (Post Transition Metal) Dichalcogenides. a,b** Raman spectroscopy of the TMD's **c** Raman spectra of the niobium doped TMDs **d**. Raman spectra of SnSe2 a post transitional metal dichalcogenide. The spectra in all cases are consistent with reports of the semiconducting 2H phase and the J1, J2 or J3 vibrational modes attributed to the metallic 1T phases are not observed. We mark the absent peaks with a star (*).

Next, we investigated the phase and quality of the transition metal and group IV dichalcogenides used in the study. Supplementary Figure 10a depicts the spectra of the MoS_2 (black), $MoSe_2$ (yellow), WSe_2 (blue) and the alloy $Mo_{0.5}W_{0.5}Se_2$ (green) nanosheets after drop casting and annealing at 120 °C on Si/SiO_2. The black MoS_2 spectrum (Supplementary Figure 10a, black curve) shows the typical E_{2g} and A_{1g} peaks at 385 cm^{-1} and 412 cm$^{-1,}$ respectively.[27,28] The Raman spectrum of the $MoSe_2$ (Supplementary Figure 10a, yellow curve) has two peaks located at ~ 245 and 287 cm^{-1} attributed to the A_{1g} and E_{2g} Raman modes.[29]

The WSe_2 (Supplementary Figure 10a, blue curve) has a peak at (~ 251 cm^{-1}) attributed to the A_{1g} and E_{2g} Raman modes, indicating the formation of few-layer nanosheets.[30,31] Our $Mo_{0.5}W_{0.5}Se_2$ has a ratio of 0.5:0.5 (Mo:W). In Supplementary Figure 10a, (green curve) we find the A_{1g} vibrational mode at ~ 247 cm^{-1} attributed to the out of plane motion of the Se atoms relative to a Mo or W atom and a weak E_{2g} vibration at ~ 146 cm^{-1} attributed to the in-plane vibrational motion of the Se atoms.[32]

The Raman spectrum of the WS_2 (Supplementary Figure 10b, black) also matches the previous literature reports for few-layer WS_2, having an A_{1g} peak (~ 421 cm^{-1}) and an overlapping 2LA and E_{2g} peak at 354 cm^{-1}.[33] The Raman spectrum of the $MoTe_2$ nanosheets are shown in Supplementary Figure 10b, yellow curve and demonstrate in-plane vibrational modes (E_{2g}) at ~235 cm^{-1} and a B_{2g} at ~ 290 cm^{-1}. The presence of the B_{2g} mode indicates that we have few layer $MoTe_2$,[34] since the B_{2g} mode is not observed in bulk.[35]

For the MoS_2, $MoSe_2$, $MoTe_2$ WS_2 and WSe_2, the J_2 and J_3 vibrational modes attributed to the metallic 1T phase are not observed.[36-41] We plot the Raman spectra for $PtSe_2$ in Supplementary Figure 10b, blue curve. We observe the A_{1g} and E_g peaks at ~207 cm^{-1} and ~180 cm^{-1} respectively. The E_g peak arises due to the vibration of selenium (Se) atoms within the plane, while the A_{1g} peak results from the vibration of Se atoms perpendicular to the plane.[42] The intensity ratio of A_{1g}/E_g is ~ 0.2 and consistent with the ratio expected for ultra-thin <3 nm $PtSe_2$.[43] Similarly the peak positions of the A_{1g} and E_g modes would suggest nanosheets that are <5 nm.[44] These results are consistent with our AFM results (main text), which show an apparent thickness of ~ 3 nm due to the favourable mechanical properties of PtSe2 for electrochemical exfoliation.

The Raman spectra of TaS_2 are plotted in Supplementary Figure 10b, green. We observe the E_{1g}, E_{2g} and A_{1g} vibrational modes at ~ 244, 301 and 385 cm^{-1} respectively. The E_{1g} modes represent the in-plane vibrations of the Se atoms, while the E_{2g} mode represents the in-plane vibrations of the S and Ta atoms. The A_{1g} mode represents the out-of-plane vibration of the S atoms.[45] The peak positions of the vibrational modes are consistent with what would be expected for 2H-TaS_2,[46]

We undertake Raman spectroscopy of the niobium-doped TMD's WSe_2 and $MoSe_2$ in Supplementary Figure 3c. We don't observe any differences between the doped WSe_2 and $MoSe_2$ and their undoped counterparts. This is not unexpected since metals do not typically have observable Raman peaks.[47]

Next, we examine $SnSe_2$ by raman spectroscopy which is a post-transition metal dichalcogenide. We observe the in-plane E_g vibrational mode at ~111 cm^{-1} and the out-of-plane A_{1g} vibrational mode at ~ 186 cm^{-1} consistent with previous reports.[48] 2H-$SnSe_2$ the E_g peak is located at 108 cm^{-1} (in theory) while 1T-$SnSe_2$, gives rise to an upshift of the E_g peak to ~119 cm^{-1}.[48,49] Therefore we have electrochemically exfoliated the 2H-$SnSe_2$ phase.

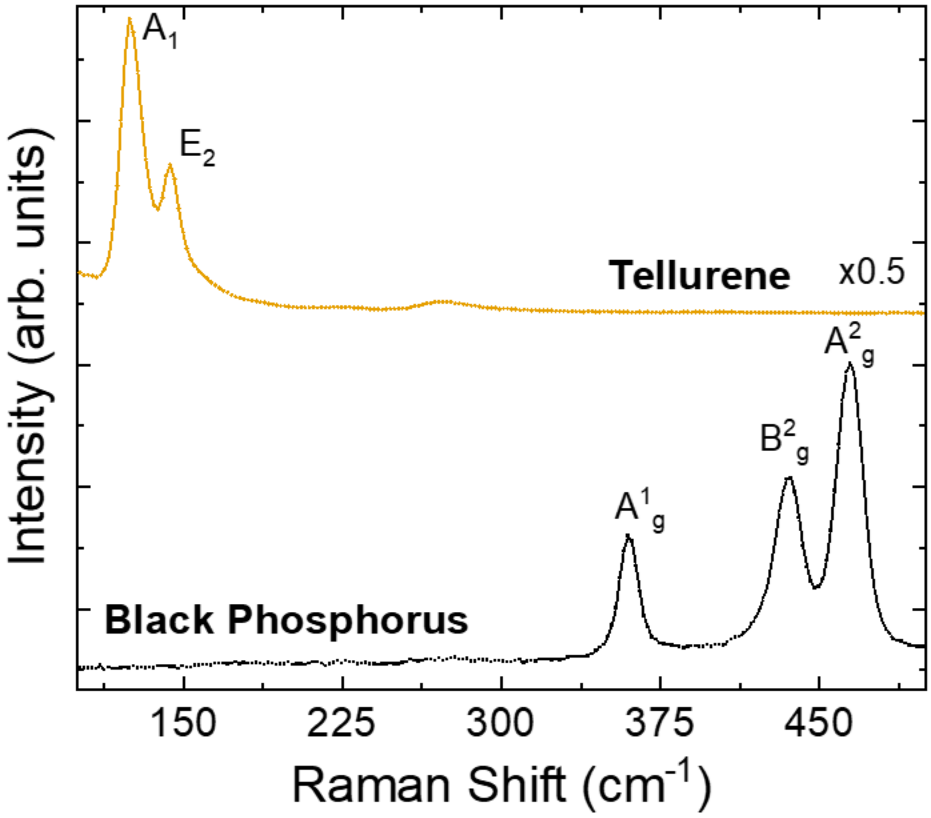

Supplementary Figure 11: **Raman spectroscopy of the elemental 2D materials electrochemically exfoliated.** Raman spectroscopy of the elemental 2D materials black phosphorus and tellurene labelled with their corresponding vibrational modes.

We examine the Raman spectra of the elemental 2D materials in Supplementary Figure 11. Black Phosphorus (BP) (black curve) shows the A^1_g, B^2_g and A^2_g vibrational modes at 360, 435 and 465 cm^{-1} respectively. The A^2_g and B^2_g modes are related to in-plane vibrations of the phosphorus and the A^1_g mode is related to the out-of-plane movement of the phosphorous.[50] The peak positions are upshifted compared to bulk BP samples, which have A^1_g and A^2_g vibrational modes at 359 and 463 cm^{-1} which suggests the BP nanosheets are thin with nanosheet thickness <10 nm,[51] consistent with our AFM results presented in the main text.

Next, we examine our tellurene nanosheets in Supplementary Figure 11 (yellow curve) and find the A_1 (124 cm^{-1}) and E_2 (144 cm^{-1}) vibrational modes, which are typical of tellurene and attributed to Te atoms moving in the basal plane (A_1) and asymmetric stretching (E_2) of Te atoms.[52] The presence of the E_2 mode would suggest that the nanosheets are >20 nm thick (consistent with our AFM results), which is not active for monolayer or few-layer nanosheets.[53]

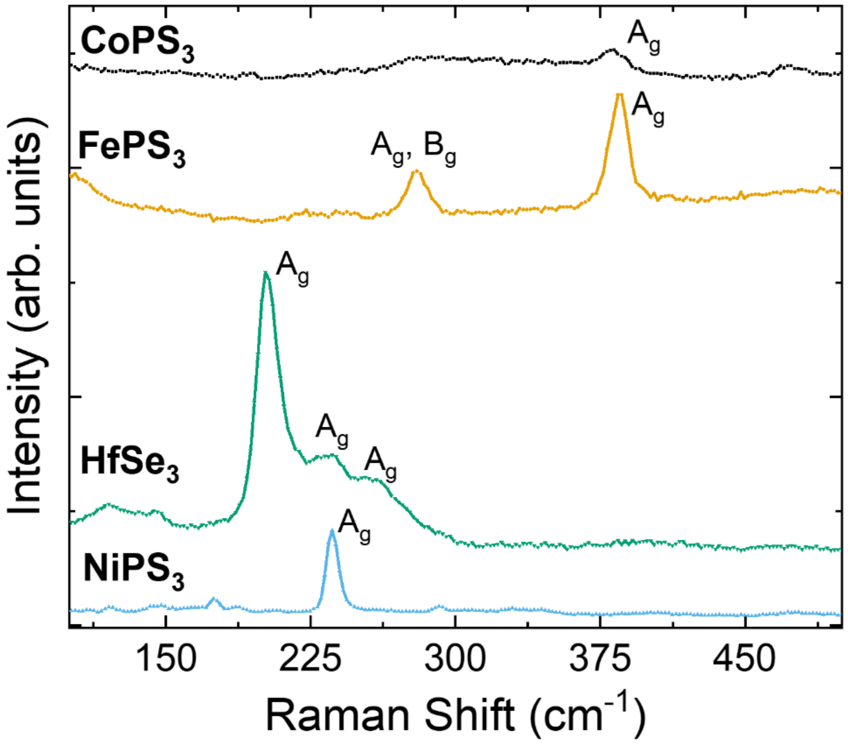

Supplementary Figure 12: **Raman spectroscopy of the electrochemically exfoliated transition metal trichalcogenide and layered metal phosphorus trichalcogenides.** Iron phosphorus trisulfide, cobalt phosphorus trisulfide, nickel phosphorus trisulfide and hafnium triselenide are labelled with their corresponding vibrational modes.

We undertake raman of the transition metal trichalcogenides and layered metal phosphorus trichalcogenides in Supplementary Figure 12. The Raman spectroscopy of hafnium triselenide ($HfSe_3$) (green curve) shows a A_g peaks at 202 cm^{-1}, 232 cm^{-1} and 253 cm^{-1} which are likely attributed to the A_g vibrational mode.[54] Iron phosphorus trisulfide ($FePS_3$) spectra (yellow curve) show Raman peaks at 280 cm^{-1} and 384 cm^{-1} which are attributed to the A_g and B_g vibrational modes.[55] Nickle phosphorus trisulfide ($NiPS_3$) spectra (blue) show one peak at 236 cm^{-1} attributed to the out-of-plane A_g vibrational mode. As the layer number in $NiPS_3$ increases from monolayers to bulk, the Raman active peaks increase in intensity,[56] which has previously been attributed to constructive interference enhancement.[56] Cobalt phosphorus trisulfide ($CoPS_3$) spectra (black curve) display a single peak at 382 cm^{-1} which is expected for ultra-thin $CoPS_3$ nanosheets and is attributed to the A_g vibrational mode.[57]

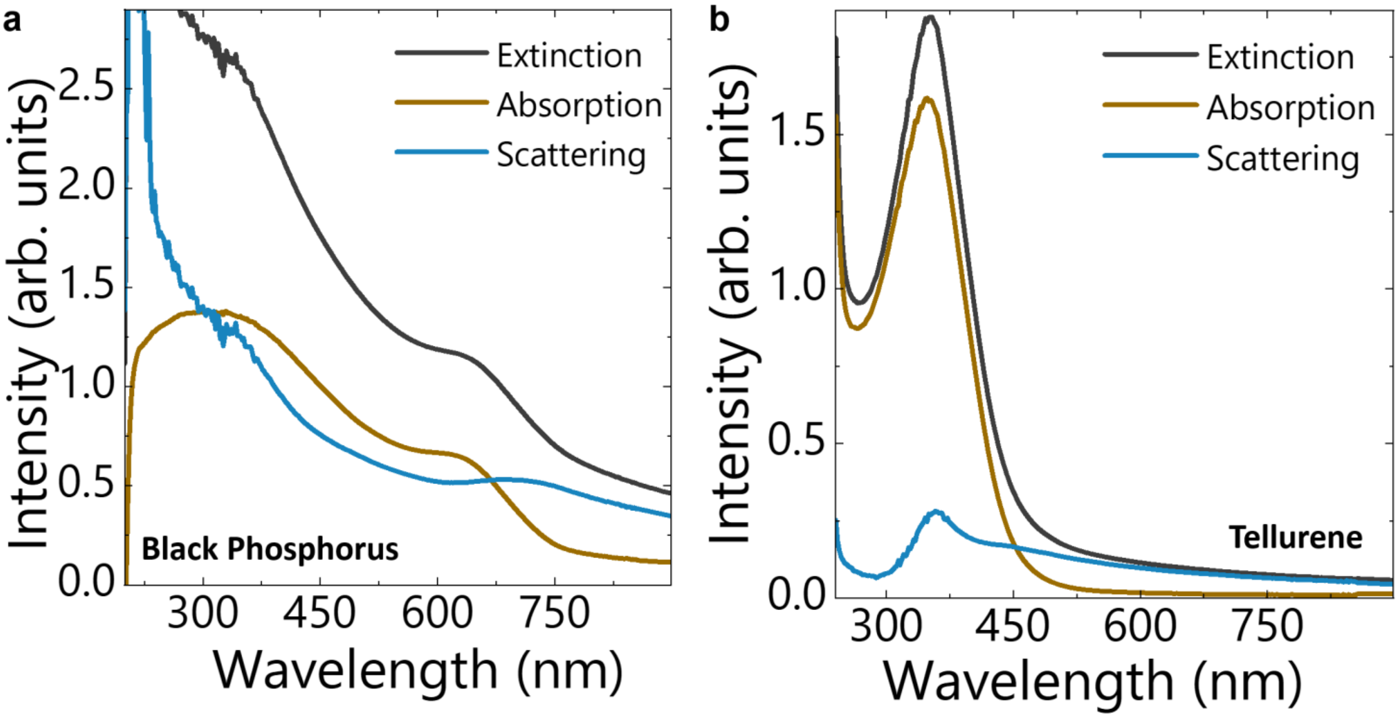

Supplementary Figure 13: **UV-visible optical absorption spectra of the electrochemically exfoliated elemental 2D materials. a** UV-vis spectra of black phosphorus and **b** tellurene with the characteristic absorption peaks shown.

Supplementary Figure 13 presents a selection of the UV-visible optical absorption for black phosphorus (BP) obtained using an integrating sphere where we have subtracted the scattering component of each spectrum from the extinction spectra to obtain the absorption spectra.[58] We observe a broad absorption band across the UV and NIR wavelengths, which is consistent with previous reports for BP nanosheets made by LPE.[59] There is also a characteristic absorption peak at ~ 620 nm, which has not previously been observed. Figure 1b shows the spectra of tellurene and reveals an excitonic transition at 348 nm attributed to the transition of valence band p-orbital charge carriers to the conduction band.[60]

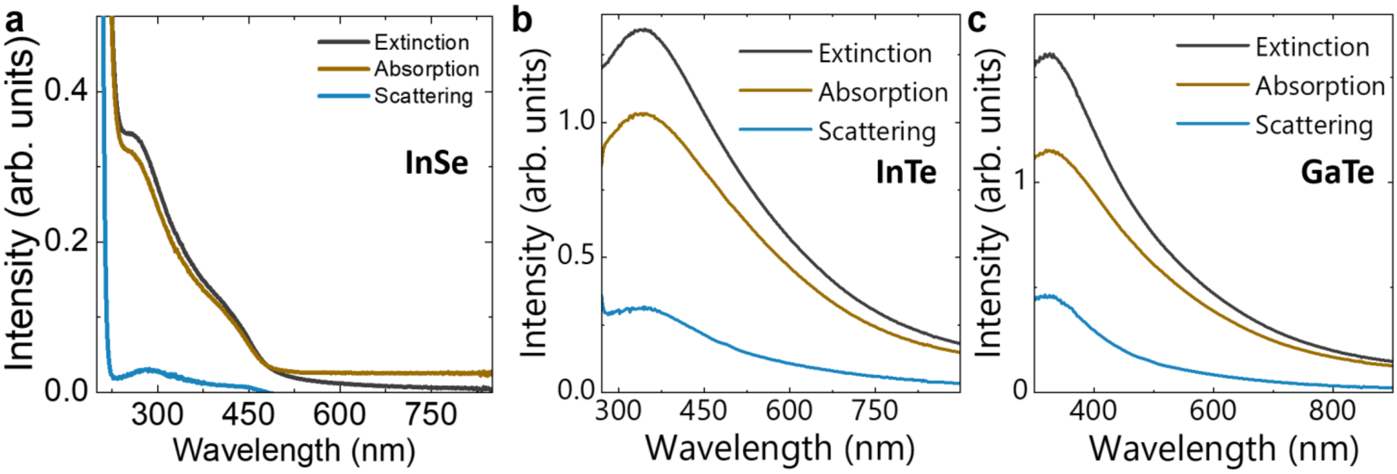

Supplementary Figure 14: **UV-visible optical absorption spectra of the electrochemically exfoliated Post-Transition Metal Monochalcogenides. a** UV-vis spectra of indium selenide, **b** indium telluride and **c** gallium telluride with the characteristic absorption peaks shown.

Supplementary Figure 14 presents the UV-visible optical absorption for indium selenide (InSe), which shows a broad peak at ~260 nm, which is consistent with the expected peak position from DFT calculations.[61] Supplementary Figure 14b shows the spectra obtained for indium telluride (InTe) it is a characteristic absorption peak at ~342 nm, which is consistent with DFT calculations for the expected peaks.[61] Supplementary Figure 14c shows the spectra obtained for gallium telluride (GaTe), which shows a characteristic absorption peak at ~324 nm, the featureless tail is consistent with previous reports.[62]

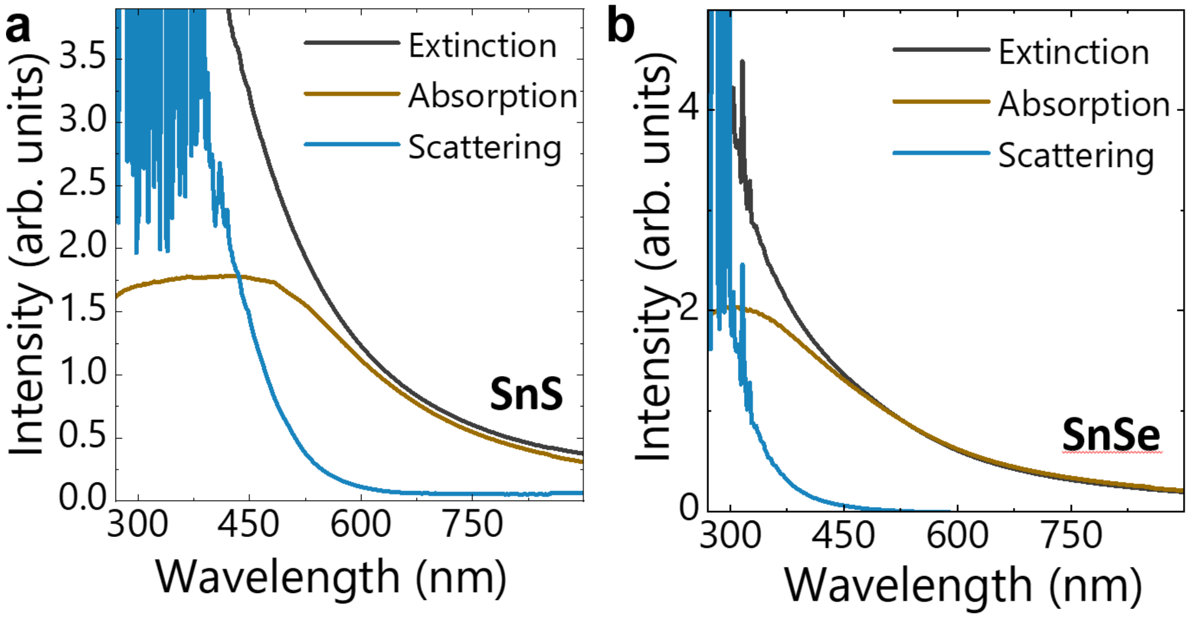

Supplementary Figure 15: **UV-visible optical absorption spectra of the electrochemically exfoliated tin-based metal monochalcogenides. a** UV-vis spectra of tin sulfide and **b** tin selenide with the characteristic absorption peaks shown.

Supplementary Figure 15a presents the UV-visible optical absorption for tin sulfide (SnS), which shows a broad absorption that extends from 270 nm to 480 nm, which is consistent with previous reports.[63] The scattering background is also small at wavelengths above 600 nm, suggesting the presence of small nanosheets, consistent with our AFM data in the main text. Supplementary Figure 15b presents the UV-visible optical absorption for tin selenide (SnSe), which shows a broad absorption that extends from 270 nm to 480 nm, which is also consistent with previous reports.[64] Similar to the SnS nanosheet, the SnSe had a small scattering component >450 nm, which is attributed to the size of the nanosheets.

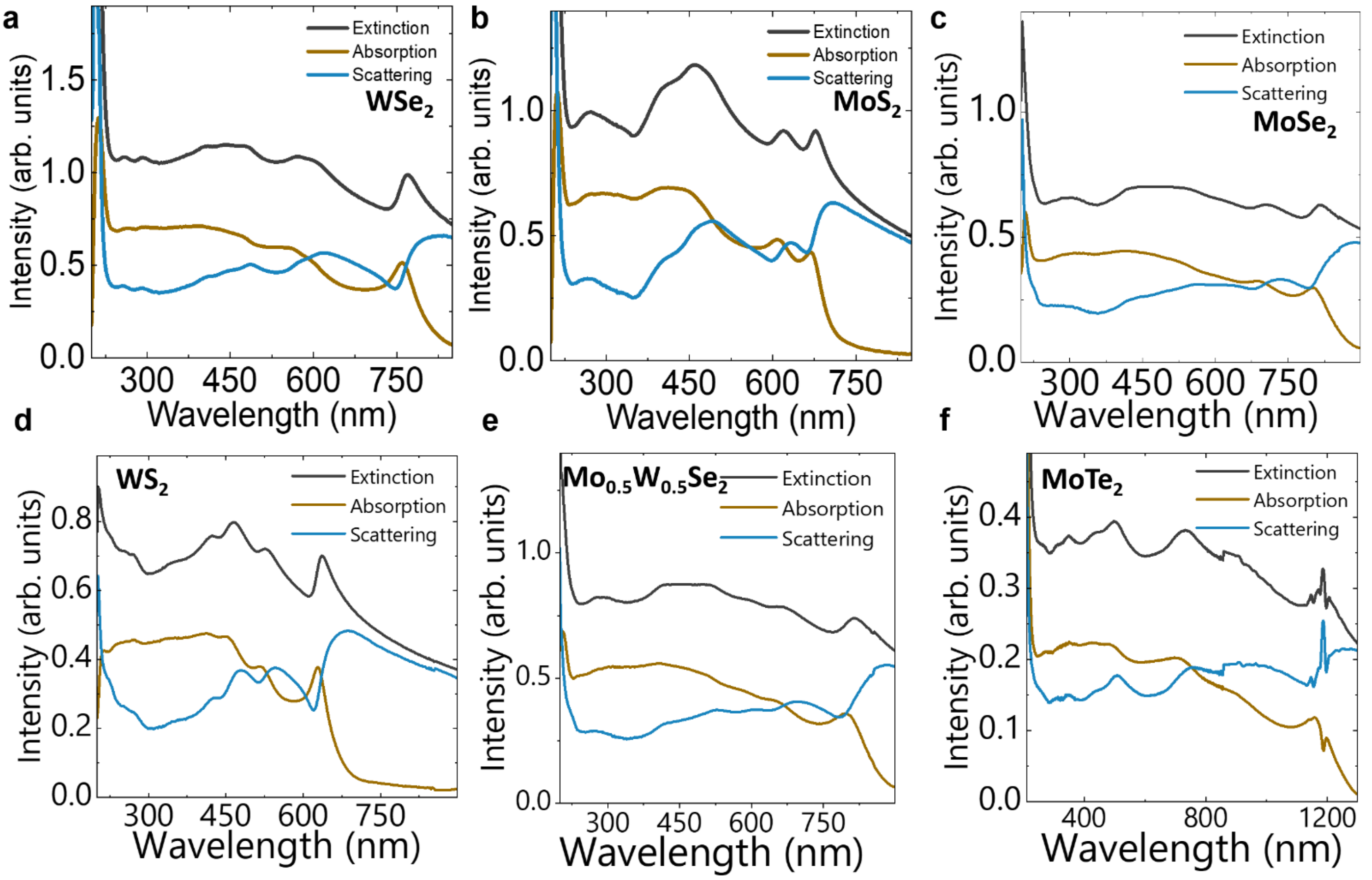

Supplementary Figure 16: **UV-visible optical absorption spectra of the electrochemically exfoliated transition metal dichalcogenides. a** UV-vis spectra tungsten diselenide (WSe_2), **b** molybdenum disulfide (MoS_2), **c** molybdenum diselenide ($MoSe_2$), **d** tungsten disulfide (WS_2), **e** molybdenum tungsten diselenide ($Mo_{0.5}W_{0.5}Se_2$), **f** molybdenum ditelluride ($MoTe_2$) with the extinction, absorption and scattering components of the UV-vis spectra shown in black, brown and blue for each material respectively.
In Supplementary Figure 16a-f, UV-visible optical absorption spectra of the WSe_2, MoS_2, $MoSe_2$, WS_2, $MoTe_2$, and $Mo_{0.5}W_{0.5}Se_2$ inks are taken with an integrating sphere to isolate the extinction, absorption and scattering components of the TMD inks.[58] The excitonic peak positions of the materials are consistent with previous reports for well-known TMD's displaying the A and B excitons WSe_2, MoS_2, $MoSe_2$, WS_2 and $MoTe_2$ and are consistent with previous reports.[34,65-67] The $Mo_{0.5}W_{0.5}Se_2$ spectra show excitonic peaks that are similar to $MoSe_2$ that correspond to the A and B excitons at 798 nm and 658 nm, respectively.[66] In all cases, the scattering component is large and ~50% of the extinction spectra intensity, which would suggest a large nanosheet *L*. The ratio of the extinction at the B exciton peak (Ext_B) to the local minimum at 345 nm (Ext_{345}) in the MoS_2 spectral analysis (refer to Supplementary Figure 16b, black curve) serves as a metric for determining *L*.[58] The $Ext_B/Ext_{345} > 1$ indicates that the nanosheets are large with $L > 400$ nm.[58] Similarly, the length *L* of WS_2, $MoSe_2$ and WSe_2 can be gauged by the ratio of extinction at the A exciton peak (Ext_A) to the local minimum. For WS_2 the local minimum is found at 295 nm (Ext_A/Ext_{295}).[58] We find $Ext_A/Ext_{295} > 1$

indicating L > 400 nm.[58,68] For $MoSe_2$ and WSe_2 the extinction at 365 nm and 334 nm can be used to indicate we have larger sized nanosheets with Ext_A/Ext_{365} > 0.4 and Ext_A/Ext_{334} > 0.22 respectively.[58] The size metrics determined by UV-vis are in agreement with our AFM statistics from the main text.

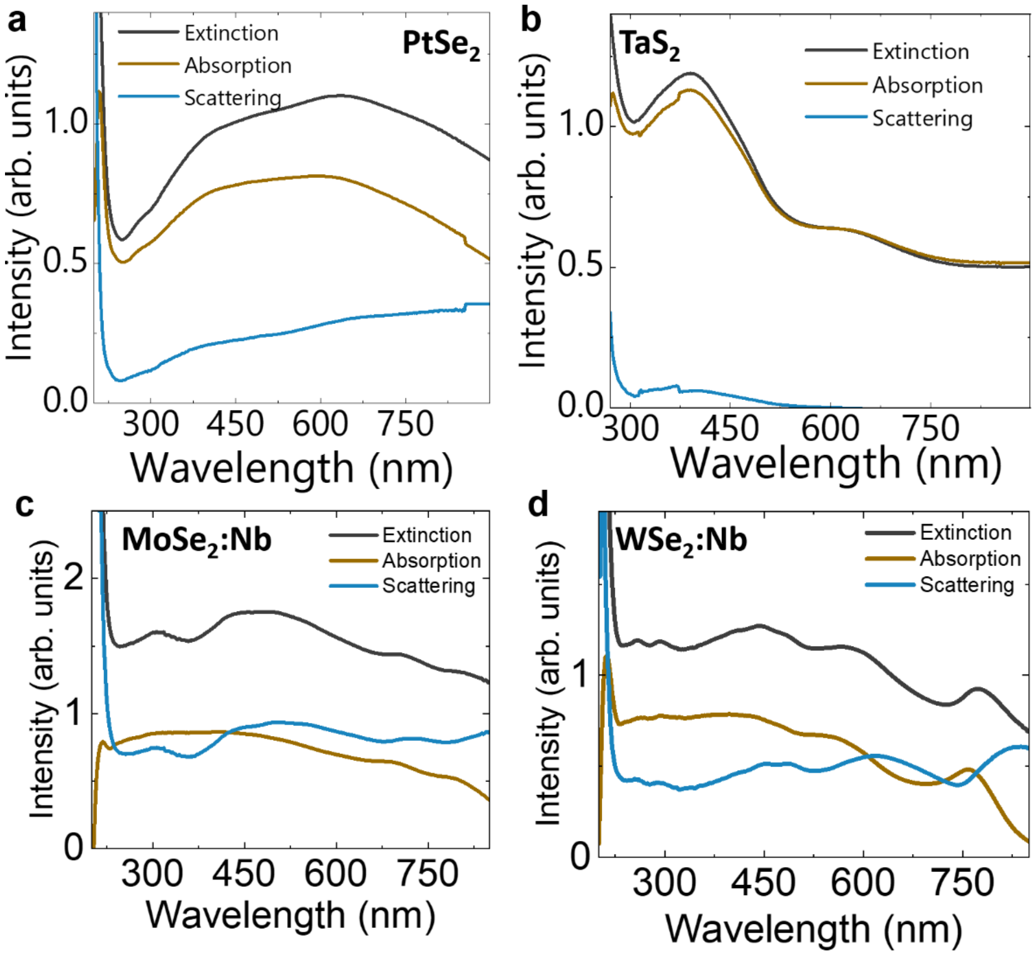

Supplementary Figure 17: **UV-visible optical absorption spectra of the electrochemically exfoliated transition metal dichalcogenides. a** UV-vis spectra platinum diselenide ($PtSe_2$) **b** tantalum disulfide (TaS_2) **c** $MoSe_2$ doped with Nb, and **d** WS_2 doped with Nb. The extinction, absorption and scattering components of the UV-vis spectra are shown in black, brown and blue for each material, respectively.

In Supplementary Figure 17a the spectra for $PtSe_2$ exhibit a broad absorption range with two peaks around 616 nm and 398 nm, respectively. The greater intensity of the 616 nm peak implies that the nanosheet is thin <5 nm (consistent with our AFM) and consistent with the peaks expected from the literature.[69] The absorption is high ~50% of the extinction signal at IR wavelengths, which would suggest that there are thicker nanosheets in the dispersion, which are semi-metallic rather than semiconducting like $PtSe_2$ monolayers.[69] The spectra of TaS_2 show absorption peak positions at 390 nm and 626 nm, which is consistent with a previous report with LPE nanosheets for UV wavelengths.[70] There is almost no scattering background at wavelengths above 650 nm, and there is strong absorption at wavelengths >600 nm, the lack of an absorption band edge would imply metallic behaviour in the material. There are no previous reports on the optical properties of the Nb-doped WSe_2 and $MoSe_2$ materials shown in Supplementary Figure 17c-d. But the peak positions and optical properties are the same as their undoped counterparts.

Supplementary Note 11 | UV-Vis: Post Transition Metal Dichalcogenide

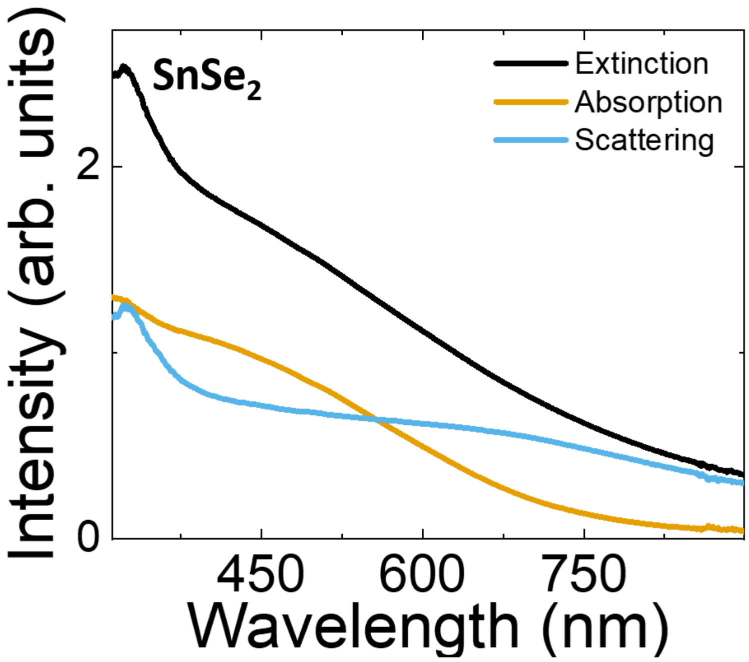

Supplementary Figure 18: **UV-visible optical absorption spectra of the electrochemically exfoliated post-TMD. a** UV-vis spectra of tin diselenide ($SnSe_2$) with the characteristic absorption peaks shown.

The UV-vis spectra for the post-TMD $SnSe_2$ is shown in Supplementary Figure 18. The spectra are featureless and is consistent with previous reports.[71] The high intensity in the IR wavelengths would imply metallic behaviour. All of the TMDs have strong absorption at shorter wavelengths (430 nm) due to interband transitions.[72]

Supplementary Note 12| UV-Vis: Transition Metal Trichalcogenides

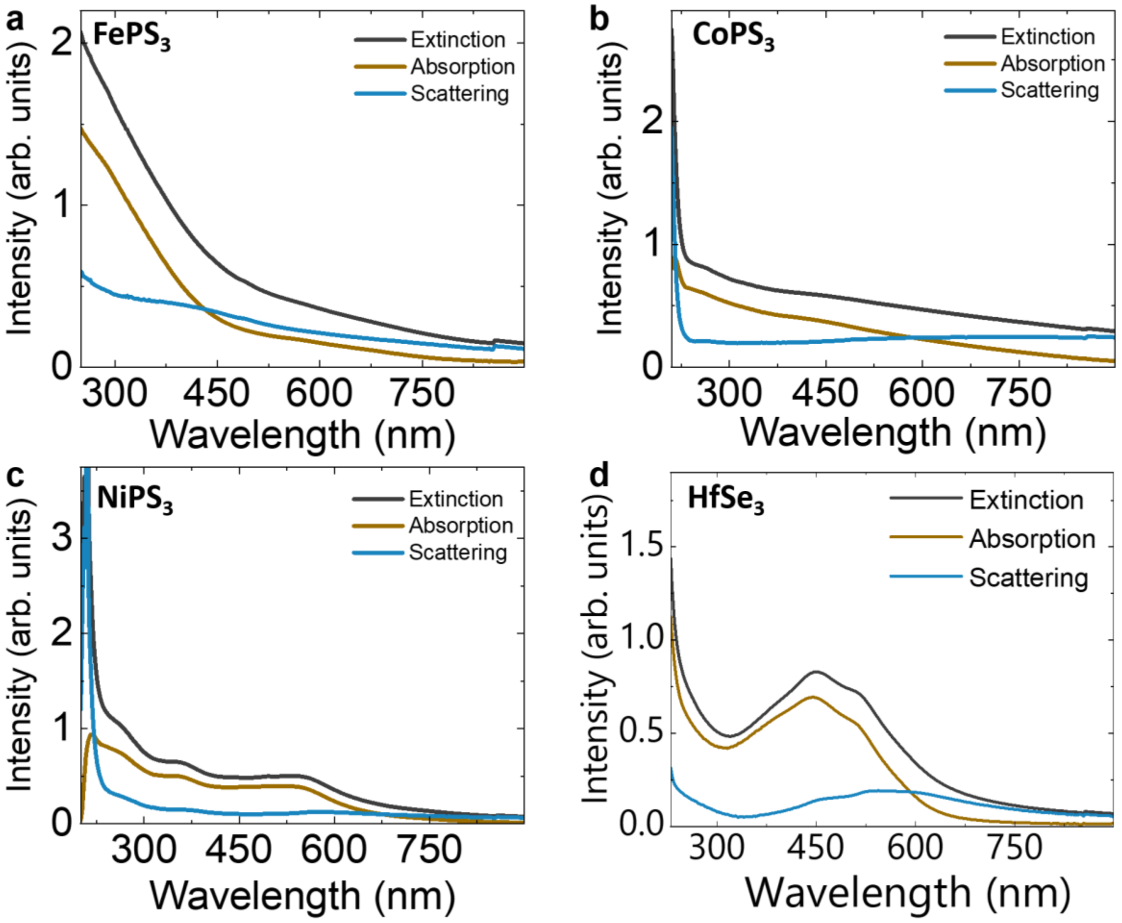

Supplementary Figure 19: **UV-visible optical absorption spectra of the electrochemically exfoliated TMTs. a** UV-vis spectra of iron phosphorus trichalcogenide ($FePS_3$), **b** cobalt phosphorus trichalcogenide ($CoPS_3$), **c** nickel phosphorus trichalcogenide ($NiPS_3$) and **d** hafnium triselenide ($HfSe_3$) with the characteristic absorption peaks shown.

Supplementary Figure 19 shows the extinction, absorption and scattering components of UV-visible optical absorption spectra for the TMTs. Iron phosphorus trichalcogenide ($FePS_3$) shows no prominent absorption peaks and stronger absorption at wavelengths <450 nm, consistent with a previous study.[73] Similarly, the cobalt phosphorus trichalcogenide ($CoPS_3$) spectra, shown in Supplementary Figure 19b, are mostly featureless, with a weak absorption peak found at ~ 216 nm. To our knowledge there are no other reports on this material reporting its optical properties. The spectra for nickel phosphorus trichalcogenide ($NiPS_3$) is shown in Supplementary Figure 19c and show characteristic absorption peaks at 218 nm, 352 nm, and 528 nm, consistent with a previous report.[74] In Supplementary Figure 19d we obtain the spectra of hafnium triselenide ($HfSe_3$) and observe absorption peaks at 444 and 514 nm. To our knowledge, the optical properties of $HfSe_3$ nanosheets have not previously been examined. However, it is possible that the peaks are related to the excitation of the charge carriers in the Se p-orbital valence states to the conduction band.[75]

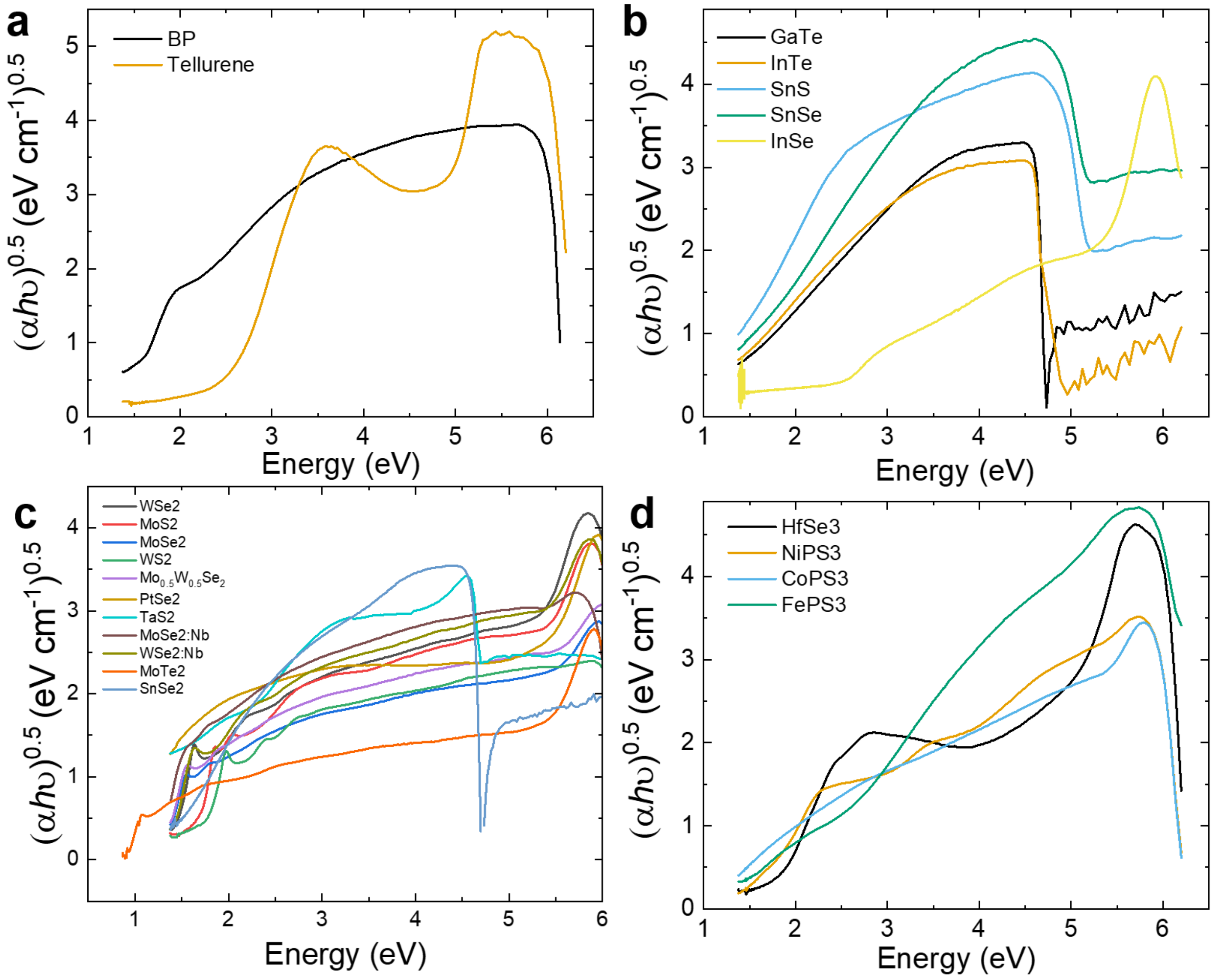

Supplementary Figure 20: **Tauc plots. a** Tauc plot of the elemental inks. **b** Tauc plot of the TMM inks. **c** Tauc plot of the TMD inks. **d** Tauc plot of the TMT inks.

We find the optical bandgap for each of the 2D inks in Supplementary Figure 20 with Tauc plots. By using the absorption data from UV-vis spectra, the optical bandgap (E_{Op}) of the semiconductor can be calculated by extrapolating a straight line of the $(\alpha h\nu)^{0.5}$ at the absorption band edge and assuming that we have indirect transitions.[76] The E_{Op} will be slightly less than the semiconductor bandgap as it doesn't account for the excitonic band gap[77] however it provides a good estimate for comparative study.

Supplementary Note 14 | Selection of Nanosheets for Each 2D Material Family

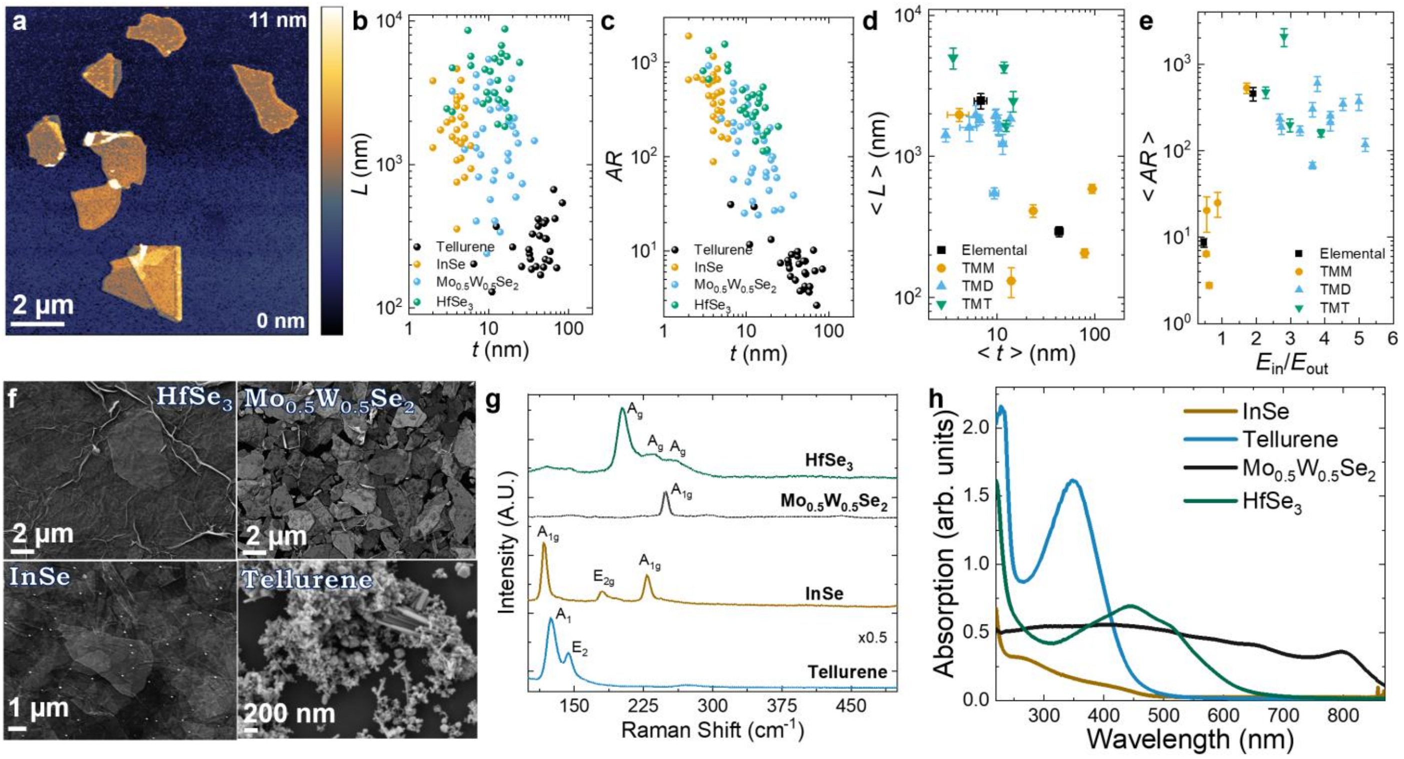

Supplementary Figure 21*:* **a** AFM micrograph showing InSe nanosheets drop-cast onto a Si/SiO_2 substrate. **b-c** Nanosheet lateral size , *L*, (**b**) and aspect ratio, AR, (**c**) plotted versus apparent nanosheet thickness , *t*, for four selected nanosheet types. Each data point represents a single nanosheet. **d** Relationship between average nanosheet lateral size (<*L*>) and apparent thickness (<*t*>) for each material under study. The data has been grouped into families of materials: TMT, TMD, TMM, and elemental materials. **e** Dependence of mean nanosheet AR for each material on the starting crystal's E_{in}/E_{out} ratio. The data has been grouped into material families, as shown in **d**. **f** Scanning electron microscopy images showing the morphology of solution-deposited nanosheet networks prepared using nanosheets from each material family. These images show alignment and connectivity for high *AR* nanosheets and the presence of nanoparticles and nanorods in the tellurene network. **g** Raman spectra of selected 2D materials showing characteristic vibrational modes, indicating the phase and quality of exfoliated nanosheets. **h** UV-visible optical absorption spectra of tellurene, InSe, $Mo_{0.5}W_{0.5}Se_2$, and $HfSe_3$ nanosheets, revealing their optical properties and excitonic peaks.

Atomic force microscopy (AFM) is utilised to determine the lateral size, *L* and observed nanosheet apparent thickness, *t* of nanosheets in the inks made from elemental, TMM, TMD and TMT materials. In Supplementary Figure 21, we improve the clarity of the presented data by presenting one material from each family studied, tellurene, InSe, $Mo_{0.5}W_{0.5}Se_2$ and $HfSe_3$. An AFM micrograph showing InSe nanosheets that are drop-cast onto a Si/SiO_2 substrate are shown in Supplementary Figure 21a, displaying average nanosheet lateral size, < *L* > ~ 2.0 ± 0.2 μm and average nanosheet apparent thickness, < *t* > ~ 4.0 ± 0.2 nm.

Supplementary Figure 21b illustrates the relationship between $< L >$ and $< t >$ for tellurene, InSe, $Mo_{0.5}W_{0.5}Se_2$ and $HfSe_3$ nanosheets, showing no direct link, a contrast to what is observed for nanosheets produced via liquid phase exfoliation (LPE), where $< L >$ will increase with $< t >$.[8] The $< L >$ are measured to be 0.29 ± 0.02 µm for tellurene, 2 ± 0.2 µm for InSe, 1.9 ± 0.2 µm for $Mo_{0.5}W_{0.5}Se_2$ and 0.67 ± 0.05 µm for $HfSe_3$. Meanwhile, $< t >$ is found to be 43 ± 4 nm for tellurene, 4.0 ± 0.2 nm for InSe, 14 ± 1 nm for $Mo_{0.5}W_{0.5}Se_2$ and 12 ± 1 nm for $HfSe_3$. When the *AR*, ($L\ t^{-1}$) is plotted against *t*, as shown in Supplementary Figure 21c, the maximum *AR* values recorded were ~ 1000 for InSe, $Mo_{0.5}W_{0.5}Se_2$ and $HfSe_3$ and *AR* < 100 for tellurene. To enable the highest possible device performances an *AR* > 40 is considered necessary to facilitate nanosheet-to-nanosheet connections with minimised R_J.[78] Therefore InSe, $Mo_{0.5}W_{0.5}Se_2$ and $HfSe_3$ will make nice conformal networks once deposited. LS deposition can help to align the nanosheets[79] however, once the AR > 40, the nanosheets self-align their basal plane parallel to the substrate, even if the inks are drop-cast.[80] Supplementary Figure 21d summarises the $< L >$ and $< t >$ found for each 2D nanosheet ink. For each material family, $< L >$ is not strongly correlated to $< t >$, which is unique to EE nanosheet production. The trend is unexpected as traditional LPE exfoliation techniques such as ultrasonication, shear mixing or microluidization would show a strong correlation of increasing $< L >$ with $< t >$, which can be attributed to the interplay of matching hasan solubility parameters and the mechanical action of the exfoliation process which favours larger and thicker nanosheets.[81] In contrast, the EE is producing nanosheets where $< L >$ is not increasing with $< t >$, likely due to the nature of the EE exfoliation mechanism where once ion intercalation overcomes E_b, exfoliation is likely, and then the resulting nanosheet geometry is largely dependet on the crystal mechanical properties and post-expansion size selection protocols rather than the mechanical or chemical force applied. In Supplementary Figure 21e, we show how $< AR >$ depends on the starting crystals E_{in}/E_{out} for each material family. It is observed that once the E_{in}/E_{out} is > 1.7, the nanosheet AR > 500, which is independent of the family of 2D material. Furthermore, if E_{in}/E_{out} <1, the AR is less than 25, which is not a sufficient *AR* to make conformal nanosheet-to-nanosheet junctions for high-performance devices.[78]

In Supplementary Figure 21f, we examine a selection of networks from each material family by scanning electron microscopy (SEM) to assess the nanosheets' morphology when assembled in a network. In materials such as InSe, MoWSe and $HfSe_3$, where the nanosheet AR is high (>100), the network reveals well-aligned and seamless connections between nanosheets, suggesting low R_J.[78] Additionally, the presence of folds and wrinkles in the nanosheets indicates a high degree of nanosheet flexibility, which is expected since our AFM results indicate low apparent thickness (<10 nm) in these materials. In Figure 2f, we observe a nonconformal and misaligned tellurene network. We attribute the misalignment of the nanosheets to their low aspect ratio (<40) since the network has a similar morphology to what is typically seen in low *AR* LPE networks.[78] Rods are also observed in the network previously observed in solution phase synthesis of tellurene.[82] The co-existence of tellurene nanowires with nanosheets has previously been observed during the growth of tellurene nanosheets. The formation of tellurene nanowires is energetically preferred for the Te atoms. It is possible that

the electrochemical exfoliation of the bulk telluride provides a combination of both 1D and 2D dimensionalities.[83]

We use Raman spectroscopy with a 532 nm laser to identify the phase and quality of the 2D nanosheets after exfoliation. Supplementary Figure 21g depicts select spectra from each material family being examined. Tellurene nanosheets (blue curve) show A_1 and E_2 modes at 124 and 144 cm^{-1}.[52] The presence of the E_2 mode would suggest that the nanosheets are >20 nm thick (consistent with our AFM results) which is not active for monolayer or few-layer nanosheets.[53] For few-layer InSe (brown curve), the A_{1g}, E_{2g}, and another A_{1g} vibrational modes are identified at ~ 117, 180, and 228 cm^{-1}, associated with out-of-plane and in-plane vibrations of Se and In atoms, respectively.[19] All TMDs studied are consistent with previous reports of 2H semiconducting nanosheets since the J_1, J_2 and J_3 vibrational modes attributed to the metallic 1T phase are not observed.[36,37,39] The TMD alloy of $Mo_{0.5}W_{0.5}Se_2$ is shown in Supplementary Figure 21g (black curve) and we find the A_{1g} vibrational mode at ~ 247 cm^{-1} attributed to the out-of-plane motion of the Se atoms relative to a Mo or W atom and a weak E_{2g} vibration at ~ 146 cm^{-1} attributed to the in-plane vibrational motion of the Se atoms.[32] $HfSe_3$ (green curve) shows a A_g peaks at 202 cm^{-1}, 232 cm^{-1} and 253 cm^{-1}, which are likely attributed to the A_g vibrational mode.[54] Supplementary Figure 21h presents a selection of the UV-visible optical absorption for tellurene, InSe, $Mo_{0.5}W_{0.5}Se_2$ and $HfSe_3$ inks, obtained using an integrating sphere where we have subtracted the scattering component of each spectrum from the extinction spectra.[58] The spectra of tellurene (blue curve) reveal an excitonic transition at 348 nm attributed to the transition of valence band p-orbital charge carriers to the conduction band.[60] The peak position is consistent with predicted density functional theory (DFT) values.[60,84] The spectra of InSe (brown curve) show a broad peak at ~260 nm, consistent with the expected peak position from DFT calculations.[61] The $Mo_{0.5}W_{0.5}Se_2$ spectra (black curve) show excitonic peaks similar to $MoSe_2$ (Supplementary Figure 16c) corresponding to the A and B excitons at 798 nm and 658 nm, respectively.[66] The spectra of $HfSe_3$ is shown as the green curve and show absorption peaks at 444 and 514 nm. To our knowledge, the optical properties of $HfSe_3$ nanosheets have not previously been examined. However the peaks may be related to the excitation of the charge carriers in the Se p-orbital valence states to the conduction band.[75] The UV-vis of all other inks are detailed in Supplementary Note 8-10. We estimate the inks' optical bandgap (E_{Op}) by plotting Tauc plots with absorption spectra (Supplementary Note 13). A straight line is extrapolated to the x-axis intercept to estimate E_{Op}. The E_{Op} is between 0.8 – 2.1 eV for most 2D inks except for TaS_2, where the intercept is negative, likely due to the metallic properties of the material, therefore we assume E_{Op} = 0 eV since the E_f is in the conduction band. 2H-TaS_2 has previously shown metallic-like behaviour when electrochemically intercalated with ammonium cations[85] and the E_F is found in the conduction band.[86]

Supplementary Note 15 | Effect of Interlayer Spacing on Crystal Expansion

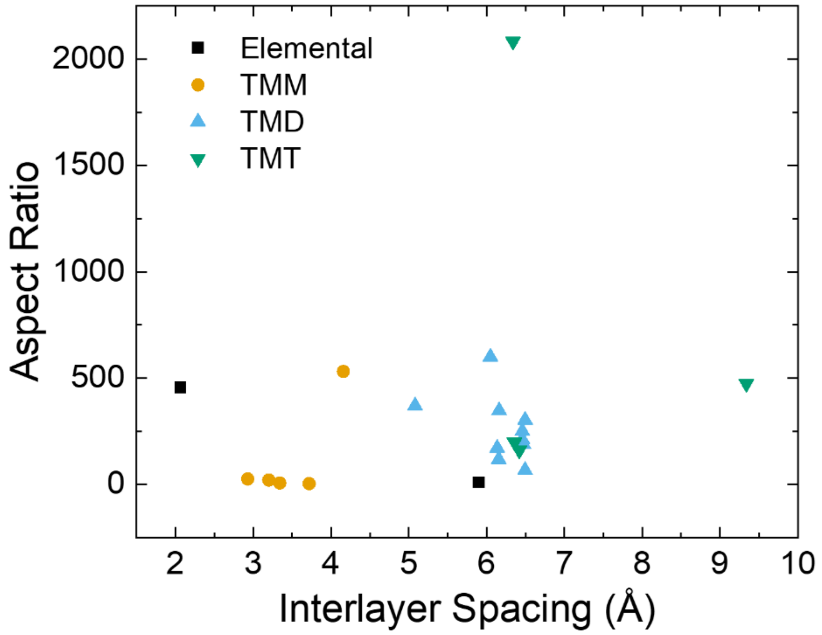

Supplementary Figure 22: The elemental (black), TMM (yellow), TMD (blue) and TMT (green) crystals are plotted as a function of their aspect ratio after exfoliation and their interlayer spacing. We use interlayer spacings found from the XRD library from the International Centre for Diffraction Data.

We investigate the effect of the crystal interlayer spacing on the flake aspect ratio and find no correlation between the interlayer spacing and the aspect ratio of the flakes exfoliated shown in **Supplementary Figure 22** across the different families of 2D materials presented in Supplementary **Table 3**. We keep the intercalating ion diameter constant ($TPA^+ \approx 9.5$ Å) in all intercalations so that the mechanical properties could be changed and the aspect ratio measured. Materials such as $HfSe_3$ (interlayer spacing, d = 9.34 Å) and black phosphorous (d = 2.06 Å) have varied interlayer spacing but in both cases the flakes have large aspect ratio >> 30. Furthermore, materials with large interlayer spacing such as InTe or GaTe (3-4 Å) do not exfoliate well with AR < 30. Therefore, interlayer spacing is not a good metric for quantifying exfoliation success. We also find the interlayer spacing for each crystal is less than the TPA^+ cation diameter (≈ 9.5 Å) indicating that the cation must either elastically deform to enter the crystals or apply force to the outside of the crystals through electrostatic repulsion from other cations.

Material	<*AR*>	Interlayer Spacing (Å)	E_{in}/E_{out}
BP	456 ± 81	2.064	1.9
Tellurene	9 ± 1	5.9	0.47
GaTe	3 ± 1	3.72	0.62
InSe	531 ± 74	4.16	1.72
InTe	6 ± 1	3.342	0.54
SnS	20 ± 9	3.2	0.55
SnSe	25 ± 8	2.93	0.87
$MoSe_2$	250 ± 35	6.46	4.16
$MoSe_2$:Nb	212 ± 43	6.46	4.16
WSe_2	67 ± 6	6.494	3.64
WSe_2:Nb	303 ± 57	6.494	3.64
MoS_2	117 ± 20	6.155	5.18
$Mo_{0.5}W_{0.5}Se_2$	188 ± 33	6.475	2.73
$PtSe_2$	370 ± 78	5.08	5
WS_2	347 ± 55	6.161	4.53
$SnSe_2$	171 ± 21	6.137	3.27
TaS_2	600 ± 115	6.05	3.78
$NiPS_3$	2084 ± 448	6.34	2.81
$CoPS_3$	198 ± 33	6.36	2.98
$FePS_3$	160 ± 16	6.422	3.89
$HfSe_3$	473 ± 72	9.34	2.81

Supplementary Table 3: Table of nanosheet aspect ratio, interlayer spacting and E_{in}/E_{out} values used.

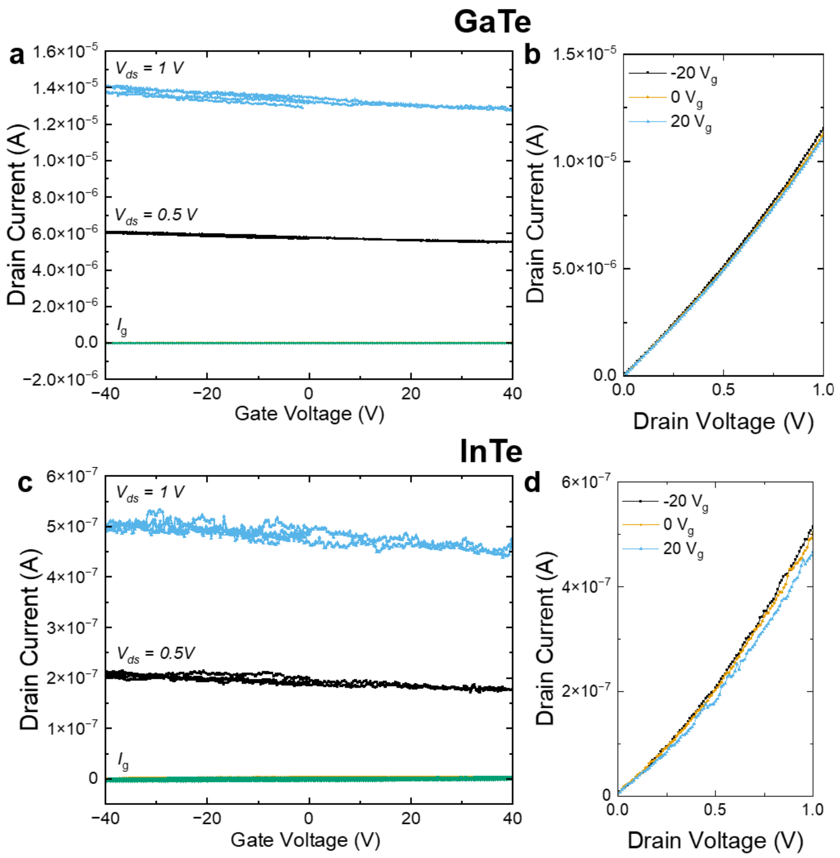

Supplementary Figure 23**:** Transfer characteristics of GaTe and InTe. **a** Gate voltage sweep on GaTe nanosheet network with V_{ds} = 0.5 and 1 V. Gate leakage, I_g is shown as the green line. **b** Output characteristic of the GaTe device. **c** Gate voltage sweep on InTe nanosheet network with V_{ds} = 0.5 and 1 V. **d** Output characteristic of the InTe device.

We make solid-state devices using pre-patterned gold electrodes on Si/SiO_2 (90 nm SiO_2) (Fraunhofer Gen 4 OFET chips) which have much smaller L_{CH} = 2.5 µm (W_{CH} = 10 mm) than can be achieved with shadow mask evaporation, ideal for probing small L <500 nm nanosheets. We drop cast GaTe and InTe into the substrates after EE in a glovebox in N_2. Exfoliation, centrifugation and washing protocols are the same as described in the main text. The nanosheets are of poor quality due to their low $E_{in}/E_{out} < 1$ and, therefore have low AR = 3-6, which is insufficiently high to create conformal nanosheet-to-nanosheet junctions. In Supplementary Figure 23, we plot the transfer characteristics for each material. Both InTe and GaTe show weak p-type behaviour which has not yet been reported for networks of solution processed nanosheets. The GaTe p-type behaviour has previously been reported for mechanical exfoliation of GaTe[87] and to our knowledge InTe transistors have not previously been reported. The average ambient μ_{NET} for the GaTe networks is $\mu_{GaTe} \approx 3.8 \times 10^{-7} \pm 7.6 \times 10^{-8}$ cm^2 V^{-1} s^{-1} (N = 4) and for InTe is $\mu_{InTe} \approx 1.7 \times 10^{-8} \pm 7.0 \times 10^{-9}$ cm^2 V^{-1} s^{-1} (N = 4). In both InTe and GaTe $I_{on}/I_{off} < 2$. In both GaTe and InTe the I_G current is several magnitudes (≈ 3 nA) below I_D, therefore we believe it is real modulation of the semiconductor, despite the low μ_{Net} obtained.

Supplementary Note 17 | Network Capacitance of Ionic Liquid Filled Networks

The areal capacitance (C_A) of a nanosheet network filled with ionic liquid will depend on various factors, including the voltage scan rate as shown in our previous work.[67] For an EE MoS_2 network (with the same nanosheet manufacturing process used in this article) with a network thickness, $t_{NET,MOS2}$ = 25 nm and nanosheet thickness $t_{NS,MOS2} = 14\ nm$, and using a CV scan rate of 50 mV s^{-1}, we previously found C_A = 3.1 μF/cm^2. [67] However, C_A is dependent on both nanosheet thickness and network thickness because $C_A = C_V t_{Net}$ where C_V is the volumetric capacity of the network. In addition, it has been shown that $C_V \propto 1/t_{NS}$.[88] This is important here because, for our networks, t_{NET} varies between deposition methods. LS deposition can make ultra-thin networks < 20 nm however drop-casting often results in thicker films >50 nm due to poor control of the ink volume deposited. Thicker networks will inevitably have higher capacitance while thicker nanosheets will decrease C_A. In addition, our nanosheets have different thicknesses (t_{NS}) across materials. Therefore, we need to correct our C_A for each network using the information above which can be used to generate the equation,

$$C_A = C_{A,MOS2}\left(\frac{t_{NS,MOS2}}{t_{NS}}\right)\left(\frac{t_{NET}}{t_{NET,MOS2}}\right) \qquad (3)$$

Material	t_{NS} (nm)	t_{NET} (nm)	C_A (μF/cm^2)
BP	7	50	12.40
Tellurene	43	550	22.20
GaTe	79	450	9.89
InSe	4	40	17.36
InTe	95	320	5.85
SnS	14	250	31.00
SnSe	23	350	26.42
$MoSe_2$	10	28	4.86
$MoSe_2$:Nb	10	20	3.47
WSe_2	9	30	5.79
WSe_2:Nb	5	25	8.68
MoS_2	11	25	3.95
$Mo_{0.5}W_{0.5}Se_2$	14	20	2.48
$PtSe_2$	6	19	5.50
WS_2	7	45	11.16
$MoTe_2$	10	20	3.47
$SnSe_2$	10	28	4.86
TaS_2	3	50	28.93
$NiPS_3$	4	50	21.70
$CoPS_3$	15	170	19.67
$FePS_3$	12	30	4.34
$HfSe_3$	12	21	3.04

Supplementary **Table 4:** Table of nanosheet thickness, network thickness and network capacitance values.

We calculate μ_{NET} of the transistors from the equation $\mu_{NET} = (L_{CH} / W_{CH})(1/C_A)(g_m/V_{ds})$, where $g_m = \partial I_d/\partial V_g$ is the transconductance (e.g., measured from the slope of the transfer characteristic). μ_{peak} is found using the maximum value of the g_m. For the n-type and ambipolar materials the electron mobility is calculated and for the p-type materials the hole mobility is calculated.

We can compare our transistor μ_{NET} presented in Supplementary Table 5 to others in the literature for solution-processed networks. EE MoS_2 nanosheet networks have achieved $\mu_{Net} \sim 10$ cm^2 $V^{-1}s^{-1}$ and $I_{on}/I_{off} \sim 10^6$ when made on silicon/silicon oxide (Si/SiO_2) substrates.[89] Other 2D nanosheet inks have also been used to make FETs. We have previously shown that tungsten disulfide (WS_2) and tungsten diselenide (WSe_2) have demonstrated n-type and ambipolar behaviour, respectively, achieving performances up to $\mu_{Net} \sim 10$ cm^2 $V^{-1}s^{-1}$ and $I_{on}/I_{off} \sim 10^4$.[67] Platinum diselenide ($PtSe_2$) has demonstrated n-type behaviour on Si/SiO_2, achieving $\mu_{Net} \sim 0.004$ cm^2 $V^{-1}s^{-1}$, $I_{on}/I_{off} \sim 10^3$.[90] P-type behaviour has also been demonstrated with violet phosphorus (VP) achieving $\mu_{Net} \sim 2$ cm^2 $V^{-1}s^{-1}$ and $I_{on}/I_{off} \sim 10^4$.[91] EE BP field effect transistor (FET) with p-type behaviour have also been demonstrated on Si/SiO_2, achieving $\mu_{Net} \sim 0.002$ cm^2 $V^{-1}s^{-1}$ and $I_{on}/I_{off} \sim 10^2$.[92] Immersion of an EE WSe_2 network on Si/SiO_2 in iron trichloride ($FeCl_3$) and bromine (Br_2) solutions followed by annealing have been used to molecularly dope WSe_2 from its intrinsic ambipolar behaviour to a p-type nanosheet. The FET devices achieved performances of $\mu_{Net} \sim 1.5$ cm^2 $V^{-1}s^{-1}$, $I_{on}/I_{off} \sim 10^6$ and $\mu_{Net} \sim 27$ cm^2 $V^{-1}s^{-1}$, $I_{on}/I_{off} \sim 10^7$ respectively.[93,94] A further larger survey is conducted in the literature review of Supplementary Note 29. To our knowledge, we are the first to study the electrical properties of many of these materials with μ_{Net} comparable to state-of-the-art or reported for the first time.

Material	μ_{NET} (cm^2 V^{-1} s^{-1})	μ_{peak} (cm^2 V^{-1} s^{-1})	I_{on}/I_{off}	N	Comment
**BP**	**$0.0008 \pm 4.7\times10^{-5}$**	**$0.0020 \pm 4.0\times10^{-4}$**	76×10^{1}	12	
**Tellurene**	**0.0012 ± 0.0010**	**0.002 ± 0.001**	6×10^{2}	6	
**GaTe**	**$3.8\times10^{-7} \pm 7.6\times10^{-8}$**	-	< 2	4	
**InSe**	-	-	-	-	Too Insulating
**InTe**	**$1.7\times10^{-8} \pm 7.0\times10^{-9}$**	-	< 2	4	
**SnS**	-	-	-	-	Too Insulating
**SnSe**	-	-	-	-	Too Insulating
**$MoSe_2$**	2.9 ± 0.4	5.4 ± 0.7	2×10^{5}	6	
**$MoSe_2$:Nb**	0.8 ± 0.04	2.2 ± 0.23	8×10^{0}	8	
**WSe_2**	1.7 ± 0.2	2.6 ± 0.4	4×10^{4}	6	
**WSe_2:Nb**	2.0 ± 0.2	3.4 ± 0.2	3×10^{3}	7	
**MoS_2**	8.4 ± 0.7	11.6 ± 1.2	2×10^{3}	9	
**$Mo_{0.5}W_{0.5}Se_2$**	5.5 ± 0.9	13.2 ± 1.1	3×10^{5}	5	
**$PtSe_2$**	1.1 ± 0.1	8.1 ± 1.5	5×10^{2}	5	
**WS_2**	7.8 ± 0.8	13.3 ± 1.6	3×10^{5}	3	
**$MoTe_2$**	2.3 ± 0.6	3.1 ± 0.7	7×10^{4}	7	
**$SnSe_2$**	-	-	-	-	Metallic
**TaS_2**	-	-	-	-	Metallic
**$NiPS_3$**	-	-	-	-	Too Insulating
**$CoPS_3$**	-	-	-	-	Too Insulating
**$FePS_3$**	-	-	-	-	Too Insulating
**$HfSe_3$**	-	-	-	-	Too Insulating

Supplementary **Table 5:** Table of network mobility, peak mobility, on/off ratio and sample amount of each ionicly gated network.

Supplementary Note 18 | Ionic Current of Liquid Gated Transistors

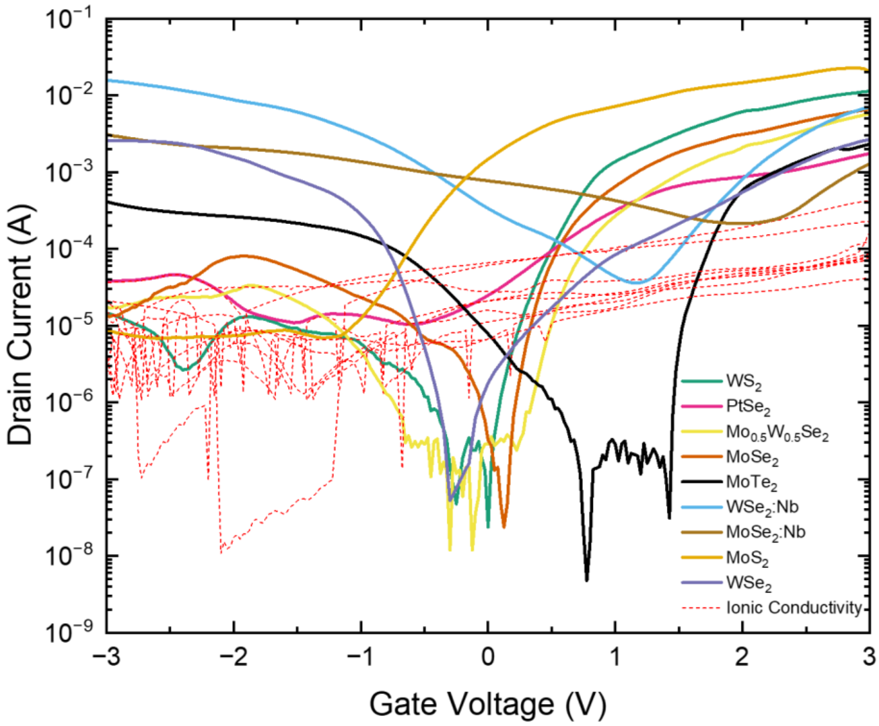

Supplementary Figure 24: Transfer curve of each nanosheet network used to make ionically gated transistors. Ionic current (dashed red line) of each ionically gated transistor, which is consistent with the conductivity of EMIM-TFSI found in previous work.[67]

Supplementary Note 19 | Output Characteristics of Ionic Gated Transistors

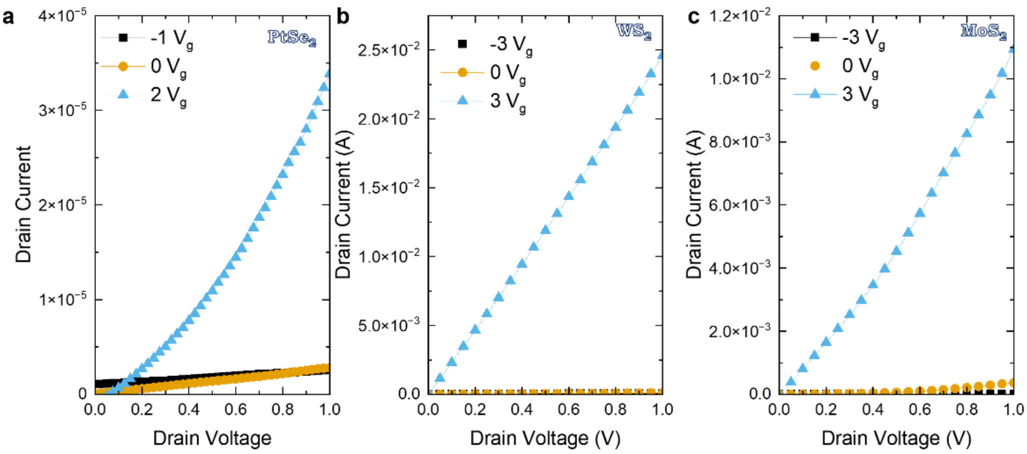

Supplementary Figure 25: Output curves of the n-type ionic gated transistors showing the highest drain current at positive gate voltages. We attribute the current offset at 0 V to the ionic liquid current.

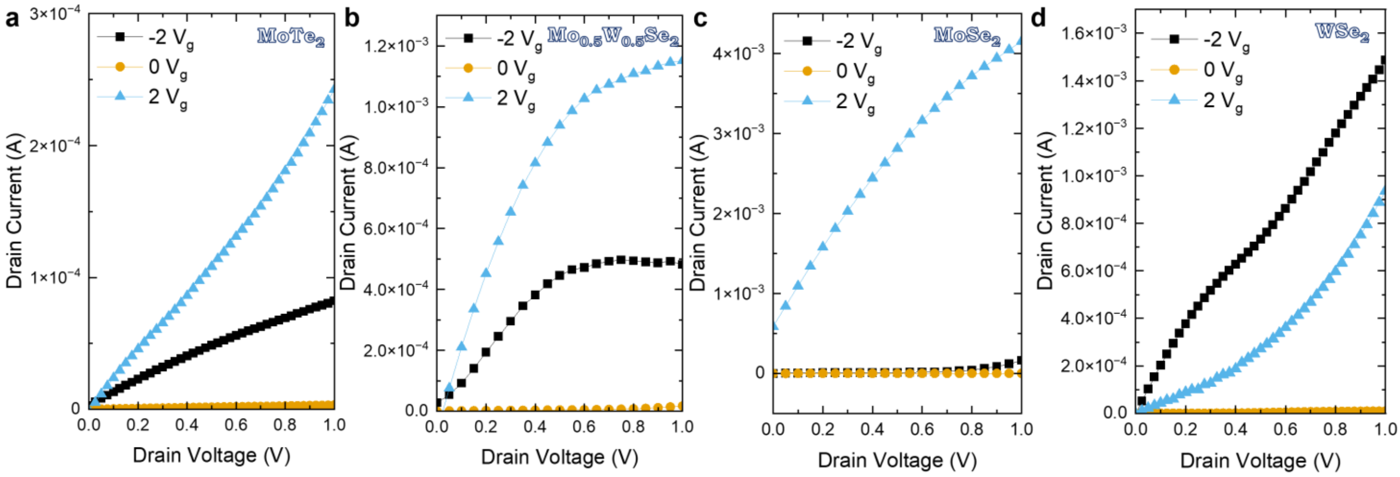

Supplementary Figure 26: Output curves of the ambipolar ionic gated transistors showing the highest drain current at positive and negative gate voltages. We attribute the current offset at 0 V to the ionic liquid current.

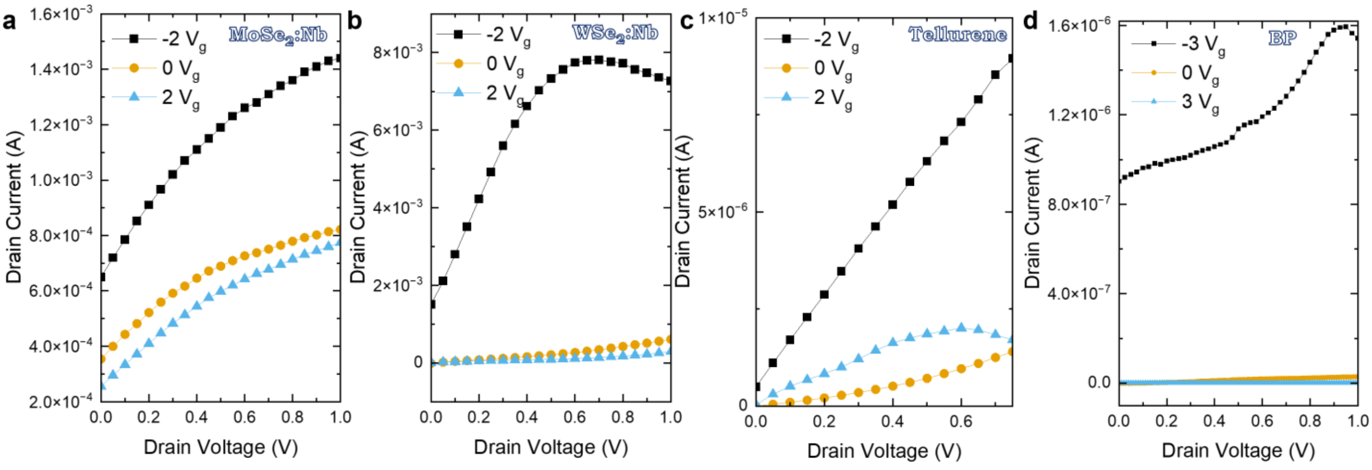

Supplementary Figure 27: Output curves of the p-type gated transistors showing the highest drain current at negative gate voltages. We attribute the current offset at 0 V to the ionic liquid current.

Supplementary Note 20 | Further Examination of Network Charge Carrier Densities

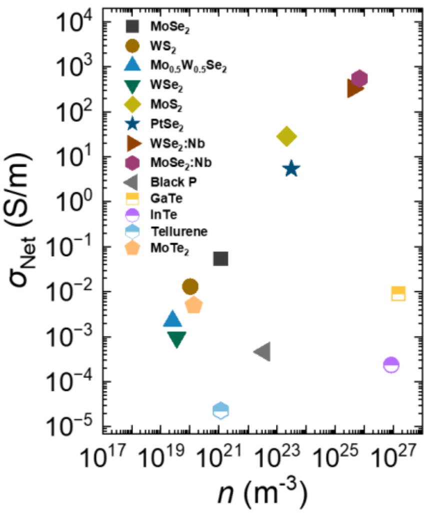

Supplementary Figure 28: Summary of the network conductivity (σ_{Net}) and charge carrier density (n) for each nanosheet network.

The network conductivity of our nanosheet networks is directly proportional to the number of charge carriers available to transport electric charge, n which can be understood through $\sigma_{Net} = nq\mu_{Net}$, where q is the charge of the carriers. Since the network μ_{NET} is now known from our transistors, we can calculate n for each network, assuming μ_{NET} is independent of n, as shown in Supplementary Figure 28. We observe an increase in n with σ_{NET} since more charge carriers are available to conduct current. Our niobium-doped WSe_2 and $MoSe_2$ have the highest $n \sim 10^{24}$ - 10^{27} m^{-3} as expected and similar in magnitude to previous mechanically exfoliated niobium-doped TMDs.[95] As a consequence of their high n, niobium-doped WSe_2 and $MoSe_2$ devices suffer from charge carrier screening of the gate's electric field and suffer from low I_{on}/I_{off} ratio ~10^2. $PtSe_2$ and MoS_2 are unlike the other EE semiconductors since they have a high $n \sim 10^{23}$ m^{-3}. MoS_2 is the only natural 2D crystal we have used for the article, and it is likely that the crystal is unintentionally doped with rhenium before it is mined.[96] Even in dilute concentrations (<1 at.%) it can drastically n-dope the MoS_2 and increase n.[97] A high n would explain why I_{off} is high (~ 1 µA) even in literature solid-state devices, resulting in a low I_{on}/I_{off}. Torsi *et. al* have also observed that V_{th} is shifted towards negative voltages[97] with Re doping, which is widely seen for the majority of solution-processed literature (Supplementary Note 29). $PtSe_2$ electrical properties are highly dependent on its layer thickness, it is a semiconductor up to 3 atomic layers but a semimetal for >3 layers ($t > 5$ nm).[98] We find $< t > = 6 \pm 1$ nm for our EE ink (Supplementary Note 3), which is a composite of thick (>10 nm) and thin (1 nm) nanosheets. Therefore, we have a combination of semiconducting and semi-metallic properties, resulting in a high n. We find that the elemental materials (BP and tellurene) are also outliers in Supplementary Figure 28, possibly due to an underestimation of their mobility. Tellurene is a low *AR* material (*AR* = 9) (Supplementary Note 3), while BP has a puckered structure forbidding the formation of conformal junctions.[78,99] Additionally, BP and tellurene are not air stable,[100,101] therefore is it possible that oxidation reduced the effective μ_{NET} in the devices.

1) OPTP measurements

We obtain an approximation of the real THz conductivity averaged over the frequency with OPTP measurements. The sum of the products of the quantum yields of electrons and holes and their respective (real) mobilities are obtained according to

$$S_{approx}(\tau) = \Phi_e(\tau)\mu_e + \Phi_h(\tau)\mu_h = \frac{\mathcal{E}_0 c(n_f + n_b)}{eN_a}\left[\frac{E^{off}(t_{max}) - E^{on}(t_{max},\tau)}{E^{on}(t_{max},\tau)}\right]. \quad (4)$$

Here, τ is the delay time between the optical pump pulse and the time at which the THz conductivity is determined, N_a is photoexcitation density per unit area (2×10^{12} - 8×10^{12} photons cm^{-2}), $\mathcal{E}_0$ is the vacuum permittivity, c is the speed of light, while n_f and n_b are the refractive indices of the media in front and back of the sample, respectively. The samples are photoexcited with pump photon energy of 3.1 eV, which is well above the bandgap of the TMDs studied and thus leads to initially hot charge carriers. The hot charges can energetically relax to the band edges by phonon emission and decay via recombination to neutral excitons or entrapment at defects. We assume that the energetic relaxation to the band edges occurs within 5 ps.

2) TRTS measurements

TRTS is a more accurate measurement than OPTP and provides the sum of the products of the quantum yields of electrons and holes and their respective (real) mobilities as a function of the radian probe frequency, ω. We measure the THz wave forms $E^{off}(t)$ and $E^{on}(t,\tau)$ as a function of the THz delay time, t, and calculate their Fourier transforms to obtain the frequency dependent results according to

$$S(\omega,\tau) = \Phi_e(\tau)\mu_e(\omega) + \Phi_h(\tau)\mu_h(\omega) = \frac{c\mathcal{E}_0(n_f + n_b)}{eN_a}\frac{E^{off}(\omega,\tau) - E^{on}(\omega,\tau)}{E^{on}(\omega,\tau)}. \quad (5)$$

. Figure 4a of the main text shows the sum of the products of the quantum yields of electrons and holes and their respective mobilities as a function of frequency, as obtained from TRTS measurements and eq. 5. In the main text we define the nanosheet mobility as $\mu_{NS}(\omega,\tau) = S(\omega,\tau)$. The μ_{NS} at 1 THz and τ = 5 ps is found to be 75, 61, 51, 42, 38, 32, 26 for WSe_2, WS_2, $MoSe_2$, MoS_2, $Mo_{0.5}W_{0.5}Se_2$, WSe_2:Nb and $MoSe_2$:Nb, respectively. For the $PtSe_2$ sample the OPTP measurements showed a rapid decay within a few picoseconds, therefore we determined the nanosheet mobility from TRTS at τ = 2 ps, which is equal to 18 $cm^2V^{-1}s^{-1}$.

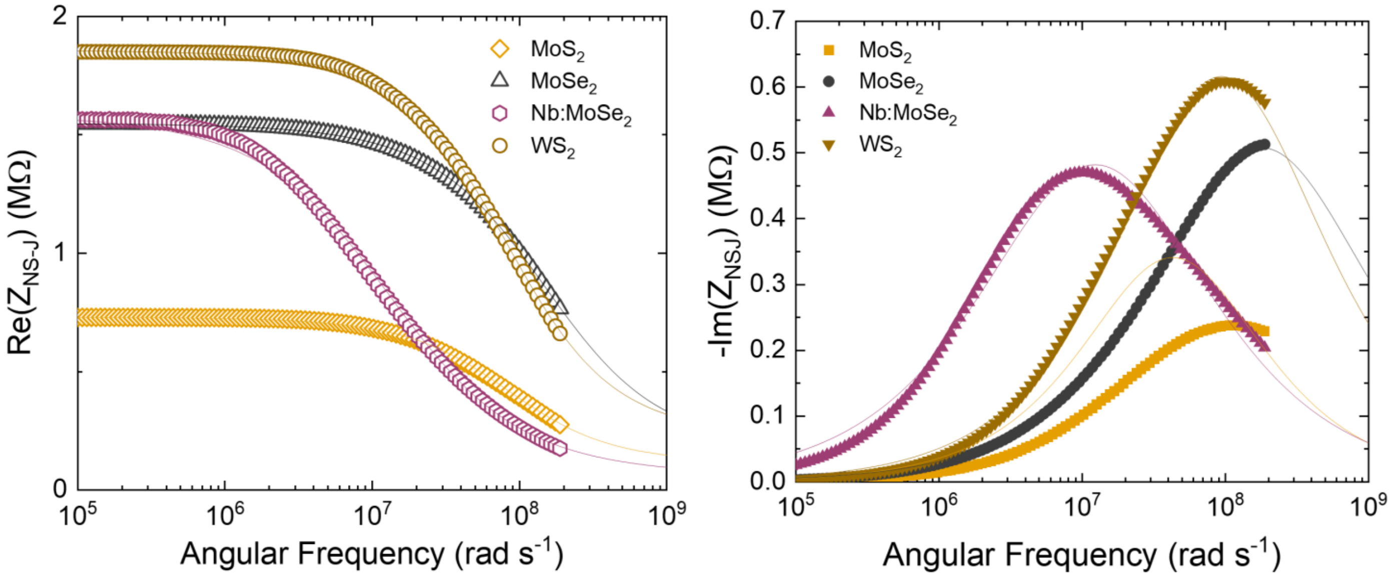

Supplementary Figure 29: Real (left) and imaginary (right) part of the impedance of a nanosheet junction pair, measured for different networks. The curves are fitted using a modified equation for the Randles circuit presented in our previous work.[102]

We have recently demonstrated that A.C. impedance spectroscopy allows R_J, R_{NS}, to be measured simultaneously with μ_{NS} and μ_{NET} indirectly estimated by leveraging the capacitance of the junctions between nanosheets.[102] The network impedance, Z_{Net}, is converted into the impedance of the average nanosheet-junction pair, $Z_{NS\text{-}J}$. The $Z_{NS\text{-}J}$ spectrum is then fitted with an equivalent circuit model of a Randle's circuit to yield values of R_{NS}, R_J for networks of MoS_2, WS_2, $MoSe_2$ and $MoSe_2$:Nb. The real component of $Z_{NS\text{-}J}$ is shown in Supplementary Figure 29 (left) while the imaginary component of $Z_{NS\text{-}J}$ is shown on the right. At low frequency, the real impedance, Re($Z_{NS\text{-}J}$) is equal to $R_j + R_{NS}$ and as the frequency approaches infinity, the impedance will equal R_{NS}.

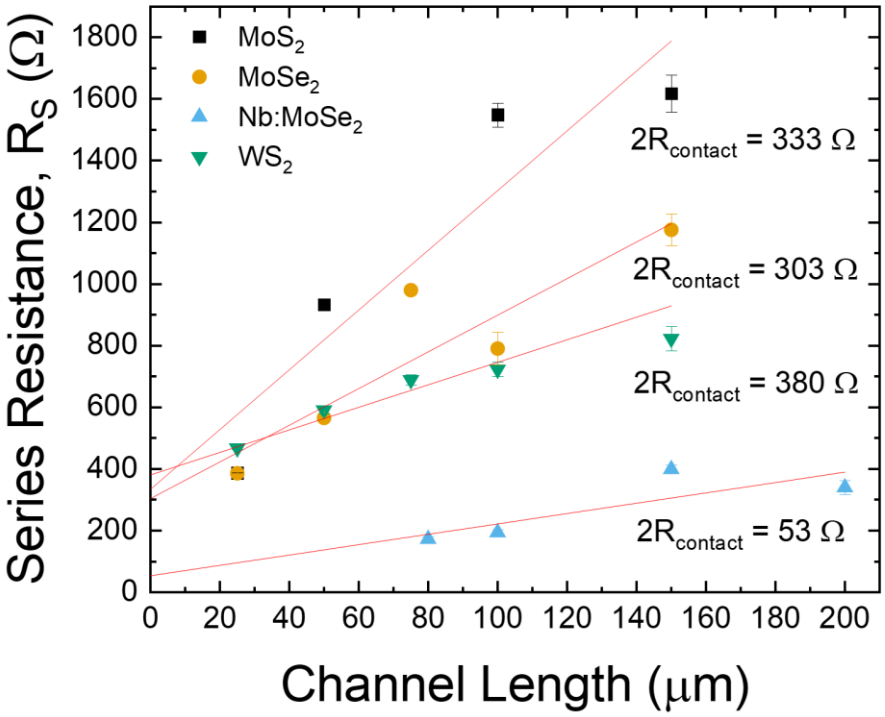

Supplementary Figure 30: Measurement of contact resistance. Series resistance Rs as a function of the channel length on networks of MoS_2, $MoSe_2$, $MoSe_2$:Nb and WS_2.

We use impedance spectroscopy to extract the series resistance R_S our equivalent circuit model of a Randel's circuit, since the contact resistance, R_c will appear as a contribution to the series resistance. We follow the protocol established in our previous work[102] to extract R_S and R_J and then plot R_S vs channel length in Supplementary Figure 30 for MoS_2, $MoSe_2$, $MoSe_2$:Nb and WS_2 networks. We find the percentage change of the R_S component (ΔR_S) between the high (30Mhz) and low (20 Hz) frequency regimes for each channel length. For MoS_2, WS_2 and $MoSe_2$ the ΔR_S ~ 7-50%, while for $MoSe_2$:Nb the ΔR_S ~ 80%. The intercept is then $2R_C$ as in the case of the transfer length method to measure contact resistance. The channel width in each case is 19.4 mm. Therefore, we find the contact resistance is 3.2 MΩ μm, 2.9 MΩ μm, 3.7 MΩ μm and 1.0 MΩ μm for MoS_2, $MoSe_2$, WS_2 and $MoSe_2$:Nb respectively.

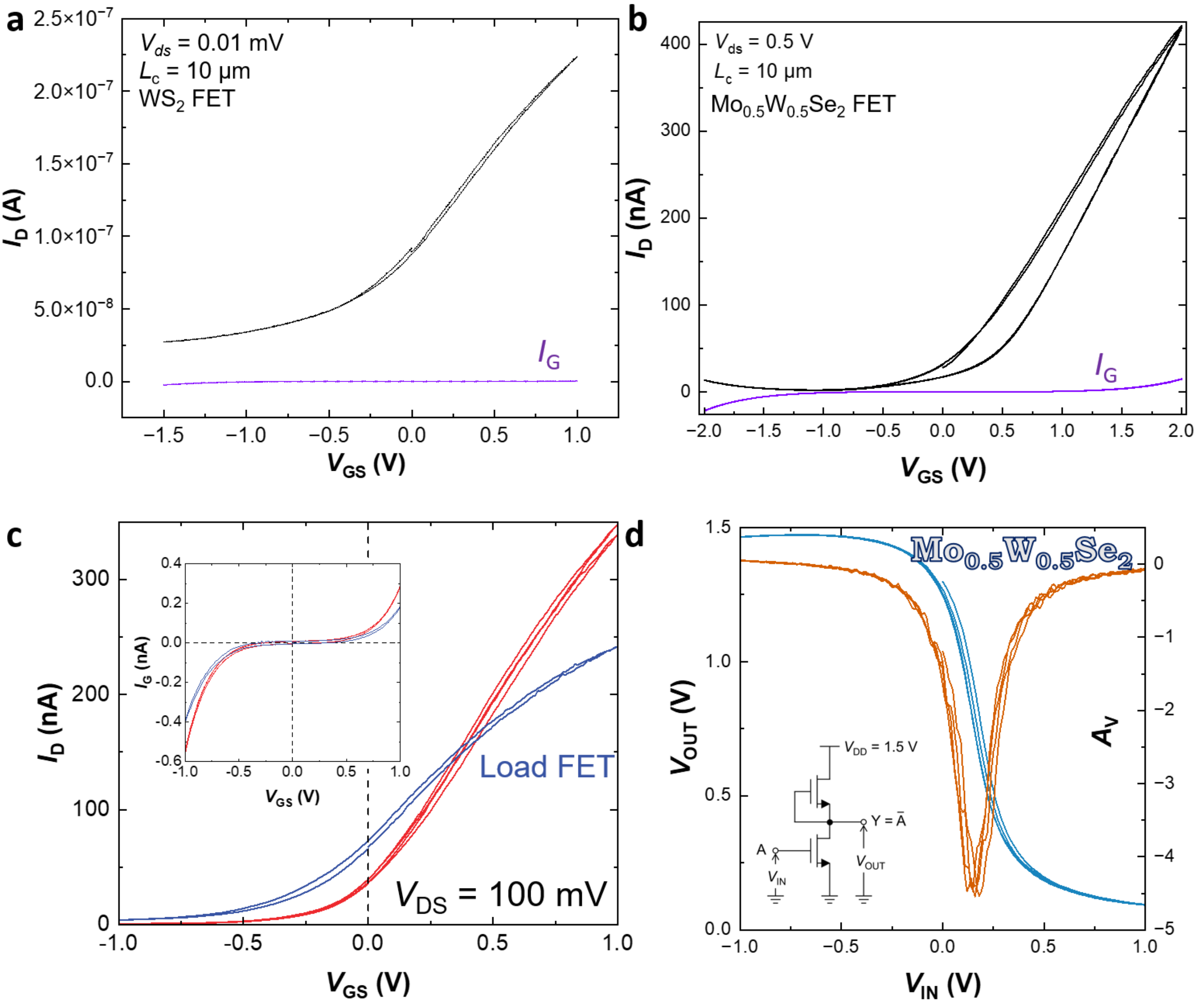

Supplementary Figure 31: **a** Transfer characteristic of WS_2 FET used for the DAC circuits. The gate leakage is shown as the purple line. **b** Transfer characteristic of $Mo_{0.5}W_{0.5}Se_2$ FET used for the NMOS and BASK circuits. The gate leakage is shown as the purple line. **c** Transfer characteristic of the $Mo_{0.5}W_{0.5}Se_2$ driver FET and load FET used in the NMOS circuit. The gate leakage current is shown in the inset. **d** NMOS inverter using solution-processed $Mo_{0.5}W_{0.5}Se_2$ (schematic inset) and it's static voltage transfer characteristic when the supply voltage, V_{DD} = 1.5 V. The blue line shows the input voltage (V_{IN}) versus output voltage (V_{OUT}), with the voltage gain depicted by the orange line.

Digital integrated circuits (IC's) rely on complementary semiconductor materials metal-oxide-semiconductor (MOS) technology that uses p-type (PMOS logic) or n-type (NMOS logic) FETs to implement mixed-signal ICs and logic gates.[103] Figures of merit such as switching speed (τ), and inverter voltage gain ($|A_v|$), defined as the slope of the inverter voltage transfer characteristic (dV_{OUT}/dV_{IN}, where V_{IN} is the input and V_{OUT} the output voltage), have been used to assess and benchmark the performance of the FETs and ICs. Solution-

processed 2D logic has only started within the last few years. The first complementary inverters with solution-processed LPE graphene/hexagonal boron nitride heterostructures achieved an inverter voltage gain, $|A_v| \approx 0.1$ and could not amplify an input voltage, V_{in} since the graphene channel lacked E_g.[104] Lin et al. then demonstrated NMOS with spin-coated MoS_2 achieving $|A_v| \approx 20$.[89] Inkjet-printed CMOS logic was then demonstrated using MoS_2 as an n-type material and organic polymer as a p-type material, achieving $|A_v| \approx 1.4$.[105] This was an essential step as it was the first time an input signal could be successfully amplified in a complementary circuit. The CMOS also had fast $\tau \approx 3.5$ µs that was limited by the poor conductivity of the channel with very high contact resistance, $R_c > 600$ MΩ µm.[105] Ricciardulli et al. demonstrated an all-2D CMOS using drop-cast p-type VP and n-type MoS_2 achieving $|A_v| \approx 17$ however, τ was several milliseconds since ionic liquid was used to gate the devices.[91] More recently, n-type MoS_2 and p-type Br-doped WSe_2 have been drop cast on Si/SiO_2 and patterned by photolithography to achieve a remarkable $|A_v| \approx 1280$ demonstrating that it is possible to make high-performance logic with solution-processed 2D materials.[94] $Mo_{0.5}W_{0.5}Se_2$ FETs have yet to be used to implement solution processed logic devices.

We use e-beam lithography to pattern Ti/Au (3/37 nm) source and drain electrodes and Al (40 nm) gate electrodes onto Si/SiO_2 substrates. A native AlO_x layer, approximately 4 nm thick, formed by exposing Al to ambient air, serves as the gate dielectric in our solid-state FETs. LS deposition is then used to deposit networks ($t \approx 25$ nm) of $Mo_{0.5}W_{0.5}Se_2$ onto the electrodes to complete the device arrays (See Supporting Information S1).

In Supplementary Figure 31a and Supplementary Figure 31b we obtain transfer characteristics of the FETs with channel geometry $L_{CH} = 10$ µm and $W_{CH} = 200$ µm (WS_2) and $W_{CH} = 300$ µm ($Mo_{0.5}W_{0.5}Se_2$) and drain voltage V_{DS} of 0.01 and 0.5 V. Additionally, in Supplementary Figure 31c we show the transfer characteristic of the $Mo_{0.5}W_{0.5}Se_2$ FETs used in the NMOS for the driver and load FETs using $V_{DS} = 0.1$ V. The gate voltage (V_{GS}) is swept at ± 2 V, sufficient to switch the FETs between on and off states due to the high oxide capacitance of the AlO_x dielectric, $C_{ox} \sim 1.4$ µF/cm^2. The transfer curves for the WS_2 and $Mo_{0.5}W_{0.5}Se_2$ devices showed minimal hysteresis, which was attributed to a dielectric/semiconductor interface with minimal trap sites. We observe n-type behaviour for the WS_2 and $Mo_{0.5}W_{0.5}Se_2$. The p-type component of the transfer characteristic for $Mo_{0.5}W_{0.5}Se_2$ is likely at lower $V_{GS} < -2$ V. Since an ultra-thin AlO_x layer of ~ 4 nm is used, it is not possible to sweep to a larger V_{GS} range without increasing the gate leakage beyond I_D. We find $\mu_j \sim 0.1$ cm^2 V^{-1} s^{-1} for the WS_2 and $Mo_{0.5}W_{0.5}Se_2$ FETs, lower than the ionic transistors described in the main text. We attribute the lower μ_j to overestimating the active channel width since the current will only pass through some nanosheets in the network, as shown in our previous works by conductive AFM.[105] Additionally, the FETs likely suffer from a high contact resistance $R_c \sim 1$ MΩ µm as shown with WS_2, $MoSe_2$ and $MoSe_2$:Nb networks in Supplementary Note 23.

We make an n-channel metal–oxide–semiconductor (NMOS) inverter using $Mo_{0.5}W_{0.5}Se_2$ shown in the schematic of Supplementary Figure 31d (inset) using the top FET as a depletion load and the bottom FET as

the driver. The latter actively switches the circuit between its high and low states based on the input voltage, V_{IN}. The static voltage transfer characteristic of the NMOS inverter is shown by the blue line in Supplementary Figure 31d. When the $Mo_{0.5}W_{0.5}Se_2$ gate has V_{IN} lower than the threshold voltage V_{th} of the driver FET, the driver FET is off and the voltage drop across the depletion load is small, thus the output voltage (V_{OUT}) is high ($V_{OUT} \approx V_{DD}$). As V_{IN} increases and exceeds V_{th}, the driver FET turns on and V_{OUT} will drop to the logic low state, achieving signal inversion with a voltage gain $|A_{v,NMOS}| \approx 4$ (orange line), comparable to previous EE MoS_2 NMOS circuits.[89,105]

Supplementary Note 25 | Measurement of circuit time constant τ

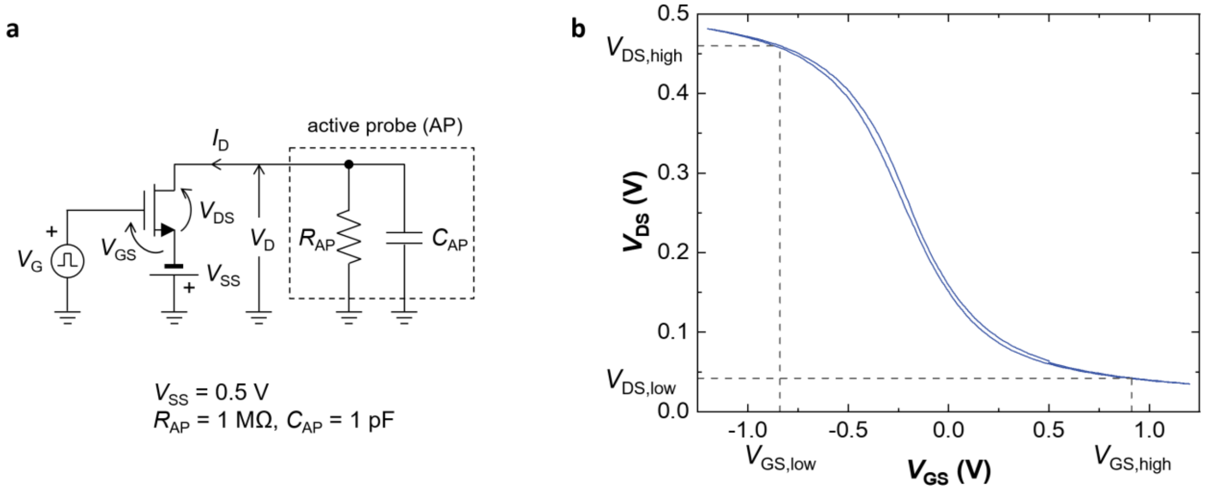

Supplementary Figure 32: **a** Schematic of the circuit to measure the RC time constant with an $Mo_{0.5}W_{0.5}Se_2$ FET. The gate was connected to a function generator providing a square wave gate potential. The AP load is R_{AP} = 1 MΩ with a capacitance, C_{AP} = 1 pF. **b** D.C. transfer curve of the $Mo_{0.5}W_{0.5}Se_2$ FET.

The time constant in a circuit comprising a single $Mo_{0.5}W_{0.5}Se_2$ FET is measured by connecting an active probe (AP) directly to the drain of the FET, as shown in the circuit schematic in Supplementary Figure 32a. The AP functions as a load resistor (R_{AP}) in D.C. conductions. The drain current $I_D = I_R + I_C$, where $I_R = -V_D/R_{AP}$ is the resistive and $I_C = -C_{AP}\, dV_D/dt$ is the capacitive component of the current. I_D can be noisy because of the derivative term therefore it is more practical to use V_{DS} to calculate the time constant, τ. In Supplementary Figure 32b the D.C. transfer curve was derived using the equation $V_{DS} = V_{SS} - R_{AP}{\cdot}I_D$ where V_{SS} = 0.5 V is the supply voltage provided by the Keithley 2636B source-measure unit. $V_{GS,low}$ and $V_{GS,high}$ are the levels of the input square wave where $V_{GS} = V_G + V_{SS}$. The corresponding V_{DS} levels are $V_{DS,high}$ and $V_{DS,low}$ which are the steady-state levels of the V_{DS} vs. time waveforms shown in Supplementary Figure 33.

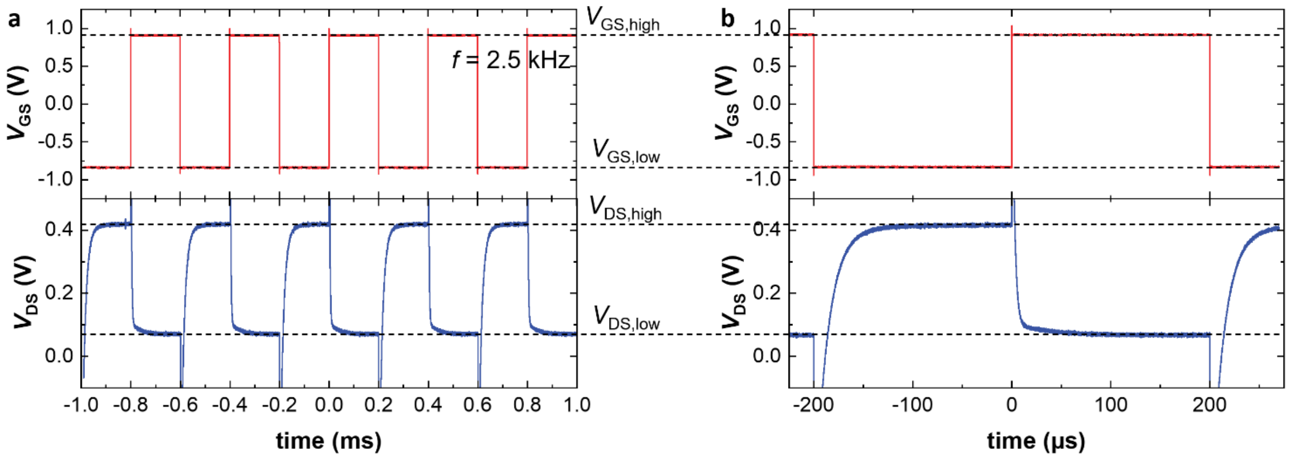

Supplementary Figure 33: **a** Steady-state waveforms of the V_{GS} input (red curve) and V_{DS} (blue curve) at a frequency of 2.5 kHz. **b** Steady-state waveforms of the V_{GS} input with a different time base.

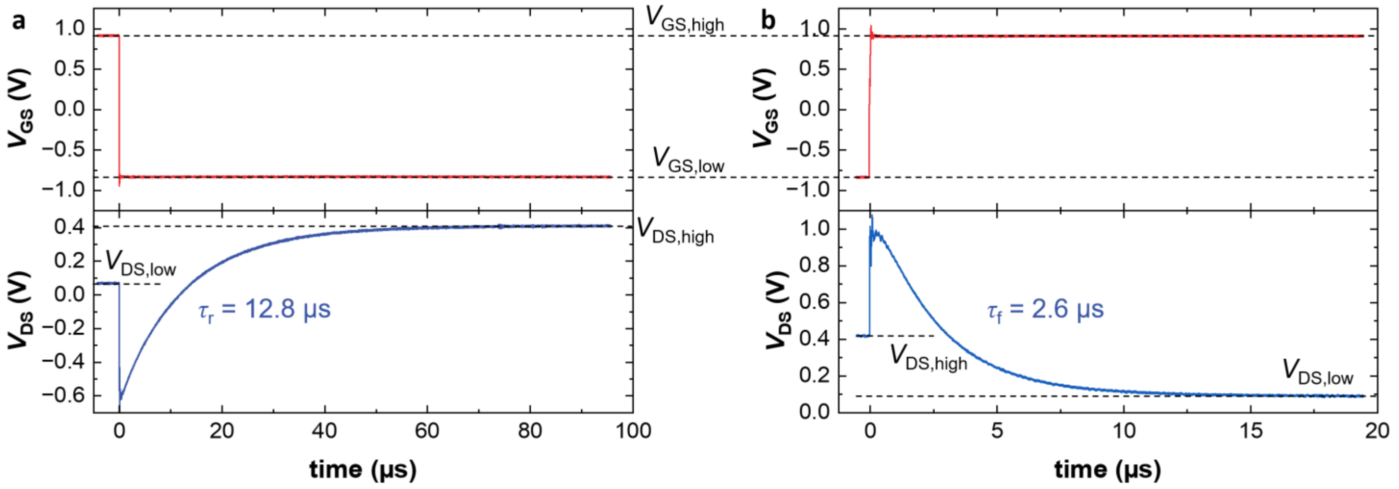

Supplementary Figure 34: The response of V_{DS} to applied V_{GS} as a function of time**. a** The switching time τ_r measured for a rising V_{DS} signal. **b** The switching time τ_f measured for a falling V_{DS} signal.

We magnify the response of V_{DS} in Supplementary Figure 34**.** The rise time of V_{DS} is longer than the fall time, indicating two different switching time constants, τ. From the exponential fit of the rising V_{DS} (Supplementary Figure 34a) and falling V_{DS} (Supplementary Figure 34b) the rising, τ_r and falling, τ_f switching time of the circuit can be found respectively. We find τ_r = 12.8 μs and τ_f = 2.6 μs indicating the fast response of the $Mo_{0.5}W_{0.5}Se_2$ FETs, which is comparable to previously reported EE MoS_2 FETs which achieved τ = 3.3 μs.

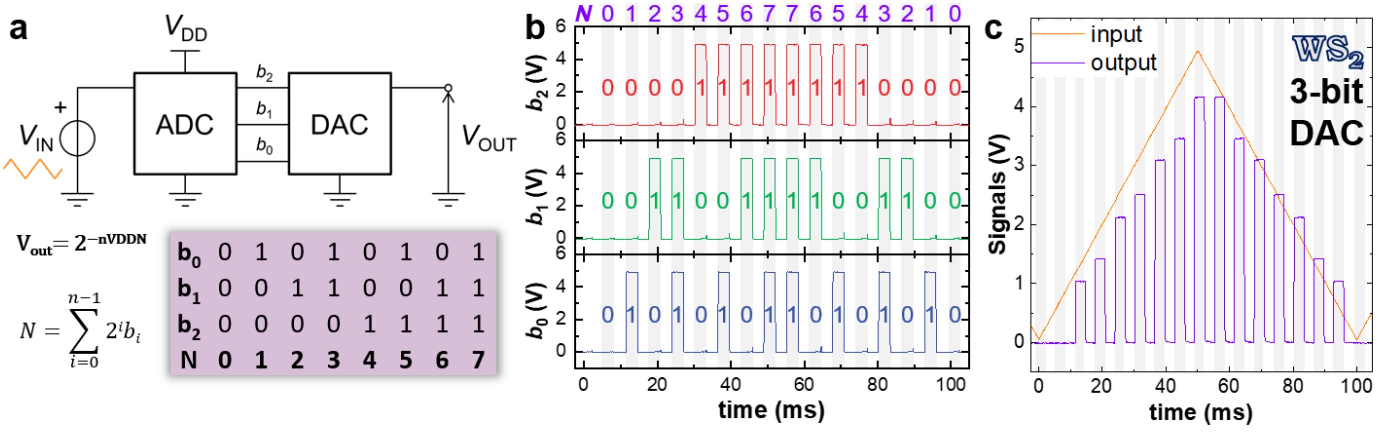

Supplementary Figure 35: **a** Circuit diagram of the analog-to-digital convertor connected with 3 bits to the solution-processed DAC where a logic table is also shown. **b** Digital signals from bit lines in a 3-bit DAC, showing the contribution of each digital signal to the output voltage. **c** Analog output (V_{OUT}) of the WS_2 3-bit DAC compared to the input (V_{IN}) triangle wave, showing pulsed signal due to ADC operation. Analog input to the ADC (orange line) and the corresponding analog output (purple line) from the 3-bit WS_2 DAC.

We use the solution-processed DAC to restore an analogue signal as shown in the circuit diagram of Supplementary Figure 35a. We input an analog triangle wave, V_{IN}, into a conventional Si AD7819 analog-to-digital converter (ADC), which outputs a digital signal of logic "1" or "0" to the WS_2 DAC. We first make a 3-bit DAC and connect the ADC to the DAC using bit lines (b_2, b_1 and b_0), where each bit represents a power of 2 in binary weighting. In our 3-bit DAC, b_2 represents the most significant bit and b_0 represents the least significant bit. We define V_{DD} as the power supply voltage of the ADC. If the value of a bit line is "1", the corresponding ADC output will go to the value of V_{DD} and if it is "0" the output will go to ground. Supplementary Figure 35b shows the digital signals of bits b_2 (red), b_1 (green), and b_0 (blue), and the corresponding binary weighted integer N. We achieve these weights by summating the contribution of each digital signal to the output voltage, V_{OUT}, of the DAC. The contribution of each signal is halved for each node going to the output. For example, in a 3-bit DAC, the contribution of b_2 is halved once, b_1 twice and b_0 three times. The formula for the output voltage considering each bit can influence the voltage by its weighted proportion of the total V_{DD} is, $V_{OUT} = 2^{-n} \times V_{DD} \times N$, where n is the number of bit lines. For our 3-bit DAC, $n = 3$ and V_{DD} is set to 5 V.

In Supplementary Figure 35c, we show the V_{IN} (orange) to the ADC and the analog output of the WS_2 DAC (purple line). V_{OUT} is a pulsed signal due to the operation of the ADC. The ADC performs a digital conversion at the beginning of each non-gray interval. During this time, the ADC is busy, and its digital outputs are at 0 V, hence V_{OUT} = 0 V**.** We set the sampling period to 6.25 ms to get 16 sampling intervals within 100 ms, which is the period of the input triangle wave (orange line). Since the sampling is done asynchronously with respect to the input signal, V_{OUT} is offset in time to V_{IN}.

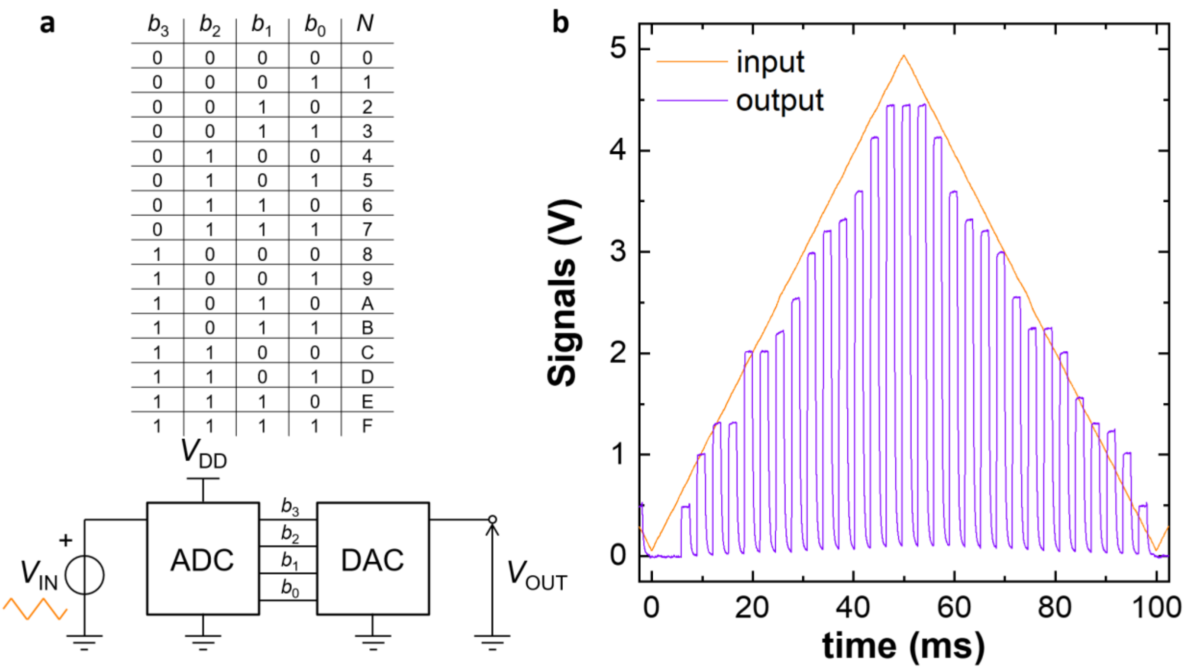

b_3	b_2	b_1	b_0	N
0	0	0	0	0
0	0	0	1	1
0	0	1	0	2
0	0	1	1	3
0	1	0	0	4
0	1	0	1	5
0	1	1	0	6
0	1	1	1	7
1	0	0	0	8
1	0	0	1	9
1	0	1	0	A
1	0	1	1	B
1	1	0	0	C
1	1	0	1	D
1	1	1	0	E
1	1	1	1	F

Supplementary Figure 36: **a** Circuit diagram of the analog-to-digital convertor connected with 4 bits to the solution-processed DAC where a logic table is also shown where V_{DD} = 5 V. **b** Analog output of the WS_2 4-bit DAC compared to the input triangle wave, showing pulsed signal due to ADC operation. Analog input to the ADC (orange line) and the corresponding analog output (purple line) from the 4-bit WS_2 DAC.

We make solution processed 4-bit WS_2 DACs to build on the work of the 3-bit DAC's presented in the main text. In Supplementary Figure 36a we show the circuit diagram of the 4-bit DAC adding one extra binary weighted resistor to give 16 discrete V_{OUT} values shown in the logic table (hexadecimal encoding is used for N). In Supplementary Figure 36b, we show the V_{IN} (orange) to the ADC and the analog output of the WS_2 DAC (purple line). V_{OUT} is a pulsed signal due to the operation of the ADC. We set the sampling period to 3.125 ms to get 32 sampling intervals within 100 ms, which is the period of the input triangle wave (orange line). Since the sampling is done asynchronously with respect to the input signal, V_{OUT} is offset in time to V_{IN}. The quantisation is not perfectly symmetrical in the 4-bit DAC, which we attribute to the network's non-identical WS_2 resistances between semiconductor channels.

Supplementary Note 28 | Amplitude Demodulation of BASK Signal

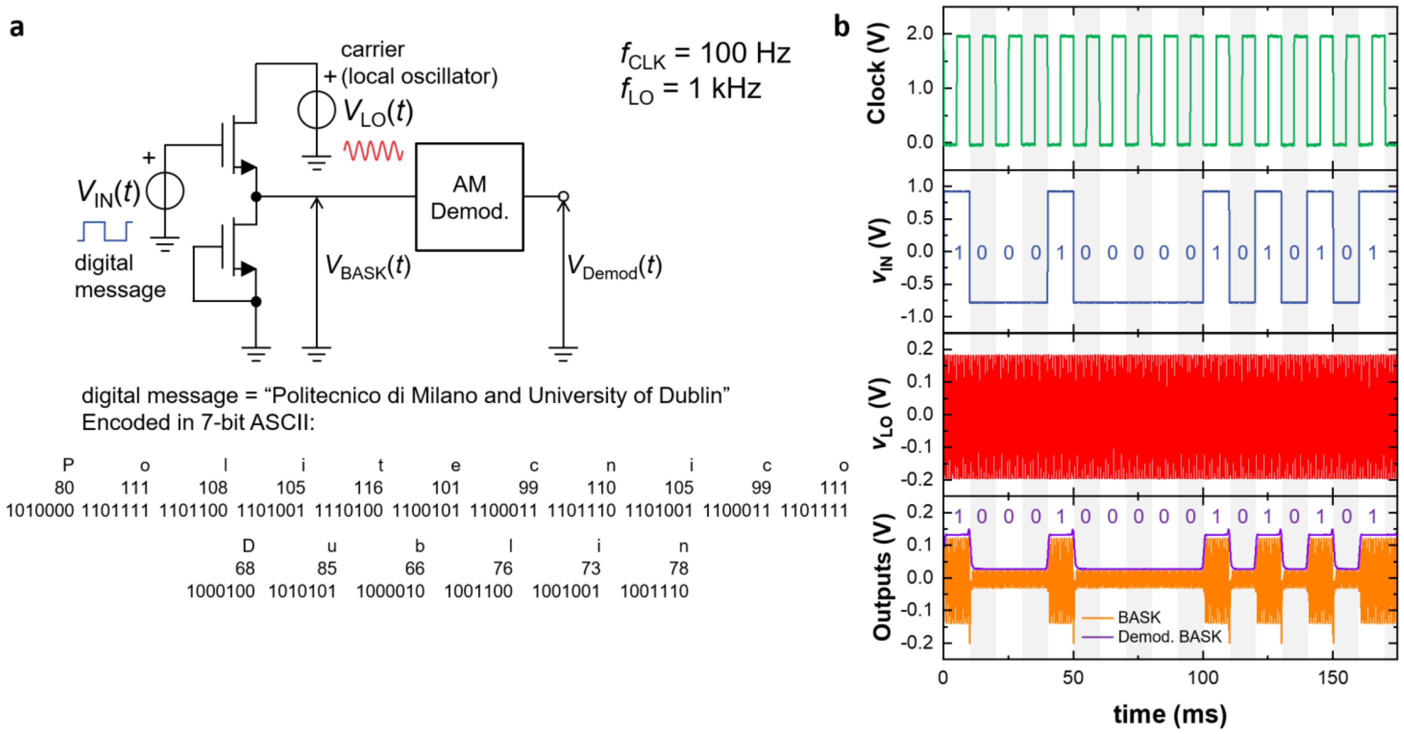

Supplementary Figure 37: **a** Circuit diagram of the $Mo_{0.5}W_{0.5}Se_2$ solution processed BASK circuit (top) and example binary input required to encode "Politecnico" and "Dublin" in 7-bit ASCII (bottom). **b** The clock signal with a frequency of f_{CLK} = 100 Hz is shown by the green curve, while the V_{IN} represents the digital message shown by the blue curve. The local oscillator V_{LO} is shown in red. The output of the BASK circuit V_{BASK} which has the encoded digital message in the local oscillator is shown by the orange curve and the demodulated BASK signal V_{Demod} is shown as the purple line.

We expand our work on $Mo_{0.5}W_{0.5}Se_2$ FETs to implement BASK circuits to modulate the amplitude of a high-frequency signal by a binary signal obtained by encoding a message. The modulated signal is demodulated using an amplitude modulation (AM) demodulator. The circuit diagram is shown in Supplementary Figure 37a. ASCII is a standard way of representing characters as binary numbers. As an example, Supplementary Figure 37a shows the binary bits required to encode "Politecnico" and "Dublin".The digital message "Politecnico di Milano and University of Dublin" was encoded using 7-bit ASCII. Since each character is represented by 7 bits and the message has 46 characters, the total number of bits sent is 46 characters × 7 bits/character = 322 bits. The encoded binary sequence (322 bits) of high "1" and low "0" is input as V_{IN} to the top FET of the BASK modulator. The green curve in Supplementary Figure 37b represents the clock signal with a frequency of f_{CLK} = 100 Hz which each period corresponds to the transmission of one bit of data. Therefore we have a data transmission rate of 100 bits a second (bps). We provide a high-frequency (1 kHz) AC sine wave, with an amplitude of 200 mV, to act as a carrier signal denoted as the local oscillator, V_{LO}. The BASK circuit then converts the binary sequency into an AM signal previously represented as V_{OUT}

which we will now call V_{BASK} shown as the orange curve of Supplementary Figure 37b. V_{BASK} is input into the AM demodulator and the output voltage is denoted as V_{Demod} shown by the purple line of Supplementary Figure 37b. Supplementary Figure 37b (orange and purple curves) shows a short part (175 milliseconds) of the modulated and demodulated signal. The entire message takes 3.22 seconds to transmit (322 bits/100 bps) and the snippet of the signal shows how the signal behaves to transmit a space and the letter "U".

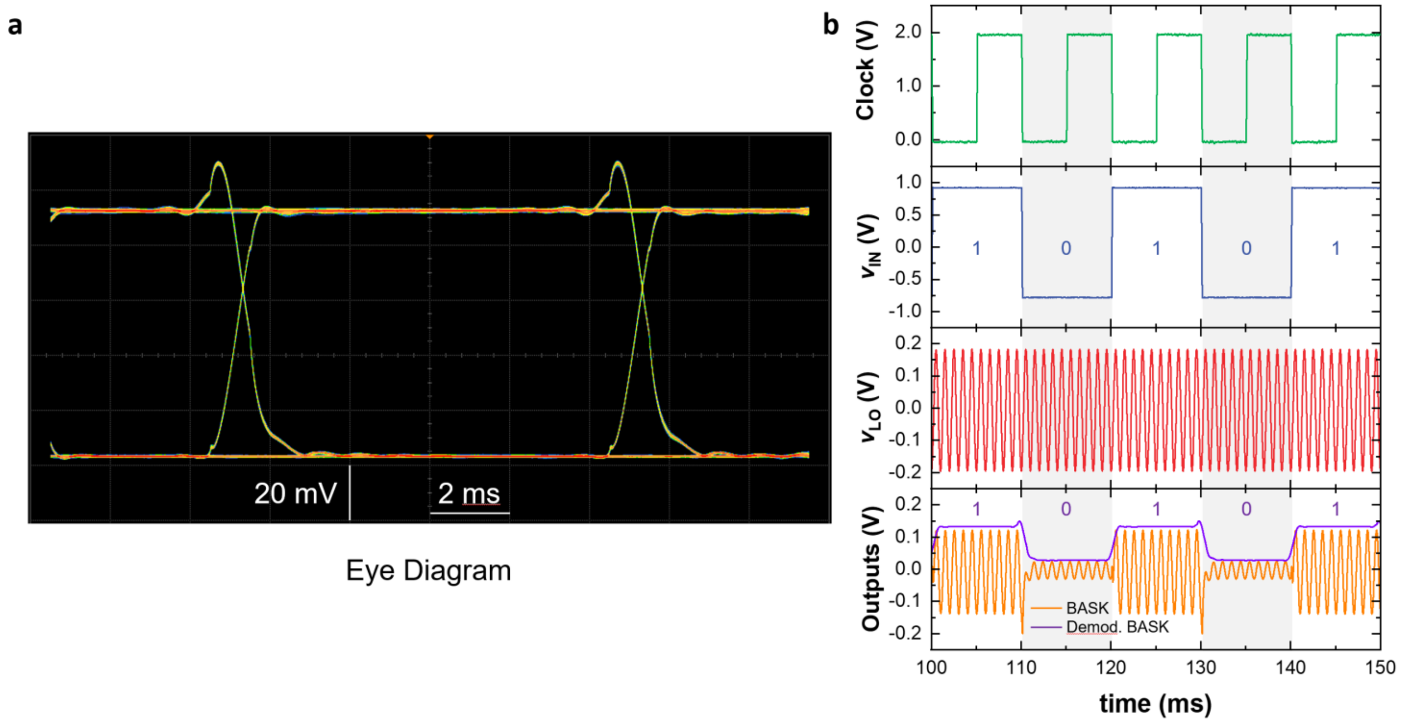

Supplementary Figure 38: **a** Eye diagram of the demodulated BASK signal, the horizontal axis represents time (2 ms/div), while the vertical axis represents the demodulated signal (20 mV/div). **b** The clock signal (green), V_{IN} (blue), V_{LO} (red), V_{BASK} (orange) and V_{Demod} (purple) at a shorter time window than in Supplementary Figure 37 using the same device.

We show an eye diagram in Supplementary Figure 38a which is tool typically used in digital communications to visualize how well a digital signal can be interpreted by the receiver. Supplementary Figure 38a is obtained from the entire modulated message (all 322 bits). Supplementary Figure 38b shows the same plot and device as Supplementary Figure 37 but with a time window between 100 and 150 ms to see the demodulated signal more clearly. The clear, open eye pattern in Supplementary Figure 38a indicates a good quality signal with minimal interference and noise.

Supplementary Note 29 | Literature Review of Solution Processed 2D Materials

Deposition Method	Material	Annealing Temperature (°C)	Acid Treatment (TFSI)	Measurement Environment	Mobility $cm^2V^{-1}s^{-1}$	I_{on}/I_{off}	Year	Method	Ref
Spin Coating	MoS_2 rGO	50 C (overnight)	No	Ambient	0.3	<2	2012	LPE	[106]
Inkjet Printing	MoS_2	450C 1 hours	No	Passivation (ALD 50nm Al2O3)	0.00048	3	2014	LPE	[107]
Spin Coating TiO2/drop casting MoS2	MoS_2	250C for TiO2 - 25 C	No	10^{-6} mBar (for 12 h)	0.02	nan	2014	LPE	[108]
Dip-Coating	MoS_2	350 C 1 hour	Yes hydrazine hydrate	Ambient	0.4	10^6	2015	Chem-ical	[109]
Inkjet/Spray/Evaporation	MoS_2/WS_2/WSe_2/$MoSe_2$	70 C 24 hours	No	Under Vacuum (1 x 10-4 mbar)	0.1	100	2017	LPE	[110]
Inkjet Printing	Graphene, hBN	100C 1 hour	no	Ambient Air	204	2.5	2017	LPE	[104]
Drop-casting	Few-layer BP	250 (in forming gas H_2/Ar)	Not specified	Ambient	Up to 100 (60 on average), as single nanosheets (not a network)	~1 × 10^4 (avg.)	2018	EE	[111]
Spin-coating	MoS_2	300 C	Yes	Vacuum	7-11	10^6	2018	EE	[89]
Spray	WS_2	100C for 12h	No	Under Vacuum <10^{-6} mbar	0.01	10^4	2018	LPE	[112]
Spray Coating	MoS_2	200C for 2 hours	No	nan	0.0004	22	2018	LPE	[113]
Spray Coating	InSe	200 °C for 30 minutes	Not specified	Under vacuum	3 × 10^{-5}	3	2020	na	[114]
Liquid-Liquid Assembly	MoS_2	200 C for 2 hours	No	Ambient	0.73	10^5	2020	EE	[79]

Spray Coating + ebeam evaporation	WSe_2	80C + 70C overnight	No	Under Vacuum	0.08 (WSe2), 0.1 (WS2)	10^3	2020	LPE	[115]
Electrohydrodynamic Jet	MoS_2	150 (pre-annealing), 1000	No	Not mentioned	19.4	5.0×10^6	2021	Pre-cursur solution	[116]
Unknown	In_2Se_3	400C	Not specified	In vacuum and dark	0.2	10^5	2021	EE	[117]
Inkjet Printing	MoS_2	200 (in air) followed by 300 (in argon atmosphere	Not specified	Ambient	Subthermionic: Not specified; Thermionic: 7-11	Subthermionic: up to 10^6; Thermionic: up to 10^6	2021	Chem-ical	[118]
Spray Coating	GaSe	Not Specified	Not Applicable	Ambient	$\approx10^{-3}$	$\approx10^3$	2021	LPE	[119]
Spin-coating	MoS_2	250C 1.5h + 400C 2 hours	nan	Vacuum	5	10^6	2021	EE	[120]
Inkjet Printing + ALD + e-beam	MoS_2	400C 1 hour	No	Ambient	0.1	25	2021	EE	[121]
Inkjet Printing + ALD + e-beam	MoS_2	400C 1 hour	Yes	Ambient	0.06	50	2021	EE	[105]
Drop Casting - MoS2/ Dry transfer from PET	MoS_2	110C + 90C for 30min	Yes, saturated solution of BDT	Under Vacuum <10^−6 mbar	0.01	10^4	2021	LPE	[122]
Spin Coating + photolithography	MoS_2	80C	Yes (TFSI)	Under Vacuum (~7 × 10-5 torr)	1.8	10^6	2021	EE	[123]
Spin-Coated	MoS_2	1000 °C for 1h	No	(~10-2 Torr) growth only	7.9	10^5	2021	Chem-ical	[124]
Spin Coating + wet transfer + inkjet printing - Inkjet not used for dielectric	MoS_2/HfS_2	500C for 5h (HfO2), 80C for 5min (MoS2), 100C for 5min (TFSI).	Yes (TFSI)	All under vacuum (10-5 Torr)	8.3	10^6	2021	EE	[125]

		300C for 30min + 200C for 30min							
Inkjet Printing	MoS_2	500 (for HfO_2)	Yes (TFSI for MoS2)	Not mentioned	~10	>10^5	2022	EE	[126]
Layer-by-Layer (LbL) assembly	In_2Se_3	200°C (Vacuum for 2 hours before the test)	Not mentioned directly	Not mentioned directly	Single nanosheet: 12.8 cm²/Vs, Thin film: 0.4 cm²/Vs	Single nanosheet: > 1.5 × 10^3, Thin film: 7 × 10^4	2022	EE	[127]
Drop-casting	Violet Phosphorus	Not specified	Not specified	Not specified	2.25	10^4	2022	LPE	[91]
Direct Writing	MoS_2	300	Not applicable	Ambient	Up to 6.7	2 × 10^6	2022	EE	[128]
Spin coating	Graphene, MoS_2, HfO_2 from HfS_2	500 (for HfS_2 to HfO_2 conversion)	Yes	Room temperature, under vacuum	8.3 for MoS_2 devices	1.6 × 10^6 for MoS_2 devices	2022	EE	[125]
Screen Printing	WSe_2	Not Applicable	Not Applicable	Room Temperature	0.066	≈2000	2022	LPE	[129]
Langmuir Shafer/Stamping	MoS_2	200C for 2 hours	no	Under N2	0.2	14	2022	EE with Powder	[130]
Spin-coated Mos2+wet transfer	MoS_2	300C	no	no	10	100	2022	EE	[131]
Vacuum filtration transfer	BP	nan	nan	nan	0.002	130	2022	EE	[92]
Slot-Die Coating	MoS_2	250	Yes	Not Mentioned	~112	>10^5	2023	EE	[132]
Inkjet Printing	MoS_2 & Graphene	350	Not explicitly mentioned but TFSI treatment discussed	Ambient	≈0.27 (for AlOx/Si substrate), potentially higher due to actual channel	Up to 10^3 (AlOx/Si substrate)	2023	EE	[133]

			as a method for enhancing mobility		width considerations				
Drop-casting	WSe_2, MoS_2 (for complementary circuits)	200 (for annealing in a nitrogen-filled glovebox)	Not specified	Measurements in an N2-filled glovebox at 25°C, bias stress measurement at 60°C under vacuum	>27	~10^7	2023	EE	[94]
Unknown, likely dropcast	$PtSe_2$	Not specified	Not specified	Vacuum at room temperature	0.0004 (n-type behavior attributed to Se vacancies)	6.3 × 10^3	2023	EE	[90]
Langmuir–Schaefer coating	MoS_2, WS_2, WSe_2	120	Not used	Ambient	MoS_2: ≈ 11, WS_2: ≈ 9, WSe_2: ≈ 2	MoS_2: ≈ 2.6 × 10^3, WS_2: ≈ 3.4 × 10^3, WSe_2: ≈ 4.2 × 10^4	2023	EE	[67]
Drop-casting	WSe_2	Not directly mentioned; annealed at 80°C for FeCl3 doping	FeCl3 (p-type doping)	Ambient	~1.5 after FeCl3 doping	~10^6	2023	EE	[93]
Inkjet Printed MoS2 and ionic gel	MoS_2	200C 1 hour, Ar atmosphere at 120°C	Yes (TFSI, 80C 1 hour)	No	11	10^6	2023	EE	[134]
Drop-casting	$MoSe_2$	120-180	No	N_2-filled glovebox	1.5	>10^6	2024	EE	[135]
Drop-casting	WS_2	120-180	No	N_2-filled glovebox	0.6	>10^6	2024	EE	[135]
Not mentioned	WSe_2	Not mentioned	Not mentioned	Not mentioned	0.002	~10^2	2024	EE	[135]

Not mentioned	MoS_2	Not mentioned	Yes	Not mentioned	0.5	3	2024	EE	[135]
Langmuir−Blodgett and spin-coating	MoS_2 and $Sr_{1.8}Bi_{0.2}Nb_3O_{10}$	Below 250	Yes, bis(trifluoromethane)sulfonimide (TFSI) treatment	Ambient air	Average 4.4, Maximum 11	$>10^5$	2024	EE	[136]
Drop Casting	MoS_2	15 min at 120 °C in an Ar glovebox	no	Ambient Air	15	26	2024	EE	[80]
Drop Casting	WSe_2	15 min at 120 °C in an Ar glovebox	no	Ambient Air	1	10^4	2024	EE	[80]
Langmuir−Schaefer coating	MoS_2	150	No	Ambient	≈ 7	10^3	2024	EE	[102]

Supplementary Note 30 | Supplementary References

1 Gates-Rector, S. & Blanton, T. The Powder Diffraction File: a quality materials characterization database. *Powder Diffraction* **34**, 352-360 (2019). https://doi.org/10.1017/S0885715619000812

2 Holder, C. F. & Schaak, R. E. Tutorial on Powder X-ray Diffraction for Characterizing Nanoscale Materials. *ACS Nano* **13**, 7359-7365 (2019). https://doi.org/10.1021/acsnano.9b05157

3 Faita, F. L., Campos, C. E. M., Ersching, K. & Pizani, P. S. Structural, thermal and vibrational characterization of mechanical alloyed In50Te50. *Materials Chemistry and Physics* **125**, 257-262 (2011). https://doi.org/10.1016/j.matchemphys.2010.09.020

4 Kang, J., Tongay, S., Li, J. & Wu, J. Monolayer semiconducting transition metal dichalcogenide alloys: Stability and band bowing. *Journal of Applied Physics* **113** (2013). https://doi.org/10.1063/1.4799126

5 Choudhary, K. *et al.* The joint automated repository for various integrated simulations (JARVIS) for data-driven materials design. *npj Computational Materials* **6**, 173 (2020). https://doi.org/10.1038/s41524-020-00440-1

6 Talirz, L. *et al.* Materials Cloud, a platform for open computational science. *Sci Data* **7**, 299 (2020). https://doi.org/10.1038/s41597-020-00637-5

7 Wei, Q. *et al.* Quasi-Two-Dimensional Se-Terminated Bismuth Oxychalcogenide (Bi(2)O(2)Se). *ACS Nano* **13**, 13439-13444 (2019). https://doi.org/10.1021/acsnano.9b07000

8 Backes, C. *et al.* Equipartition of Energy Defines the Size-Thickness Relationship in Liquid-Exfoliated Nanosheets. *ACS Nano* **13**, 7050-7061 (2019). https://doi.org/10.1021/acsnano.9b02234

9 Mounet, N. *et al.* Two-dimensional materials from high-throughput computational exfoliation of experimentally known compounds. *Nat Nanotechnol* **13**, 246-252 (2018). https://doi.org/10.1038/s41565-017-0035-5

10 Campi, D., Mounet, N., Gibertini, M., Pizzi, G. & Marzari, N. Expansion of the Materials Cloud 2D Database. *ACS Nano* **17**, 11268-11278 (2023). https://doi.org/10.1021/acsnano.2c11510

11 Giannozzi, P. *et al.* QUANTUM ESPRESSO: a modular and open-source software project for quantum simulations of materials. *J Phys Condens Matter* **21**, 395502 (2009). https://doi.org/10.1088/0953-8984/21/39/395502

12 Giannozzi, P. *et al.* Advanced capabilities for materials modelling with Quantum ESPRESSO. *J Phys Condens Matter* **29**, 465901 (2017). https://doi.org/10.1088/1361-648X/aa8f79

13 Prandini, G., Marrazzo, A., Castelli, I. E., Mounet, N. & Marzari, N. Precision and efficiency in solid-state pseudopotential calculations. *npj Computational Materials* **4**, 72 (2018). https://doi.org/10.1038/s41524-018-0127-2

14 Perdew, J. P., Burke, K. & Ernzerhof, M. Generalized Gradient Approximation Made Simple. *Phys Rev Lett* **77**, 3865-3868 (1996). https://doi.org/10.1103/PhysRevLett.77.3865

15 Hamada, I. & Otani, M. Comparative van der Waals density-functional study of graphene on metal surfaces. *Physical Review B* **82**, 153412 (2010). https://doi.org/10.1103/PhysRevB.82.153412

16 Vydrov, O. A. & Van Voorhis, T. Nonlocal van der Waals density functional made simple. *Phys Rev Lett* **103**, 063004 (2009). https://doi.org/10.1103/PhysRevLett.103.063004

17 Marzari, N., Vanderbilt, D., De Vita, A. & Payne, M. C. Thermal Contraction and Disordering of the Al(110) Surface. *Physical Review Letters* **82**, 3296-3299 (1999). https://doi.org/10.1103/PhysRevLett.82.3296

18 Dal Corso, A. Elastic constants of beryllium: a first-principles investigation. *J Phys Condens Matter* **28**, 075401 (2016). https://doi.org/10.1088/0953-8984/28/7/075401

19 Lei, S. *et al.* Evolution of the electronic band structure and efficient photo-detection in atomic layers of InSe. *ACS Nano* **8**, 1263-1272 (2014). https://doi.org/10.1021/nn405036u

20 Marvan, P., Mazanek, V. & Sofer, Z. Shear-force exfoliation of indium and gallium chalcogenides for selective gas sensing applications. *Nanoscale* **11**, 4310-4317 (2019). https://doi.org/10.1039/c8nr09294j

21 Chitara, B. & Ya'akobovitz, A. High-frequency electromechanical resonators based on thin GaTe. *Nanotechnology* **28**, 42LT02 (2017). https://doi.org/10.1088/1361-6528/aa897d

22 Fonseca, J. J. *et al.* Bandgap Restructuring of the Layered Semiconductor Gallium Telluride in Air. *Adv Mater* **28**, 6465-6470 (2016). https://doi.org/10.1002/adma.201601151

23 Mutlu, Z. *et al.* Phase Engineering of 2D Tin Sulfides. *Small* **12**, 2998-3004 (2016). https://doi.org/10.1002/smll.201600559

24 Efthimiopoulos, I. *et al.* Effects of temperature and pressure on the optical and vibrational properties of thermoelectric SnSe. *Phys Chem Chem Phys* **21**, 8663-8678 (2019). https://doi.org/10.1039/c9cp00897g

25 Lin, S. *et al.* Accessing valley degree of freedom in bulk Tin(II) sulfide at room temperature. *Nat Commun* **9**, 1455 (2018). https://doi.org/10.1038/s41467-018-03897-3
26 Norton, K. J., Alam, F. & Lewis, D. J. A Review of the Synthesis, Properties, and Applications of Bulk and Two-Dimensional Tin (II) Sulfide (SnS). *Applied Sciences* **11**, 2062 (2021).
27 Plechinger, G. *et al.* Raman spectroscopy of the interlayer shear mode in few-layer MoS2 flakes. *Applied Physics Letters* **101**, 101906 (2012). https://doi.org/10.1063/1.4751266
28 Fujisawa, K. *et al.* Quantification and Healing of Defects in Atomically Thin Molybdenum Disulfide: Beyond the Controlled Creation of Atomic Defects. *ACS Nano* **15**, 9658-9669 (2021). https://doi.org/10.1021/acsnano.0c10897
29 Taghizadeh, A., Leffers, U., Pedersen, T. G. & Thygesen, K. S. A library of ab initio Raman spectra for automated identification of 2D materials. *Nat Commun* **11**, 3011 (2020). https://doi.org/10.1038/s41467-020-16529-6
30 Zhang, R., Drysdale, D., Koutsos, V. & Cheung, R. Controlled Layer Thinning and p-Type Doping of WSe2 by Vapor XeF2. *Adv. Funct. Mater.* **27**, 1702455 (2017). https://doi.org/10.1002/adfm.201702455
31 Zhao, W. *et al.* Lattice dynamics in mono- and few-layer sheets of WS2 and WSe2. *Nanoscale* **5**, 9677-9683 (2013). https://doi.org/10.1039/c3nr03052k
32 Dey, S. & Singh, G. Sodium and potassium ion storage in cation substituted 2D MoWSe2: insights into the effects of upper voltage cut-off. *Nanotechnology* **34**, 385401 (2023). https://doi.org/10.1088/1361-6528/acdf66
33 Wang, F. *et al.* Strain-induced phonon shifts in tungsten disulfide nanoplatelets and nanotubes. *2D Mater.* **4**, 015007 (2016). https://doi.org/10.1088/2053-1583/4/1/015007
34 Ruppert, C., Aslan, B. & Heinz, T. F. Optical properties and band gap of single- and few-layer MoTe2 crystals. *Nano Lett* **14**, 6231-6236 (2014). https://doi.org/10.1021/nl502557g
35 Li, J.-H. *et al.* Thickness-dependent excitonic properties of atomically thin 2H-MoTe2*. *Chinese Physics B* **29**, 017802 (2020). https://doi.org/10.1088/1674-1056/ab5a3a
36 Er, E. *et al.* High-Yield Preparation of Exfoliated 1T-MoS2 with SERS Activity. *Chemistry of Materials* **31**, 5725-5734 (2019). https://doi.org/10.1021/acs.chemmater.9b01698
37 Han, A. *et al.* One-step synthesis of single-site vanadium substitution in 1T-WS2 monolayers for enhanced hydrogen evolution catalysis. *Nat. Commun* **12**, 709 (2021). https://doi.org/10.1038/s41467-021-20951-9
38 Calandra, M. Chemically exfoliated single-layer ${\text{MoS}}_{2}$: Stability, lattice dynamics, and catalytic adsorption from first principles. *Phys. Rev. B* **88**, 245428 (2013). https://doi.org/10.1103/PhysRevB.88.245428
39 Sokolikova, M. S., Sherrell, P. C., Palczynski, P., Bemmer, V. L. & Mattevi, C. Direct solution-phase synthesis of 1T' WSe2 nanosheets. *Nat. Commun* **10**, 712 (2019). https://doi.org/10.1038/s41467-019-08594-3
40 Fraser, J. P. *et al.* Selective phase growth and precise-layer control in MoTe2. *Communications Materials* **1**, 48 (2020). https://doi.org/10.1038/s43246-020-00048-4
41 Gupta, U. *et al.* Characterization of few-layer 1T-MoSe2 and its superior performance in the visible-light induced hydrogen evolution reaction. *APL Materials* **2** (2014). https://doi.org/10.1063/1.4892976
42 Parhizkar, S. *et al.* Two-Dimensional Platinum Diselenide Waveguide-Integrated Infrared Photodetectors. *ACS Photonics* **9**, 859-867 (2022). https://doi.org/10.1021/acsphotonics.1c01517
43 Sojková, M. *et al.* High carrier mobility epitaxially aligned PtSe2 films grown by one-zone selenization. *Applied Surface Science* **538**, 147936 (2021). https://doi.org/10.1016/j.apsusc.2020.147936
44 Lukas, S. *et al.* Correlating Nanocrystalline Structure with Electronic Properties in 2D Platinum Diselenide. *Advanced Functional Materials* **31**, 2102929 (2021). https://doi.org/10.1002/adfm.202102929
45 Hajiyev, P., Cong, C., Qiu, C. & Yu, T. Contrast and Raman spectroscopy study of single- and few-layered charge density wave material: 2H-TaSe(2). *Sci Rep* **3**, 2593 (2013). https://doi.org/10.1038/srep02593
46 Hirata, T. & Ohuchi, F. S. Temperature dependence of the Raman spectra of 1T-TaS2. *Solid State Communications* **117**, 361-364 (2001). https://doi.org/10.1016/s0038-1098(00)00468-3
47 Garcia-Vidal, F. J. & Pendry, J. B. Collective Theory for Surface Enhanced Raman Scattering. *Phys Rev Lett* **77**, 1163-1166 (1996). https://doi.org/10.1103/PhysRevLett.77.1163
48 Smith, A. J., Meek, P. E. & Liang, W. Y. Raman scattering studies of SnS2and SnSe2. *Journal of Physics C: Solid State Physics* **10**, 1321-1323 (1977). https://doi.org/10.1088/0022-3719/10/8/035
49 Biswas, R. *et al.* Strong near band-edge excited second-harmonic generation from multilayer 2H Tin diselenide. *Sci Rep* **11**, 15017 (2021). https://doi.org/10.1038/s41598-021-94612-8
50 Surrente, A. *et al.* Excitons in atomically thin black phosphorus. *Physical Review B* **93**, 121405 (2016). https://doi.org/10.1103/PhysRevB.93.121405

51 Liang, S., Hasan, M. N. & Seo, J.-H. Direct Observation of Raman Spectra in Black Phosphorus under Uniaxial Strain Conditions. *Nanomaterials* **9** (2019).
52 Khatun, S., Banerjee, A. & Pal, A. J. Nonlayered tellurene as an elemental 2D topological insulator: experimental evidence from scanning tunneling spectroscopy. *Nanoscale* **11**, 3591-3598 (2019). https://doi.org/10.1039/c8nr09760g
53 Yadav, R. A. *et al.* Anomalous vibrational behavior of two dimensional tellurium: Layer thickness and temperature dependent Raman spectroscopic study. *Applied Surface Science* **531**, 147303 (2020). https://doi.org/10.1016/j.apsusc.2020.147303
54 Zwick, A., Renucci, M. A. & Kjekshus, A. Raman scattering in the IVB transition-metal trichalcogenides: ZrS3, ZrSe3, ZrTe3and HfSe3. *Journal of Physics C: Solid State Physics* **13**, 5603-5614 (1980). https://doi.org/10.1088/0022-3719/13/30/023
55 Kargar, F. *et al.* Phonon and Thermal Properties of Quasi-Two-Dimensional FePS(3) and MnPS(3) Antiferromagnetic Semiconductors. *ACS Nano* **14**, 2424-2435 (2020). https://doi.org/10.1021/acsnano.9b09839
56 Kuo, C. T. *et al.* Exfoliation and Raman Spectroscopic Fingerprint of Few-Layer NiPS3 Van der Waals Crystals. *Sci Rep* **6**, 20904 (2016). https://doi.org/10.1038/srep20904
57 Liu, Q. *et al. Probing quasi-long-range ordering by magnetostriction in monolayer CoPS3*. (2021).
58 Backes, C. *et al.* Edge and confinement effects allow in situ measurement of size and thickness of liquid-exfoliated nanosheets. *Nat Commun* **5**, 4576 (2014). https://doi.org/10.1038/ncomms5576
59 Hanlon, D. *et al.* Liquid exfoliation of solvent-stabilized few-layer black phosphorus for applications beyond electronics. *Nat Commun* **6**, 8563 (2015). https://doi.org/10.1038/ncomms9563
60 Sang, D. K. *et al.* Electronic and Optical Properties of Two-Dimensional Tellurene: From First-Principles Calculations. *Nanomaterials* **9** (2019).
61 Shang, J. *et al.* Tunable electronic and optical properties of InSe/InTe van der Waals heterostructures toward optoelectronic applications. *Journal of Materials Chemistry C* **6**, 7201-7206 (2018). https://doi.org/10.1039/c8tc01533c
62 Zhang, M. *et al.* Group IIIA/IVA monochalcogenides nanosheets for ultrafast photonics. *APL Photonics* **4** (2019). https://doi.org/10.1063/1.5100848
63 Shah, M. S. *et al.* Thickness-Dependent Physical Properties of Tin Sulfide Thin Films for an Efficient Sunlight-Absorbing Layer. *Journal of Electronic Materials* **51**, 6454-6462 (2022). https://doi.org/10.1007/s11664-022-09881-4
64 Heo, S. H. *et al.* Composition change-driven texturing and doping in solution-processed SnSe thermoelectric thin films. *Nat Commun* **10**, 864 (2019). https://doi.org/10.1038/s41467-019-08883-x
65 Castellanos-Gomez, A., Quereda, J., van der Meulen, H. P., Agrait, N. & Rubio-Bollinger, G. Spatially resolved optical absorption spectroscopy of single- and few-layer MoS(2) by hyperspectral imaging. *Nanotechnology* **27**, 115705 (2016). https://doi.org/10.1088/0957-4484/27/11/115705
66 Dong, N. *et al.* Optical Limiting and Theoretical Modelling of Layered Transition Metal Dichalcogenide Nanosheets. *Sci Rep* **5**, 14646 (2015). https://doi.org/10.1038/srep14646
67 Carey, T. *et al.* High-Mobility Flexible Transistors with Low-Temperature Solution-Processed Tungsten Dichalcogenides. *ACS Nano* **17**, 2912-2922 (2023). https://doi.org/10.1021/acsnano.2c11319
68 Backes, C. *et al.* Production of Highly Monolayer Enriched Dispersions of Liquid-Exfoliated Nanosheets by Liquid Cascade Centrifugation. *ACS Nano* **10**, 1589-1601 (2016). https://doi.org/10.1021/acsnano.5b07228
69 Gong, Y. *et al.* Two-Dimensional Platinum Diselenide: Synthesis, Emerging Applications, and Future Challenges. *Nanomicro Lett* **12**, 174 (2020). https://doi.org/10.1007/s40820-020-00515-0
70 Ji, X. *et al.* Integration of functionalized two-dimensional TaS2 nanosheets and an electron mediator for more efficient biocatalyzed artificial photosynthesis. *Journal of Materials Chemistry A* **5**, 5511-5522 (2017). https://doi.org/10.1039/c7ta00002b
71 Kumaar Swamy Reddy, B., Veeralingam, S., Borse, P. H. & Badhulika, S. A flexible, rapid response, hybrid inorganic–organic SnSe2–PEDOT:PSS bulk heterojunction based high-performance broadband photodetector. *Materials Chemistry Frontiers* **6**, 341-351 (2022). https://doi.org/10.1039/D1QM01232K
72 Eda, G. & Maier, S. A. Two-dimensional crystals: managing light for optoelectronics. *ACS Nano* **7**, 5660-5665 (2013). https://doi.org/10.1021/nn403159y
73 Gao, Y. *et al.* Bias-switchable negative and positive photoconductivity in 2D FePS(3) ultraviolet photodetectors. *Nanotechnology* **29**, 244001 (2018). https://doi.org/10.1088/1361-6528/aab9d2

74 Liu, J. *et al.* NiPS(3) nanoflakes: a nonlinear optical material for ultrafast photonics. *Nanoscale* **11**, 14383-14391 (2019). https://doi.org/10.1039/c9nr03964c

75 Pacilé, D. *et al.* Photoemission and optical studies ofZrSe3,HfSe3, andZrS3. *Physical Review B* **76**, 155406 (2007). https://doi.org/10.1103/PhysRevB.76.155406

76 Yu, J. *et al.* Ternary SnS(2-x)Se(x) Alloys Nanosheets and Nanosheet Assemblies with Tunable Chemical Compositions and Band Gaps for Photodetector Applications. *Sci Rep* **5**, 17109 (2015). https://doi.org/10.1038/srep17109

77 Horan, P. & Blau, W. Optical nonlinearity near the bandgap in semiconductors. *Contemporary Physics* **28**, 59-68 (1987). https://doi.org/10.1080/00107518708211040

78 Kelly, A. G., O'Suilleabhain, D., Gabbett, C. & Coleman, J. N. The electrical conductivity of solution-processed nanosheet networks. *Nature Reviews Materials* **7**, 217-234 (2021). https://doi.org/10.1038/s41578-021-00386-w

79 Neilson, J., Avery, M. P. & Derby, B. Tiled Monolayer Films of 2D Molybdenum Disulfide Nanoflakes Assembled at Liquid/Liquid Interfaces. *ACS Appl Mater Interfaces* **12**, 25125-25134 (2020). https://doi.org/10.1021/acsami.0c03794

80 Carey, T. *et al.* Knot Architecture for Biocompatible and Semiconducting 2D Electronic Fiber Transistors. *Small Methods*, e2301654 (2024). https://doi.org/10.1002/smtd.202301654

81 Backes, C. *et al.* Spectroscopic metrics allow in situ measurement of mean size and thickness of liquid-exfoliated few-layer graphene nanosheets. *Nanoscale* **8**, 4311-4323 (2016). https://doi.org/10.1039/c5nr08047a

82 Wu, W., Qiu, G., Wang, Y., Wang, R. & Ye, P. Tellurene: its physical properties, scalable nanomanufacturing, and device applications. *Chem Soc Rev* **47**, 7203-7212 (2018). https://doi.org/10.1039/c8cs00598b

83 Gao, M. *et al.* One-Pot Hydrothermal Synthesis of Thin Tellurene Nanosheet and Its Formation Mechanism. *Journal of Nanomaterials* **2019**, 1-7 (2019). https://doi.org/10.1155/2019/5715291

84 Shi, Z. *et al.* Two-Dimensional Tellurium: Progress, Challenges, and Prospects. *Nanomicro Lett* **12**, 99 (2020). https://doi.org/10.1007/s40820-020-00427-z

85 Wang, N. Z. *et al.* Tunable superconductivity by electrochemical intercalation in TaS2. *New Journal of Physics* **20**, 023014 (2018). https://doi.org/10.1088/1367-2630/aaa8a7

86 Yan-Bin, Q., Yan-Ling, L., Guo-Hua, Z., Zhi, Z. & Xiao-Ying, Q. Anisotropic properties of TaS2. *Chinese Physics* **16**, 3809-3814 (2007). https://doi.org/10.1088/1009-1963/16/12/042

87 Xia, X., Sun, X., Wang, H. & Li, X. Introducing Electrode Contact by Controlled Micro-Alloying in Few-Layered GaTe Field Effect Transistors. *Crystals* **10** (2020).

88 Gholamvand, Z., McAteer, D., Harvey, A., Backes, C. & Coleman, J. N. Electrochemical Applications of Two-Dimensional Nanosheets: The Effect of Nanosheet Length and Thickness. *Chemistry of Materials* **28**, 2641-2651 (2016). https://doi.org/10.1021/acs.chemmater.6b00009

89 Lin, Z. *et al.* Solution-processable 2D semiconductors for high-performance large-area electronics. *Nature* **562**, 254-258 (2018). https://doi.org/10.1038/s41586-018-0574-4

90 Cho, Y. S. *et al.* Electronic and electrocatalytic applications based on solution-processed two-dimensional platinum diselenide with thickness-dependent electronic properties. *EcoMat* **5**, e12358 (2023). https://doi.org/https://doi.org/10.1002/eom2.12358

91 Ricciardulli, A. G., Wang, Y., Yang, S. & Samorì, P. Two-Dimensional Violet Phosphorus: A p-Type Semiconductor for (Opto)electronics. *Journal of the American Chemical Society* **144**, 3660-3666 (2022). https://doi.org/10.1021/jacs.1c12931

92 Jeon, Y. *et al.* Electrochemically exfoliated phosphorene nanosheet thin films for wafer-scale near-infrared phototransistor array. *npj 2D Materials and Applications* **6**, 82 (2022). https://doi.org/10.1038/s41699-022-00360-2

93 Zou, T. *et al.* Charge transfer enabled by the p-doping of WSe2 for 2D material-based printable electronics. *Journal of Information Display* **24**, 255-261 (2023). https://doi.org/10.1080/15980316.2023.2204205

94 Zou, T. *et al.* High-Performance Solution-Processed 2D P-Type WSe2 Transistors and Circuits through Molecular Doping. *Advanced Materials* **35**, 2208934 (2023). https://doi.org/https://doi.org/10.1002/adma.202208934

95 Suh, J. *et al.* Doping against the native propensity of MoS2: degenerate hole doping by cation substitution. *Nano Lett* **14**, 6976-6982 (2014). https://doi.org/10.1021/nl503251h

96 McCandless, T. E., Ruiz, J. & Campbell, A. R. Rhenium behavior in molybdenite in hypogene and near-surface environments: Implications for Re-Os geochronometry. *Geochimica et Cosmochimica Acta* **57**, 889-905 (1993). https://doi.org/10.1016/0016-7037(93)90176-w
97 Torsi, R. *et al.* Dilute Rhenium Doping and its Impact on Defects in MoS2. *ACS Nano* **17**, 15629-15640 (2023). https://doi.org/10.1021/acsnano.3c02626
98 Ansari, L. *et al.* Quantum confinement-induced semimetal-to-semiconductor evolution in large-area ultra-thin PtSe2 films grown at 400 °C. *npj 2D Materials and Applications* **3**, 33 (2019). https://doi.org/10.1038/s41699-019-0116-4
99 Xia, F., Wang, H. & Jia, Y. Rediscovering black phosphorus as an anisotropic layered material for optoelectronics and electronics. *Nat Commun* **5**, 4458 (2014). https://doi.org/10.1038/ncomms5458
100 Huang, Y. *et al.* Interaction of Black Phosphorus with Oxygen and Water. *Chemistry of Materials* **28**, 8330-8339 (2016). https://doi.org/10.1021/acs.chemmater.6b03592
101 Xu, W., Gan, L., Wang, R., Wu, X. & Xu, H. Surface Adsorption and Vacancy in Tuning the Properties of Tellurene. *ACS Appl Mater Interfaces* **12**, 19110-19115 (2020). https://doi.org/10.1021/acsami.9b21625
102 Gabbett, C. *et al.* Understanding how junction resistances impact the conduction mechanism in nano-networks. *Nature Communications* **15**, 4517 (2024). https://doi.org/10.1038/s41467-024-48614-5
103 Voinigescu, S. *High-Frequency Integrated Circuits*. (2013).
104 Carey, T. *et al.* Fully inkjet-printed two-dimensional material field-effect heterojunctions for wearable and textile electronics. *Nat Commun* **8**, 1202 (2017). https://doi.org/10.1038/s41467-017-01210-2
105 Carey, T. *et al.* Inkjet Printed Circuits with 2D Semiconductor Inks for High-Performance Electronics. *Advanced Electronic Materials* **7**, 2100112 (2021). https://doi.org/10.1002/aelm.202100112
106 He, Q. *et al.* Fabrication of flexible MoS2 thin-film transistor arrays for practical gas-sensing applications. *Small* **8**, 2994-2999 (2012). https://doi.org/10.1002/smll.201201224
107 Li, J., Naiini, M. M., Vaziri, S., Lemme, M. C. & Östling, M. Inkjet Printing of MoS2. *Advanced Functional Materials* **24**, 6524-6531 (2014). https://doi.org/10.1002/adfm.201400984
108 Yu, X., Prévot, M. S. & Sivula, K. Multiflake Thin Film Electronic Devices of Solution Processed 2D MoS2 Enabled by Sonopolymer Assisted Exfoliation and Surface Modification. *Chemistry of Materials* **26**, 5892-5899 (2014). https://doi.org/10.1021/cm502378g
109 Xi, Y. *et al.* Fabrication of MoS2 thin film transistors via selective-area solution deposition methods. *Journal of Materials Chemistry C* **3**, 3842-3847 (2015). https://doi.org/10.1039/c5tc00062a
110 Kelly, A. G. *et al.* All-printed thin-film transistors from networks of liquid-exfoliated nanosheets. *Science* **356**, 69-73 (2017). https://doi.org/10.1126/science.aal4062
111 Li, J. *et al.* Ultrafast Electrochemical Expansion of Black Phosphorus toward High-Yield Synthesis of Few-Layer Phosphorene. *Chemistry of Materials* **30**, 2742-2749 (2018). https://doi.org/10.1021/acs.chemmater.8b00521
112 Higgins, T. M. *et al.* Electrolyte-Gated n-Type Transistors Produced from Aqueous Inks of WS2 Nanosheets. *Advanced Functional Materials* **29**, 1804387 (2018). https://doi.org/10.1002/adfm.201804387
113 Zeng, X., Hirwa, H., Metel, S., Nicolosi, V. & Wagner, V. Solution processed thin film transistor from liquid phase exfoliated MoS2 flakes. *Solid-State Electronics* **141**, 58-64 (2018). https://doi.org/10.1016/j.sse.2017.12.005
114 Curreli, N. *et al.* Liquid Phase Exfoliated Indium Selenide Based Highly Sensitive Photodetectors. *Advanced Functional Materials* **30**, 1908427 (2020). https://doi.org/10.1002/adfm.201908427
115 O'Suilleabhain, D. *et al.* Effect of the Gate Volume on the Performance of Printed Nanosheet Network-Based Transistors. *ACS Applied Electronic Materials* **2**, 2164-2170 (2020). https://doi.org/10.1021/acsaelm.0c00368
116 Can, T. T. T., Kwack, Y.-J. & Choi, W.-S. Drop-on-demand patterning of MoS2 using electrohydrodynamic jet printing for thin-film transistors. *Materials & Design* **199**, 109408 (2021). https://doi.org/10.1016/j.matdes.2020.109408
117 Lin, Z. *et al.* High-yield exfoliation of 2D semiconductor monolayers and reassembly of organic/inorganic artificial superlattices. *Chem* **7**, 1887-1902 (2021). https://doi.org/10.1016/j.chempr.2021.03.022
118 Mondal, S. K., Biswas, A., Pradhan, J. R. & Dasgupta, S. Inkjet-Printed MoS(2) Transistors with Predominantly Intraflake Transport. *Small Methods* **5**, e2100634 (2021). https://doi.org/10.1002/smtd.202100634
119 Curreli, N. *et al.* Liquid-Phase Exfoliated Gallium Selenide for Light-Driven Thin-Film Transistors. *Advanced Electronic Materials* **7**, 2001080 (2021). https://doi.org/10.1002/aelm.202001080
120 Ma, C. *et al.* Two-dimensional van der Waals thin film transistors as active matrix for spatially resolved pressure sensing. *Nano Research* **14**, 3395-3401 (2021). https://doi.org/10.1007/s12274-021-3717-0
121 Piatti, E. *et al.* Charge transport mechanisms in inkjet-printed thin-film transistors based on two-dimensional materials. *Nature Electronics* **4**, 893-905 (2021). https://doi.org/10.1038/s41928-021-00684-9

122 Ippolito, S. *et al.* Covalently interconnected transition metal dichalcogenide networks via defect engineering for high-performance electronic devices. *Nat Nanotechnol* **16**, 592-598 (2021). https://doi.org/10.1038/s41565-021-00857-9

123 Kim, J. *et al.* Area-Selective Chemical Doping on Solution-Processed MoS2 Thin-Film for Multi-Valued Logic Gates. *Nano Letters* **22**, 570-577 (2022). https://doi.org/10.1021/acs.nanolett.1c02947

124 Kwack, Y.-J., Can, T. T. T. & Choi, W.-S. Bottom-up water-based solution synthesis for a large MoS2 atomic layer for thin-film transistor applications. *npj 2D Materials and Applications* **5**, 84 (2021). https://doi.org/10.1038/s41699-021-00264-7

125 Kim, J. *et al.* All-Solution-Processed Van der Waals Heterostructures for Wafer-Scale Electronics. *Advanced Materials* **34**, 2106110 (2022). https://doi.org/https://doi.org/10.1002/adma.202106110

126 Song, O. *et al.* All inkjet-printed electronics based on electrochemically exfoliated two-dimensional metal, semiconductor, and dielectric. *npj 2D Materials and Applications* **6**, 64 (2022). https://doi.org/10.1038/s41699-022-00337-1

127 Gao, X. *et al.* Thin-Film Transistors from Electrochemically Exfoliated In2Se3 Nanosheets. *Micromachines* **13** (2022).

128 Li, L. *et al.* Interface Capture Effect Printing Atomic-Thick 2D Semiconductor Thin Films. *Advanced Materials* **34**, 2207392 (2022). https://doi.org/https://doi.org/10.1002/adma.202207392

129 Abdolhosseinzadeh, S. *et al.* A Universal Approach for Room-Temperature Printing and Coating of 2D Materials. *Adv Mater* **34**, e2103660 (2022). https://doi.org/10.1002/adma.202103660

130 Wells, R. A. *et al.* High Performance Semiconducting Nanosheets via a Scalable Powder-Based Electrochemical Exfoliation Technique. *ACS Nano* **16**, 5719-5730 (2022). https://doi.org/10.1021/acsnano.1c10739

131 Yan, Z. *et al.* Highly stretchable van der Waals thin films for adaptable and breathable electronic membranes. *Science* **375**, 852-859 (2022). https://doi.org/doi:10.1126/science.abl8941

132 Kwon, Y. A. *et al.* Wafer-scale transistor arrays fabricated using slot-die printing of molybdenum disulfide and sodium-embedded alumina. *Nature Electronics* **6**, 443-450 (2023). https://doi.org/10.1038/s41928-023-00971-7

133 Sui, X. *et al.* Fully Inkjet-Printed, 2D Materials-Based Field-Effect Transistor for Water Sensing. *Advanced Materials Technologies* **8**, 2301288 (2023). https://doi.org/https://doi.org/10.1002/admt.202301288

134 Chen, M. *et al.* Inkjet-Printed MoS2 Nanoplates on Flexible Substrates for High-Performance Field Effect Transistors and Gas Sensing Applications. *ACS Applied Nano Materials* **6**, 3236-3244 (2023). https://doi.org/10.1021/acsanm.2c04885

135 Zou, T. *et al.* Electrical Properties of Electrochemically Exfoliated 2D Transition Metal Dichalcogenides Transistors for Complementary Metal-Oxide-Semiconductor Electronics. *Advanced Electronic Materials* **10**, 2300691 (2024). https://doi.org/https://doi.org/10.1002/aelm.202300691

136 Joung, S.-Y. *et al.* All-Solution-Processed High-Performance MoS2 Thin-Film Transistors with a Quasi-2D Perovskite Oxide Dielectric. *ACS Nano* **18**, 1958-1968 (2024). https://doi.org/10.1021/acsnano.3c06972
